# Enantioselective bromination of axially chiral cyanoarenes in the presence of bifunctional organocatalysts†

**DOI:** 10.1039/c9ra05532k

**Published:** 2019-10-04

**Authors:** Yuuki Wada, Akira Matsumoto, Keisuke Asano, Seijiro Matsubara

**Affiliations:** Department of Material Chemistry, Graduate School of Engineering, Kyoto University Kyotodaigaku-Katsura, Nishikyo Kyoto 615-8510 Japan asano.keisuke.5w@kyoto-u.ac.jp matsubara.seijiro.2e@kyoto-u.ac.jp +81 75 383 2438 +81 75 383 7571 +81 75 383 7130

## Abstract

Enantioselective bromination of axially chiral cyanoarenes bearing high intrinsic rotational barriers *via* dynamic kinetic resolution using bifunctional organocatalysts is reported. Sequential addition of a brominating reagent in several portions at an optimized temperature was effective in accomplishing high enantioselectivities.

## Abstract 2

Enantioselective bromination of axially chiral cyanoarenes bearing high intrinsic rotational barriers *via* dynamic kinetic resolution using bifunctional organocatalysts is reported.

## Graphical abstract

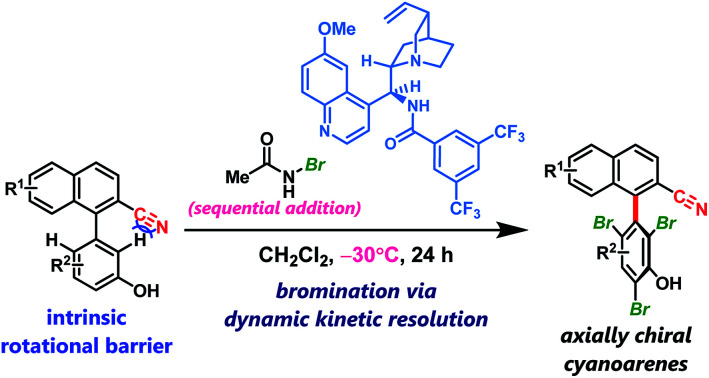

Axially chiral biaryls are privileged structures in pharmaceuticals,^1^ asymmetric catalysts,^2^ functional materials,^3^*etc.* Thus, the development of efficient methods for their synthesis is desirable to advance research in these scientific fields. Among recent accomplishments on catalytic atroposelective transformations^4^ toward the synthesis of densely substituted axially chiral biaryls, powerful strategies include organocatalytic dynamic kinetic resolution involving *ortho*-functionalization of existing biaryls *via* the introduction of additional rotational barriers.^5^ In this method, substrates should in principle have rotational barriers low enough to enable fast rotation about the biaryl axis, leading to their rapid racemization. Therefore, it is difficult to employ biaryl substrates bearing intrinsic rotational barriers, which impede their racemization. In 2015, the Miller group reported an elegant example of this type of dynamic kinetic resolution *via* bromination of 3-arylquinazolin-4(3*H*)-ones, the rotational barrier of which is ∼19 kcal mol^−1^, by slow addition of a brominating agent.^5*e*^ Here, we present enantioselective bromination of axially chiral cyanoarenes bearing intrinsic rotational barriers exceeding 18 kcal mol^−1^ (Scheme 1). To the best of our knowledge, there has been no report of a catalytic asymmetric reaction affording axially chiral cyanoarenes,^6^ despite their prevalence in bioactive agents^7^ and the rich chemistry of cyano compounds as synthetic intermediates.^8^

**Scheme 1:**
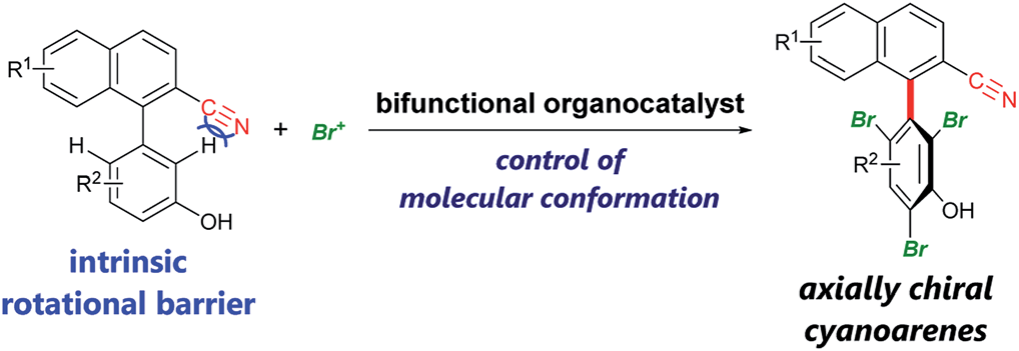
Enantioselective bromination of axially chiral cyanoarenes using bifunctional organocatalysts.

Table 1 shows the optimization of reaction conditions. We started our investigation using 1-(3-hydroxyphenyl)-2-naphthonitrile (1a) and *N*-bromoacetamide (NBA, 4a) as the brominating reagent with 10 mol% quinine-derived bifunctional catalysts 3a–3c in CH_2_Cl_2_ at 25 °C. Urea and amide catalysts 3a and 3c, respectively, afforded 2a in higher enantioselectivities than the thiourea catalyst 3b (Table 1, entries 1–3).^9^ Other catalysts 3d and 3e, bearing a cyclohexanediamine framework, and 3f, bearing a binaphthyl framework, resulted in poor enantioselectivities (Table 1, entries 4–6).^10^ Using 3a and 3c, lower reaction temperatures were investigated (Table 1, entries 7–10); 3c gave higher enantioselectivity at −40 °C, although the reactions did not proceed at all at −60 °C. By screening different solvents, CH_2_Cl_2_ was identified as the most suitable solvent from the viewpoints of both yield and enantioselectivity (Table 1, entries 9 and 11–16). Other brominating reagents (Fig. 1) were also investigated; NBA (4a) still afforded the best enantioselectivities (Table 1, entries 9 and 17–19). The decrease in the loading of 3c to 5 mol% slightly improved the enantioselectivity, although a longer reaction time (48 h) was necessary (Table 1, entry 20). Next, using the thus-optimized conditions, reactions with shorter reaction times (24 h, 12 h, and 6 h) were carried out (Table 1, entries 21–23); the enantioselectivity was improved while the conversions decreased with decreasing reaction time. These results imply that the racemization of 1a at −40 °C is not rapid enough to make use of dynamic kinetic resolution.

**Table 1:** Optimization of conditions^a^

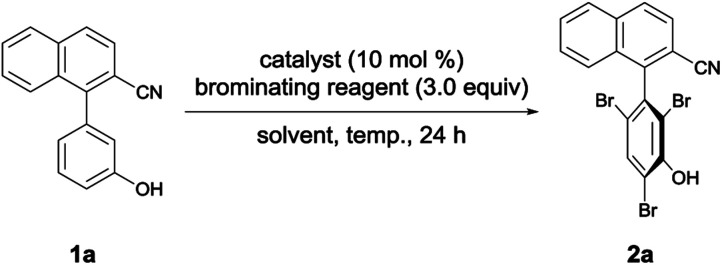						
Entry	Catalyst	Brominating reagent	Solvent	Temp. (°C)	Yield^b^ (%)	ee (%)
1	3a	NBA (4a)	CH_2_Cl_2_	25	81	26
2	3b	NBA (4a)	CH_2_Cl_2_	25	87	6
3	3c	NBA (4a)	CH_2_Cl_2_	25	89	18
4	3d	NBA (4a)	CH_2_Cl_2_	25	83	6
5	3e	NBA (4a)	CH_2_Cl_2_	25	85	3
6	3f	NBA (4a)	CH_2_Cl_2_	25	79	3
7	3a	NBA (4a)	CH_2_Cl_2_	−40	82	20
8	3a	NBA (4a)	CH_2_Cl_2_	−60	<1	—
9	3c	NBA (4a)	CH_2_Cl_2_	−40	83	41
10	3c	NBA (4a)	CH_2_Cl_2_	−60	<1	—
11	3c	NBA (4a)	CHCl_3_	−40	21	54
12	3c	NBA (4a)	Toluene	−40	18	−10
13	3c	NBA (4a)	THF	−40	13	−2
14	3c	NBA (4a)	Et_2_O	−40	46	−20
15	3c	NBA (4a)	EtOAc	−40	68	−4
16	3c	NBA (4a)	EtOH	−40	<5	—
17	3c	DBH (4b)	CH_2_Cl_2_	−40	79	1
18	3c	NBS (4c)	CH_2_Cl_2_	−40	82	20
19	3c	NBP (4d)	CH_2_Cl_2_	−40	79	−5
20^c^^,^^d^	3c	NBA (4a)	CH_2_Cl_2_	−40	85	49
21^c^^,^^e^	3c	NBA (4a)	CH_2_Cl_2_	−40	51	59
22^c^^,^^f^	3c	NBA (4a)	CH_2_Cl_2_	−40	33	66
23^c^^,^^g^	3c	NBA (4a)	CH_2_Cl_2_	−40	17	71
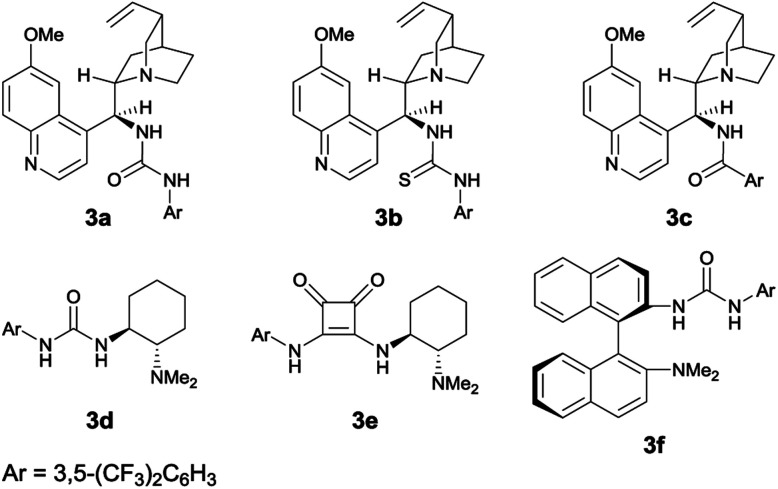						

a Reactions were run using 1a (0.10 mmol), the brominating reagent (0.30 mmol), and the catalyst (0.010 mmol) in the solvent (10 mL).

b Isolated yields.

c Reactions were run using 3c (0.0050 mmol).

d Reaction was run for 48 h.

e Reaction was run for 24 h.

f Reaction was run for 12 h.

g Reaction was run for 6 h.

**Fig. 1:**
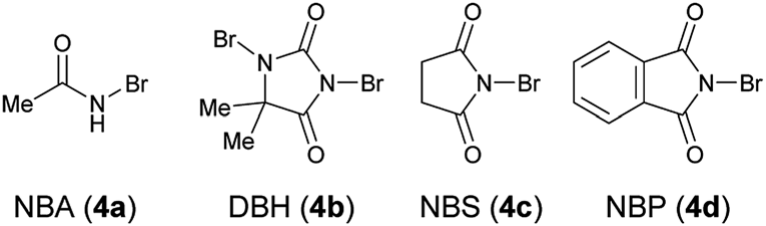
Brominating reagents.

Subsequently, to improve the efficiency of dynamic kinetic resolution by retarding the enantiodetermining bromination,^5*e*^4a was added sequentially in five portions (Fig. 2).^11^ Although the procedure hardly affected the results at −40 °C, the enantioselectivity was greatly improved for reactions carried out at −20 °C and −30 °C. Such effects were smaller at temperatures above −10 °C.

**Fig. 2:**
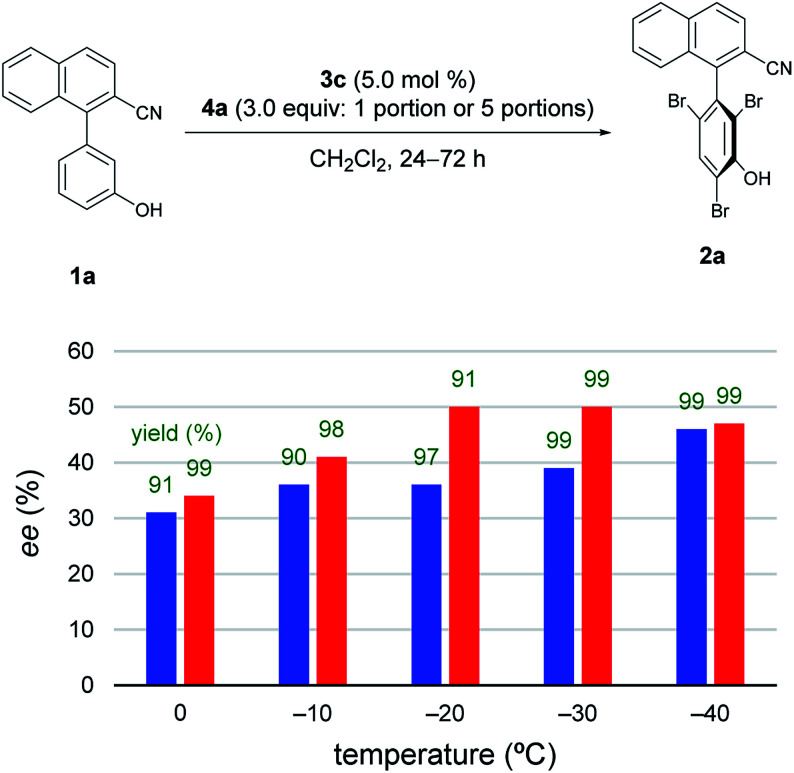
Investigations of temperatures and procedures. Blue bar: reactions were run with 4a added in 1 portion. Red bar: reactions were run with 4a added in 5 portions. Green values represent yields of 2a isolated after silica gel column chromatography. At 0, −10, −20, and −30 °C, reactions were run for 24 h; at −40 °C, reactions were run for 72 h.

Next, at −30 °C and −40 °C, respectively, the relationships between enantioselectivity and yield were investigated (Fig. 3). Reactions were carried out using various amounts of 4a. At both temperatures, the enantioselectivity decreased as the yield increased; however, the quantitative reactions also exhibited some enantioselectivity (−30 °C: 99% yield, 50% ee; −40 °C: 99% yield, 47% ee), implying the presence of the characteristics of dynamic kinetic resolution. In addition, although the enantioselectivity was better at −40 °C than −30 °C when the yield was low, the relationship became reversed as the yield increased; hence, the efficiency of dynamic kinetic resolution was revealed to be better at −30 °C than at −40 °C. Furthermore, when 1.5 equiv. of 4a were used at −30 °C affording 2a in 22% yield with 65% ee, the *ortho*-monobrominated product 1a-Br (Fig. 4) was also obtained with 75% ee (see Scheme S1 in the ESI† for details). It shows that the bromination at one of the *ortho*-positions introduces a rotational barrier high enough to set the chiral axis, which is consistent with the rotational barriers calculated at the M06-2X/6-311++G(2d,3p)//B3LYP/6-31+G(d,p) level of theory (Fig. 4).

**Fig. 3:**
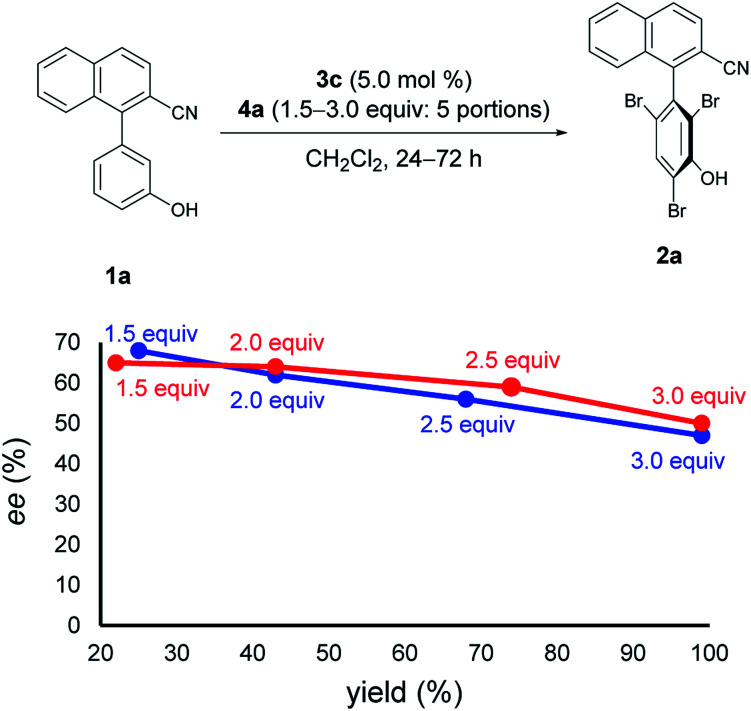
Relationships between ee and yield. Red line: reactions were run at −30 °C for 24 h with 4a added in 5 portions. Blue line: reactions were run at −40 °C for 72 h with 4a added in 5 portions. Red and blue values represent amounts of 4a used for each reaction.

**Fig. 4:**
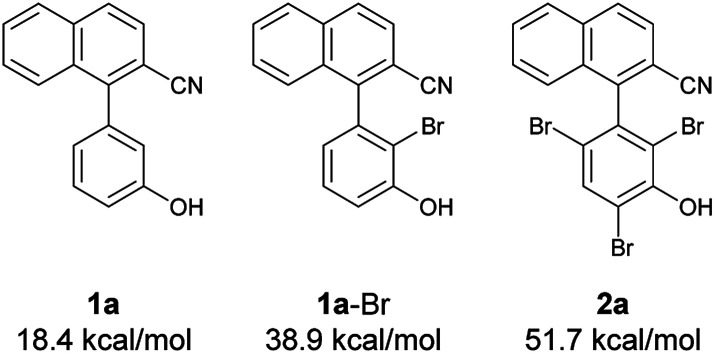
Rotational barriers of substrate, intermediate, and product calculated at the M06-2X/6-311++G(2d,3p)//B3LYP/6-31+G(d,p) level of theory.

Under the conditions of using 3c as the catalyst at −30 °C with 3 equiv. of 4a added sequentially in five portions, other substrates bearing substituted phenols were also investigated (Scheme 2).^12^ First, substrates 1b–1e bearing a substituent at the *meta*-position were investigated. While the electron-deficient substrate 1b resulted in poor enantioselectivity, 1c and 1d bearing aliphatic substituents gave improved enantioselectivities; however, substrate 1e with a methoxy group resulted in low enantioselectivity. In addition, substrates 1f–1i bearing substituents at the *para*-positions of the biaryl axis were then examined; phenol 2h bearing a methyl group resulted in higher enantioselectivities than phenols 2f and 2g bearing electron-withdrawing groups and 2i bearing a methoxy group. These results suggest that aliphatic substituents might efficiently facilitate the racemization of 1 during bromination, leading to dynamic kinetic resolution with greater enantioselectivity. Utilizing this methodology with the characteristics of dynamic kinetic resolution, the reactions of 1c and 1g were also carried out using a sub-stoichiometric amount of 4a (Scheme 3); higher enantioselectivities were accomplished albeit with lower yields.^13^ The absolute configuration of 2c was determined by X-ray crystallography (see the ESI† for details), and the configurations of all other products were assigned analogously.

**Scheme 2:**
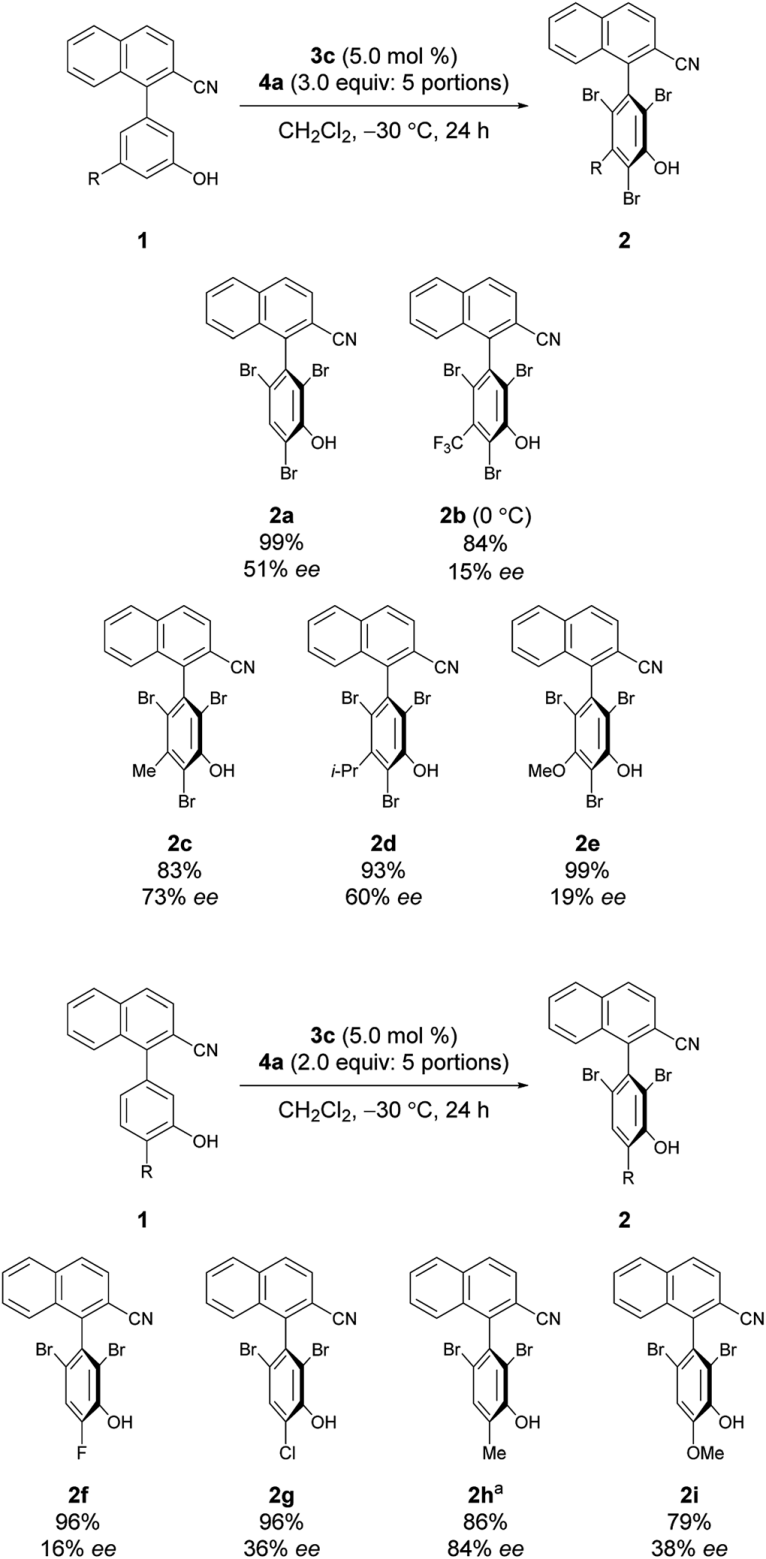
Reactions of substrates with substituted phenols. ^*a*^Reaction was run for 72 h.

**Scheme 3:**
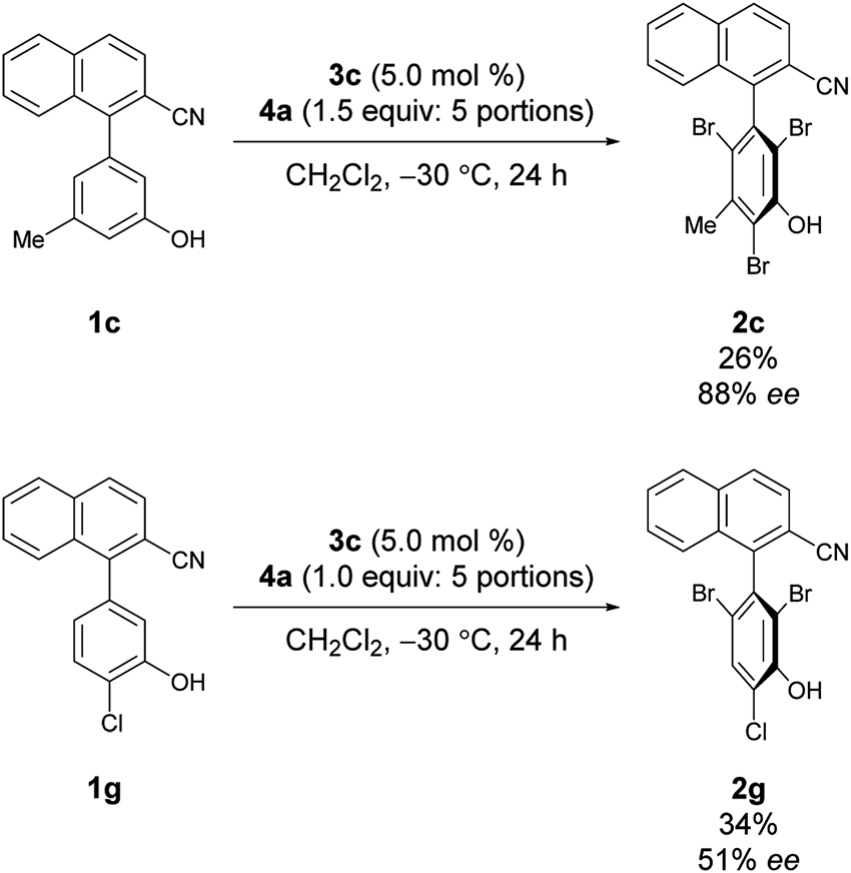
Reactions with a substoichiometric amount of 4a.

In summary, we present enantioselective bromination of axially chiral cyanoarenes bearing high intrinsic rotational barriers *via* dynamic kinetic resolution using bifunctional organocatalysts. The sequential addition of 4a in several portions at the optimized temperature was effective in improving the enantioselectivity. Although the enantioselectivities are still moderate using the current catalytic system, the guidelines for designing catalytic asymmetric syntheses of axially chiral cyanoarenes were established. Further studies on the additional optimization and application of this methodology to the construction of densely substituted axially chiral biaryls are currently underway.

## Conflicts of interest

There are no conflicts to declare.

## Supplementary Material

RA-009-C9RA05532K-s001

RA-009-C9RA05532K-s002
