# Supplementary material for: Enantioselective bromination of axially chiral cyanoarenes in the presence of bifunctional organocatalysts
Source: RSC Adv. 2019 Oct 4;9(54):31654–8. doi: 10.1039/c9ra05532k (PMC9072646; doi:10.1039/c9ra05532k)
Supplement: RA-009-C9RA05532K-s001 [file RA-009-C9RA05532K-s001.pdf]

Electronic Supplementary Material (ESI) for RSC Advances.
This journal is © The Royal Society of Chemistry 2019

Supporting Information

Enantioselective Bromination of Axially Chiral Cyanoarenes in the Presence of Bifunctional Organocatalysts

Yuuki Wada, Akira Matsumoto, Keisuke Asano,* and Seijiro Matsubara*

Department of Material Chemistry, Graduate School of Engineering, Kyoto University, Kyotodaigaku-Katsura, Nishikyo, Kyoto 615-8510, Japan

Contents

Instrumentation and Chemicals S2

Experimental Procedure S3

Table S1 S28

Scheme S1 S30

Scheme S2 S31

Scheme S3 S32

Scheme S4 S33

Scheme S5 S34

Characterization Data of Products S35

NMR Spectra (^{1}H, ^{13}C) of Products S41

HPLC Chromatogram Profiles S63

ORTEP Drawing of 5c S74

DFT Calculations of Rotational Barriers of 1a, 1a-Br, and 2a S77

Instrumentation and Chemicals

^{1}H and ^{13}C Nuclear magnetic resonance spectra were taken on a Varian UNITY INOVA 500 (^{1}H, 500 MHz; ^{13}C, 125.7 MHz) spectrometer using tetramethylsilane as an internal standard for ^{1}H NMR (δ = 0 ppm) and $CDCl_3$ as an internal standard for ^{13}C NMR (δ = 77.0 ppm). ^{1}H NMR data are reported as follows: chemical shift, multiplicity (s = singlet, d = doublet, t = triplet, q = quartet, quint = quintet, sext = sextet, sept = septet, br = broad, m = multiplet), coupling constants (Hz), integration. ^{19}F NMR spectra were measured on a Varian Mercury 200 (^{19}F, 188 MHz) spectrometer with hexafluorobenzene as an internal standard (δ = 0 ppm). Mass spectra were recorded on a Thermo Scientific Exactive (ESI, APCI) spectrometers. High performance liquid chromatography (HPLC) was performed with a SHIMADZU Prominence. Infrared (IR) spectra were determined on a SHIMADZU IR Affinity-1 spectrometer. Melting points were determined using a YANAKO MP-500D. Optical rotations were measured on a HORIBA SEPA-200. X-ray data were taken on a Rigaku XtaLAB mini diffractometer equipped with a CCD detector. TLC analyses were performed by means of Merck Kieselgel 60 F_{254} (0.25 mm) Plates. Visualization was accomplished with UV light (254 nm) and/or such as an aqueous alkaline $KMnO_4$ solution followed by heating.

Flush column chromatography was carried out using Kanto Chemical silica gel (spherical, 40–50 μm). Unless otherwise noted, commercially available reagents were used without purification.

DFT calculations were performed with Gaussian 09 packages. Geometry optimizations were carried out using the B3LYP hybrid functional with the 6-31+G(d,p) basis sets. Single-point calculations were carried out using M06-2X hybrid functional with the 6-311++G(2d,3p) basis set.

Experimental Procedure

General procedure for asymmetric synthesis of axially chiral cyanoarenes 2

In a 20 mL round-bottom flask were sequentially placed substrate **1** (0.10 mmol), quinine-derived bifunctional catalyst **3c** (2.8 mg, 0.0050 mmol), and CH_2Cl_2 (10 mL). The solution was stirred at –30 °C for 1 h. To the solution was added *N*-bromoacetamide (**4a**, 41 mg, 0.30 mmol or 28 mg, 0.20 mmol) in 5 portions after every 3 h. The mixture was stirred for 24 h or 72 h, and quenched with saturated aqueous $Na_2S_2O_3$ (5.0 mL). The aqueous phase was extracted with EtOAc (5.0 mL × 3). The combined organic layers were washed with brine, dried over Na_2SO_4, and concentrated in vacuo. Purification of the crude product by flush silica gel column chromatography using hexane/$CHCl_3$ (v/v = 1:2–1:10) or $CHCl_3$ as an eluent afforded the corresponding axially chiral cyanoarene **2**.

Racemic compounds were prepared using quinuclidine as a catalyst.

General procedure for methylation of 2a–2d, 2f, 2g, and 2i for analysis

In a 5 mL vial, to a solution of **2** in CH_2Cl_2 (0.80 mL) and MeOH (0.20 mL) was slowly added (diazomethyl)trimethylsilane (0.50 mL, 0.6 M in hexane, 0.30 mmol) at ambient temperature. After being stirred for 2 h, the mixture was passed through a short silica gel pad using CH_2Cl_2 and concentrated in vacuo. The reaction proceeded quantitatively to afford the corresponding product **5**, and the crude product was used for analysis without further purification.

General procedure for acetylation of 2e and 2h for analysis

In a 5 mL vial, to a solution of **2** and triethylamine (0.020 mL, 0.15 mmol) in CH_2Cl_2 (0.30 mL) was slowly added acetyl chloride (0.010 mL, 0.14 mmol) at ambient temperature. After the mixture was stirred for 20 h, H_2O (10 mL) was added. The aqueous phase was extracted with CH_2Cl_2 (5.0 mL × 3). The combined organic layers were washed with brine, dried over Na_2SO_4, and concentrated in vacuo. Purification of the crude product by flush silica gel column chromatography using hexane/$CHCl_3$ (v/v = 1:1) as an eluent afforded the corresponding product **6**.

Procedure for preparation of bifunctional catalyst 3c

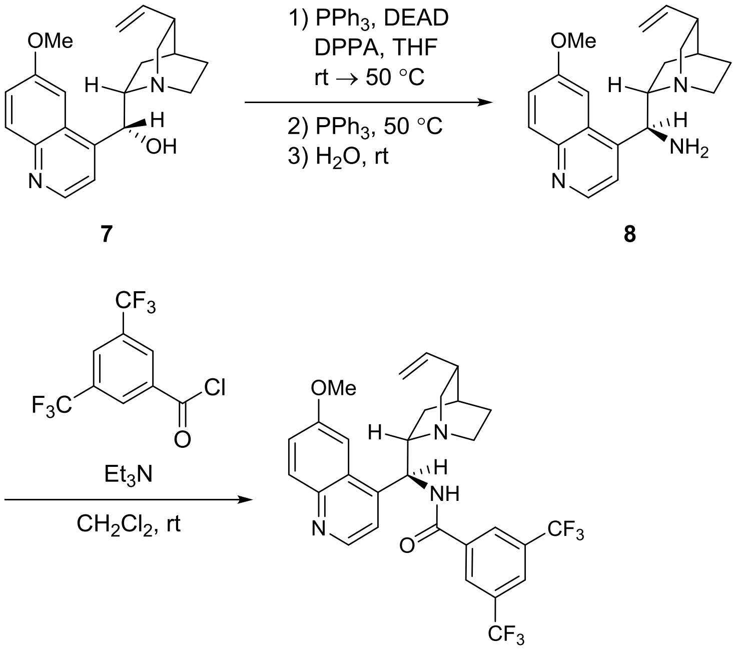

In 200 mL round-bottom flask, quinine (**7**, 2.0 g, 6.0 mmol) and triphenylphosphine (1.9 g, 7.2 mmol) were dissolved in THF (36 mL), and the solution was cooled to 0 °C. To the solution was subsequently added diethyl azodicarboxylate (3.3 mL, 7.2 mmol) and diphenyl phosphoryl azide (1.5 mL, 7.8 mmol) at 0 °C. After the mixture was stirred at ambient temperature for 24 h, it was heated to 50 °C and stirred for 10 h. Triphenylphosphine (2.0 g, 7.8 mmol) was added again, and the mixture was stirred at 50 °C for additional 15 h. After the mixture was cooled to ambient temperature, H_2O (0.60 mL) was added, and the mixture was stirred for 24 h. The solvents were removed in vacuo, and the residue was dissolved in CH_2Cl_2/1.0 M aqueous HCl (50 mL/50 mL). The aqueous phase was separated and washed with CH_2Cl_2 (25 mL × 4). It was subsequently made alkaline with aqueous NH_3, and the aqueous phase was extracted with CH_2Cl_2 (25 mL × 4). The combined organic layers were washed with brine, dried over Na_2SO_4, and concentrated in vacuo. Purification by flush silica gel column chromatography using EtOAc/CH_3OH (v/v = 8:1) then EtOAc/CH_3OH (v/v = 4:1) then $CHCl_3$/CH_3OH (v/v = 4:1) as an eluent gave (9*S*)-amino-9-deoxyquinine **8**.

Next, to the solution of **8** (0.77 g, 2.4 mmol) in THF (5.0 mL) was added triethylamine (0.41 mL, 2.4 mmol). To the resulting solution was slowly added a solution of 3,5-bis(trifluoromethyl)benzoyl chloride (0.66 mL, 4.8 mmol) in THF (5.0 mL) at ambient temperature. The mixture was stirred overnight, and the solvents were removed in

vacuo. The product was purified by flush silica gel column chromatography using $CHCl_3/CH_3OH$ (v/v = 20:1) as an eluent, and it was subsequently dissolved in CH_2Cl_2 and washed with aqueous NH_3 to give **3c**.

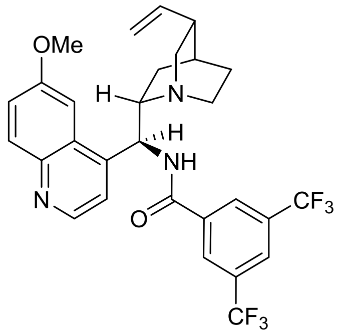

3c. White solid; 66% yield (for 2steps from quinine, 2.2 g). $[\alpha]_D^{20}$ –108.2 (*c* 0.73, CH_2Cl_2). ^1H NMR ($CDCl_3$) δ 8.76 (d, *J* = 4.5 Hz, 1H), 8.22 (s, 2H), 8.05 (d, *J* = 9.0 Hz, 1H), 7.99 (s, 1H), 7.80 (br s, 1H), 7.69 (d, *J* = 2.5 Hz, 1H), 7.42 (d, *J* = 2.5 Hz, 1H), 5.75 (ddd, *J* = 16.0, 10.5, 7.5 Hz, 1H), 5.4 (br s, 1H), 5.02 (d, *J* = 16.0 Hz, 1H), 4.99 (d, *J* = 10.5 Hz, 1H), 4.00 (s, 3H), 3.31 (m, 1H), 3.22 (m, 1H), 3.11 (m, 1H), 2.82–2.74 (m, 2H), 2.35 (m, 1H), 1.73–1.65 (m, 3H), 1.52 (m, 1H), 1.04 (m, 1H). ^{13}C NMR ($CDCl_3$) δ 164.4, 158.0, 147.5, 144.7, 141.0, 135.9, 132.0 (q, *J* = 33.9 Hz), 131.9, 128.2, 127.6, 127.5, 125.10, 125.07, 122.8 (q, *J* = 273.4 Hz), 121.6, 114.8, 101.6, 59.9, 55.9, 55.6, 51.9, 40.9, 39.4, 27.8, 27.2, 26.1. ^{19}F NMR ($CDCl_3$) δ 98.8. Mp. 111.8–112.8 °C. TLC: R_f 0.17 ($CHCl_3/CH_3OH$ = 20:1). IR (KBr): 3375, 2942, 1653, 1622, 1510, 1281, 1184, 1137, 908, 853 cm^{-1}. HRMS Calcd for $C_{29}H_{27}F_6N_3O_2Na$: $[M+Na]^+$, 586.1900. Found: *m*/*z* 586.1902.

Procedure for preparation of substrate 1a

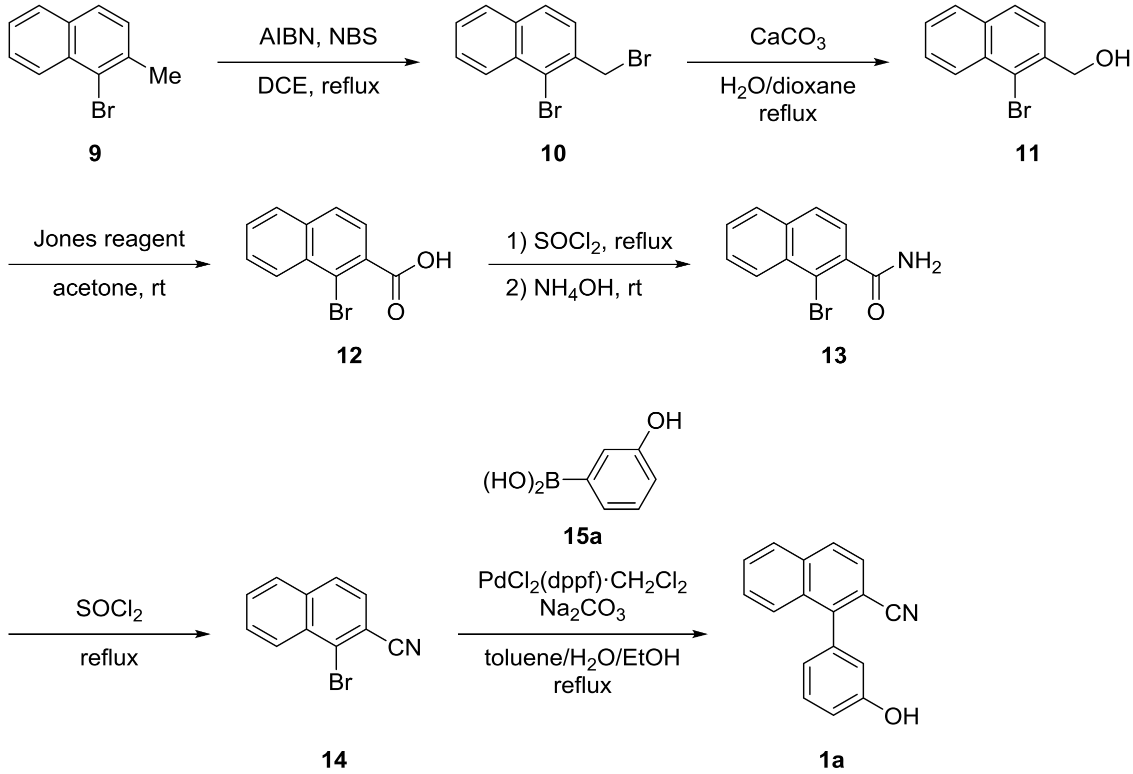

<u>Procedure for preparation of **10**</u>

In a 500 mL round-bottom flask were sequentially placed 1-bromo-2-methylnaphthalene (**9**, 7.7 mL, 50 mmol), azobisisobutyronitrile (0.82 g, 5.0 mmol), *N*-bromosuccinimide (14 g, 75 mmol) and DCE (0.30 L). The solution was refluxed in an oil bath maintained at 110 °C for 20 h. After the mixture was cooled to ambient temperature, H_2O (0.10 L) was added, and the aqueous phase was extracted with CH_2Cl_2 (50 mL × 3). The combined organic layers were washed with brine, dried over Na_2SO_4, and concentrated in vacuo to afford 1-bromo-2-(bromomethyl)naphthalene (**10**), which was used for the next step without further purification.

1-Bromo-2-(bromomethyl)naphthalene (10): CAS RN [37763-43-2].

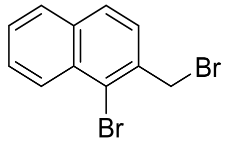

Brown solid.

^{1}H NMR ($CDCl_3$) δ 8.33 (d, *J* = 8.0 Hz, 1H), 7.81 (dd, *J* = 8.0, 1.0 Hz, 1H), 7.79 (d, *J* =

8.0 Hz, 1H), 7.62 (ddd, *J* = 8.0, 7.0, 1.0 Hz, 1H), 7.54 (ddd, *J* = 8.0, 7.0, 1.0 Hz, 1H), 7.51 (dd, *J* = 8.0, 1.0 Hz, 1H), 4.86 (s, 2H). ^{13}C NMR ($CDCl_3$) δ 134.9, 134.1, 132.4, 128.3, 128.1, 127.8, 127.7, 127.6, 127.2, 124.9, 34.8.

<u>Procedure for preparation of **11**</u>

In a 500 mL round-bottom flask were sequentially placed **10**, $CaCO_3$ (30 g, 0.30 mol), H_2O (0.10 L), and 1,4-dioxane (0.10 L). The mixture was refluxed in an oil bath maintained at 120 °C for 20 h. After being cooled to ambient temperature, the suspension was filtered. The filtrate was acidified with 1.0 M aqueous HCl, and the aqueous phase was extracted with CH_2Cl_2 (50 mL × 3). The combined organic layers were washed with brine, dried over Na_2SO_4, and concentrated in vacuo to afford (1-bromonaphthalen-2-yl)methanol (**11**), which was used for the next step without further purification.

(1-Bromonaphthalen-2-yl)methanol (11): CAS RN [76635-70-6].

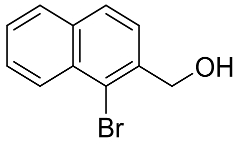

Brown solid.

^{1}H NMR ($CDCl_3$) δ 8.31 (d, *J* = 8.5 Hz, 1H), 7.84 (dd, *J* = 8.0, 1.0 Hz, 1H), 7.83 (d, *J* = 8.5 Hz, 1H), 7.64 (dd, *J* = 8.0, 1.0 Hz, 1H), 7.61 (ddd, *J* = 8.0, 7.0, 1.0 Hz, 1H), 7.53 (ddd, *J* = 8.0, 7.0, 1.0 Hz, 1H), 5.00 (d, *J* = 6.5 Hz, 2H), 2.10 (t, *J* = 6.5 Hz, 1H). ^{13}C NMR ($CDCl_3$) δ 137.7, 134.0, 132.1, 128.1, 128.0, 127.5, 126.9, 126.5, 126.0, 122.4, 65.9.

<u>Procedure for preparation of **12**</u>

In a 500 mL round-bottom flask, to a solution of **11** in acetone (0.20 L) was slowly added Jones reagent (40 mL, ca. 2.5 M, 0.10 mol) at ambient temperature. After being stirred for 24 h, the reaction was quenched with *i*-PrOH (10 mL), and concentrated in vacuo. To the resulting mixture was subsequently added H_2O, and the aqueous phase was extracted with EtOAc (50 mL × 3). The combined organic layers were washed with brine, dried over Na_2SO_4, and concentrated in vacuo to afford 1-bromo-2-naphthoic acid (**12**), which was used for the next step without further purification.

1-Bromo-2-naphthoic acid (12): CAS RN [20717-79-7].

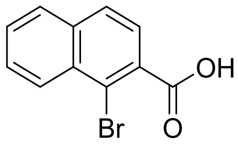

Brown solid.

^{1}H NMR ($CDCl_3$) δ 8.53 (d, *J* = 8.5 Hz, 1H), 7.89–7.87 (m, 3H), 7.68 (ddd, *J* = 8.0, 7.0, 1.0 Hz, 1H), 7.64 (ddd, *J* = 8.0, 7.0, 1.0 Hz, 1H). ^{13}C NMR ($CDCl_3$) δ 171.9, 135.6, 132.5, 129.4, 129.0, 128.6, 128.2, 127.9, 126.4, 124.1.

<u>Procedure for preparation of **13**</u>

In a 100 mL round-bottom flask were sequentially placed **12** and $SOCl_2$ (40 mL), and the mixture was refluxed in an oil bath maintained at 70 °C for 4 h. The reaction mixture was concentrated in vacuo, and 15 M aqueous NH_3 (50 mL) was slowly added. After the resulting mixture was stirred at ambient temperature for 15 h, precipitates were filtrated, washed with H_2O, and dried in vacuo to afford 1-bromo-2-naphthamide (**13**), which was used for the next step without further purification.

1-Bromo-2-naphthamide (13): CAS RN [288607-09-0].

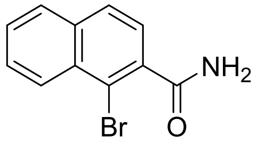

Brown solid.

^{1}H NMR ($CDCl_3$) δ 8.38 (d, *J* = 8.0 Hz, 1H), 7.87 (dd, *J* = 8.0, 1.0 Hz, 1H), 7.86 (d, *J* = 8.0 Hz, 1H), 7.67 (ddd, *J* = 8.0, 7.0, 1.0 Hz, 1H), 7.61 (ddd, *J* = 8.0, 7.0, 1.0 Hz, 1H), 7.59 (dd, *J* = 8.0, 1.0 Hz, 1H), 6.04 (br s, 1H), 5.90 (br s, 1H). ^{13}C NMR ($CDCl_3$) δ 170.3, 135.3, 134.7, 131.9, 128.4, 128.3, 128.2, 128.0, 127.8, 125.1, 119.7.

<u>Procedure for preparation of **14**</u>

In a 100 mL round-bottom flask were sequentially placed **13** and $SOCl_2$ (40 mL), and the mixture was refluxed in an oil bath maintained at 70 °C for 14 h. Precipitates were filtrated, washed with H_2O, and dried in vacuo to afford 1-bromo-2-naphthonitrile (**14**), which was used for the next step without further purification.

1-Bromo-2-naphthonitrile (14): CAS RN [20176-08-3].

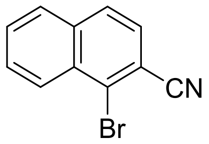

Brown solid; 91% yield (5 steps, 10.6 g).

^{1}H NMR ($CDCl_3$) δ 8.34 (d, *J* = 8.0 Hz, 1H), 7.90 (d, *J* = 8.0 Hz, 1H), 7.90 (dd, *J* = 8.0, 1.0 Hz, 1H), 7.73 (ddd, *J* = 8.0, 7.0, 1.0 Hz, 1H), 7.69 (ddd, *J* = 8.0, 7.0, 1.0 Hz, 1H), 7.60 (dd, *J* = 8.0, 1.0 Hz, 1H). ^{13}C NMR ($CDCl_3$) δ 135.4, 131.7, 129.6, 129.0, 128.6, 128.5, 128.3, 127.4, 118.1, 114.7, 113.5.

<u>Procedure for preparation of **1a**</u>

In a 100 mL round-bottom flask, **14** (1.8 g, 7.5 mmol) and (3-hydroxyphenyl)boronic acid **15a** (1.2 g, 9.0 mmol) were dissolved in toluene (42 mL) and EtOH (6.0 mL). To the mixture were sequentially added $PdCl_2(dppf)\cdot CH_2Cl_2$ (0.18 g, 0.22 mmol), Na_2CO_3 (4.0 g, 38 mmol), and H_2O (12 mL). The mixture was refluxd in an oil bath maintained at 120 °C for 38 h. After the mixture was cooled to ambient temperature, H_2O was added, and the aqueous phase was extracted with EtOAc (30 mL × 3). The combined organic layers were washed with brine, dried over Na_2SO_4, and concentrated in vacuo. Purification by flush silica gel column chromatography using hexane/EtOAc (v/v = 3:1) as an eluent gave 1-(3-hydroxyphenyl)-2-naphthonitrile (**1a**).

1-(3-Hydroxyphenyl)-2-naphthonitrile (1a).

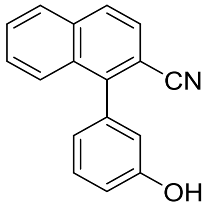

White solid; 82% yield (1.5 g).

^{1}H NMR ($CDCl_3$) δ 7.93 (d, *J* = 8.5 Hz, 1H), 7.92 (d, *J* = 8.5 Hz, 1H), 7.75 (dd, *J* = 8.5, 1.0 Hz, 1H), 7.69 (dd, *J* = 8.5, 1.0 Hz, 1H), 7.64 (ddd, *J* = 8.5, 7.5, 1.0 Hz, 1H), 7.52 (ddd, *J* = 8.5, 7.5, 1.0 Hz, 1H), 7.43 (dd, *J* = 8.0, 8.0 Hz, 1H), 7.03–6.99 (m, 2H), 6.93 (dd, *J* = 2.0, 1.0 Hz, 1H), 5.16 (br s, 1H). ^{13}C NMR ($CDCl_3$) δ 155.7, 146.1, 137.7, 134.9, 131.5, 129.9, 128.7, 128.5, 128.2, 127.6, 127.4, 126.6, 122.4, 118.9, 117.0, 116.0, 109.4. Mp. 150.0–150.6 °C. TLC: R_f 0.31 (hexane/EtOAc = 3:1). IR (KBr): 3361, 2238, 1581, 1493, 1384, 1318, 892, 874, 821 cm^{-1}. HRMS Calcd for $C_{17}H_{11}NONa$: $[M+Na]^+$,

268.0733. Found: *m*/*z* 268.0735.

General procedure for preparation of substrates 1b–1d and 1f–1h

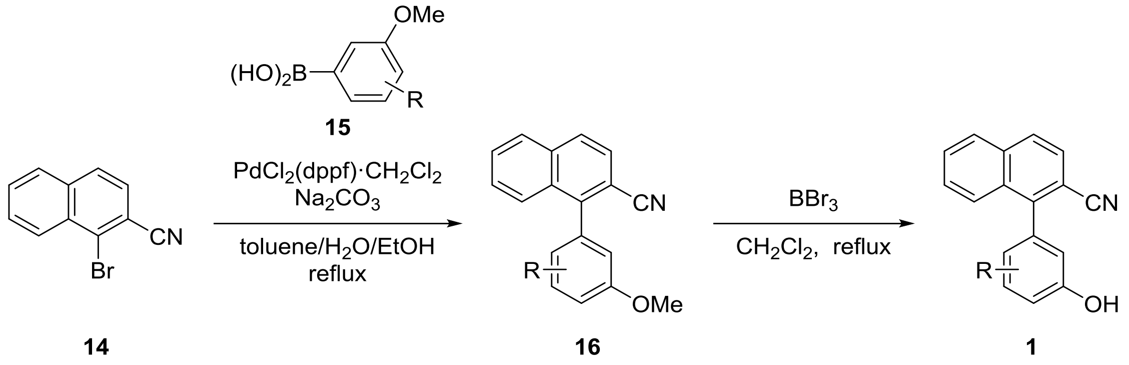

General procedure for preparation of **16**

In a 100 mL round-bottom flask, **14** (0.58 g, 2.5 mmol) and boronic acid **7** (2.5–3.0 mmol) were dissolved in toluene (18 mL) and EtOH (2.5 mL). To the mixture were sequentially added $PdCl_2$(dppf)·CH_2Cl_2 (0.061 g, 0.075 mmol), Na_2CO_3 (1.3 g, 13 mmol), and H_2O (12 mL). The mixture was refluxd in an oil bath maintained at 120 °C for 24 h. After the mixture was cooled to ambient temperature, H_2O was added, and the aqueous phase was extracted with EtOAc (20 mL × 3). The combined organic layers were washed with brine, dried over Na_2SO_4, and concentrated in vacuo. Purification by flush silica gel column chromatography using hexane/$CHCl_3$ (v/v = 3:2–1:2) as an eluent gave the corresponding product **16**.

1-(3-Methoxy-5-(trifluoromethyl)phenyl)-2-naphthonitrile (16b).

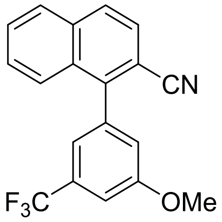

White solid; 83% yield (0.68 g).

^{1}H NMR ($CDCl_3$) δ 7.97 (d, *J* = 8.0 Hz, 1H), 7.86 (d, *J* = 8.0 Hz, 1H), 7.72 (dd, *J* = 8.5, 1.0 Hz, 1H), 7.66 (ddd, *J* = 8.5, 7.0, 1.0 Hz, 1H), 7.64 (dd, *J* = 8.5, 1.0 Hz, 1H), 7.55 (ddd, *J* = 8.5, 7.0, 1.0 Hz, 1H), 7.30 (d, *J* = 2.0 Hz, 1H), 7.29 (m, 1H), 7.17 (dd, *J* = 2.0, 2.0 Hz, 1H), 3.92 (s, 3H). ^{13}C NMR ($CDCl_3$) δ 159.9, 144.5, 138.5, 134.8, 132.2 (q, *J* = 32.6 Hz), 131.3, 129.2, 128.9, 128.4, 128.0, 126.8, 126.6, 123.7 (q, *J* = 273.4 Hz), 1

19.2, 119.0 (q, J = 3.8 Hz), 118.4, 111.3 (q, J = 3.9 Hz), 109.8, 55.7 . ^{19}F NMR ($CDCl_3$) δ 99.1. Mp. 141.8–142.5 °C. TLC: R_f 0.24 (hexane/$CHCl_3$ = 1:1). IR (KBr): 3085, 2231, 1602, 1395, 1314, 1182, 1060, 1004, 894, 868, 823 cm^{-1}. HRMS Calcd for $C_{19}H_{12}F_3NONa$: [M+Na]$^+$, 350.0763. Found: m/z 350.0771.

1-(3-Methoxy-5-methylphenyl)-2-naphthonitrile (16c).

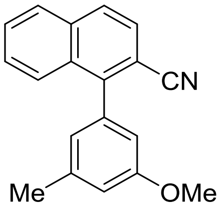

White solid; 70% yield (0.48 g).

^{1}H NMR ($CDCl_3$) δ 7.92 (d, J = 8.0 Hz, 1H), 7.91 (d, J = 8.0 Hz, 1H), 7.75 (dd, J = 8.5, 1.0 Hz, 1H), 7.68 (dd, J = 8.5, 1.0 Hz, 1H), 7.63 (ddd, J = 8.5, 7.0, 1.0 Hz, 1H), 7.51 (ddd, J = 8.5, 7.0, 1.0 Hz, 1H), 6.88 (dd, J = 1.0, 1.0 Hz, 1H), 6.84 (d, J = 1.0 Hz, 1H), 6.78 (d, J = 1.0 Hz, 1H), 3.84 (s, 3H), 2.43 (s, 3H). ^{13}C NMR ($CDCl_3$) δ 159.5, 146.5, 139.7, 137.4, 134.8, 131.6, 128.6, 128.3, 128.1, 127.5 (2C), 126.7, 123.2, 118.8, 115.2, 112.6, 109.5, 55.3, 21.7. Mp. 149.5–150.2 °C. TLC: R_f 0.18 (hexane/$CHCl_3$ = 3:2). IR (KBr): 3074, 2955, 2226, 1594, 1468, 1391, 1337, 1156, 1067, 832 cm^{-1}. HRMS Calcd for $C_{19}H_{15}NONa$: [M+Na]$^+$, 296.1046. Found: m/z 296.1053.

1-(3-Isopropyl-5-methoxyphenyl)-2-naphthonitrile (16d).

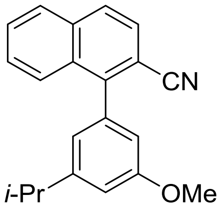

White solid; 92% yield (0.68 g).

^{1}H NMR ($CDCl_3$) δ 7.93 (d, J = 8.5 Hz, 1H), 7.91 (d, J = 8.5 Hz, 1H), 7.79 (dd, J = 8.5, 1.0 Hz, 1H), 7.69 (dd, J = 8.5, 1.0 Hz, 1H), 7.63 (ddd, J = 8.5, 7.0, 1.0 Hz, 1H), 7.51 (ddd, J = 8.5, 7.0, 1.0 Hz, 1H), 6.93 (dd, J = 1.5, 1.5 Hz, 1H), 6.91 (dd, J = 2.5, 1.5 Hz, 1H), 6.79 (dd, J = 2.5, 1.5 Hz, 1H), 3.85 (s, 3H), 2.97 (sept, J = 7.0 Hz, 1H), 1.30 (m, 6H). ^{13}C NMR ($CDCl_3$) δ 159.4, 150.7, 146.6, 137.3, 134.8, 131.5, 128.6, 128.3, 128.1, 127.43, 127.39, 126.7, 120.9, 118.8, 112.9, 112.5, 109.5, 55.2, 34.1, 23.9, 23.8. Mp. 90.8–91.5 °C. TLC: R_f 0.19 (hexane/$CHCl_3$ = 3:2). IR (KBr): 2962, 2230, 1595, 1465, 1388, 1327, 1288, 1047, 866, 817 cm^{-1}. HRMS Calcd for $C_{21}H_{19}NONa$: [M+Na]$^+$, 324.1359.

Found: *m*/*z* 324.1365.

1-(4-Fluoro-3-methoxyphenyl)-2-naphthonitrile (16f).

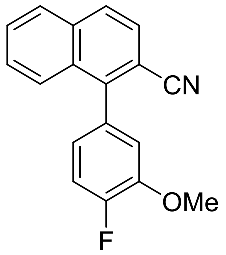

White solid; 85% yield (0.59 g).

^{1}H NMR ($CDCl_3$) δ 7.95 (d, *J* = 8.5 Hz, 1H), 7.94 (d, *J* = 8.5 Hz, 1H), 7.72 (dd, *J* = 8.5, 1.0 Hz, 1H), 7.70 (dd, *J* = 8.5, 1.0 Hz, 1H), 7.65 (ddd, *J* = 8.5, 6.5, 1.0 Hz, 1H), 7.54 (ddd, *J* = 8.5, 6.5, 1.0 Hz, 1H), 7.28 (d, *J* = 8.5 Hz, 1H), 7.04 (dd, *J* = 8.5, 2.0 Hz, 1H), 6.99 (m, 1H), 3.92 (s, 3H). ^{13}C NMR ($CDCl_3$) δ 152.6 (d, *J* = 247.5 Hz), 147.7 (d, *J* = 10.4 Hz), 145.4, 134.9, 132.6 (d, *J* = 3.8 Hz), 131.6, 128.8, 128.7, 128.3, 127.8, 127.1, 126.6, 122.6 (d, *J* = 7.7 Hz), 118.7, 116.3 (d, *J* = 19.2 Hz), 115.2, 109.9, 56.3. ^{19}F NMR ($CDCl_3$) δ 26.9. Mp. 164.5–165.5 °C. TLC: R_f 0.33 (hexane/$CHCl_3$ = 1:2). IR (KBr): 3074, 2226, 1604, 1520, 1412, 1259, 1119, 1033, 883, 833 cm^{-1}. HRMS Calcd for $C_{18}H_{12}FNOFNa$: [M+Na]$^+$, 300.0795. Found: *m*/*z* 300.0801.

1-(4-Chloro-3-methoxyphenyl)-2-naphthonitrile (16g).

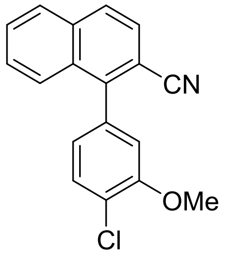

White solid; 80% yield (0.59 g).

^{1}H NMR ($CDCl_3$) δ 7.95 (d, *J* = 8.0 Hz, 1H), 7.95 (d, *J* = 8.0 Hz, 1H), 7.72 (dd, *J* = 8.5, 1.0 Hz, 1H), 7.71 (dd, *J* = 8.5, 1.0 Hz, 1H), 7.65 (ddd, *J* = 8.5, 6.5, 1.0 Hz, 1H) , 7.55 (dd, *J* = 8.0, 0.5 Hz, 1H), 7.54 (ddd, *J* = 8.5, 6.5, 1.0 Hz, 1H), 7.04 (dd, *J* = 8.0, 2.0 Hz, 1H), 6.99 (m, 1H), 3.93 (s, 3H). ^{13}C NMR ($CDCl_3$) δ 155.0, 145.2, 136.1, 134.8, 131.4, 130.4, 128.8, 128.3, 127.8, 127.0, 126.6, 123.3, 122.9, 118.6, 113.8, 109.7, 56.2. Mp. 184.5–185.5 °C. TLC: R_f 0.36 (hexane/$CHCl_3$ = 1:2). IR (KBr): 3074, 2225, 1565, 1464, 1402, 1281, 1064, 1032, 883, 832 cm^{-1}. $C_{18}H_{12}ClNONa$: [M+Na]$^+$, 316.0500. Found: *m*/*z* 316.0507.

1-(3-Methoxy-4-methylphenyl)-2-naphthonitrile (16h).

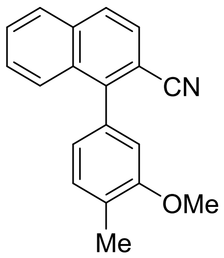

White solid; 75% yield (0.38 g).
^{1}H NMR ($CDCl_3$) δ 7.93 (d, *J* = 8.5 Hz, 1H), 7.91 (d, *J* = 8.5 Hz, 1H), 7.79 (dd, *J* = 8.5, 1.0 Hz, 1H), 7.69 (dd, *J* = 8.5, 1.0 Hz, 1H), 7.63 (ddd, *J* = 8.5, 7.0, 1.0 Hz, 1H) , 7.51 (ddd, *J* = 8.5, 7.0, 1.0 Hz, 1H), 7.30 (d, *J* = 7.5 Hz, 1H), 6.95 (dd, *J* = 7.5, 1.5 Hz, 1H), 6.89 (d, *J* = 1.5 Hz, 1H), 3.93 (s, 3H), 2.33 (s, 3H). ^{13}C NMR ($CDCl_3$) δ 157.6, 146.7, 134.87, 134.86, 131.8, 130.7, 128.6, 128.3, 128.2, 127.50, 127.46, 127.4, 126.7, 121.9, 119.0, 111.6, 109.7, 55.4, 16.2. Mp. 191.2–192.0 °C. TLC: R_f 0.16 (hexane/$CHCl_3$ = 3:2). IR (KBr): 2976, 2223, 1576, 1506, 1468, 1253, 1180, 1038, 889, 829 cm^{-1}. HRMS Calcd for $C_{19}H_{15}NONa$: $[M+Na]^+$, 296.1046. Found: *m*/*z* 296.1052.

General procedure for preparation of **1**

In a 20 mL round-bottom flask, to a solution of **6** (1.0 mmol) in CH_2Cl_2 (10 mL) was added a solution of boron tribromide (5.0 mL, ca. 1.0 M in CH_2Cl_2, 5.0 mmol) at –78 °C, and the mixture was stirred at ambient temperature for 18 h. The reaction mixture was cooled to 0 °C and quenched with H_2O (2.0 mL). After the mixture was neutralized with saturated aqueous $NaHCO_3$, the aqueous phase was extracted with EtOAc (10 mL × 3). The combined organic layers were washed with brine, dried over Na_2SO_4, and concentrated in vacuo. Purification by flush silica gel column chromatography using $CHCl_3$, CH_2Cl_2, or hexane/EtOAc (v/v = 5:1) as an eluent gave the corresponding 1-(3-hydroxyphenyl)-2-naphthonitrile **1**.

1-(3-Hydroxy-5-(trifluoromethyl)phenyl)-2-naphthonitrile (1b).

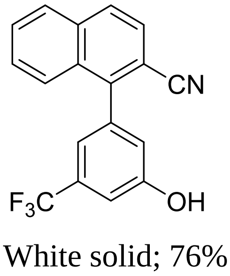

White solid; 76% yield (0.36 g).
^{1}H NMR ($CDCl_3$) δ 7.97 (d, *J* = 8.5 Hz, 1H), 7.96 (d, *J* = 8.5 Hz, 1H), 7.71 (dd, *J* = 8.5,

1.0 Hz, 1H), 7.67 (ddd, J = 8.5, 7.0, 1.0 Hz, 1H), 7.65 (dd, J = 8.5, 1.0 Hz, 1H), 7.56 (ddd, J = 8.5, 7.0, 1.0 Hz, 1H), 7.27 (m, 2H), 7.12 (dd, J = 1.5, 1.5 Hz, 1H), 5.61 (br s, 1H). ^{13}C NMR ($CDCl_3$) δ 156.5, 144.7, 138.5, 134.9, 132.4 (q, J = 32.6 Hz), 131.2, 129.2, 129.0, 128.4, 128.1, 126.9, 126.4, 123.5 (q, J = 272.4 Hz), 120.4, 118.8 (q, J = 3.9 Hz), 118.6, 113.1 (q, J = 3.9 Hz), 109.3. ^{19}F NMR ($CDCl_3$) δ 99.0. Mp. 45.6–46.6 °C. TLC: R_f 0.12 (CH_2Cl_2). IR (KBr): 3367, 2235, 1606, 1462, 1326, 1126, 868, 820 cm^{-1}. HRMS Calcd for $C_{18}H_{10}F_3NONa$: $[M+Na]^+$, 336.0607. Found: m/z 336.0611.

1-(3-Hydroxy-5-methylphenyl)-2-naphthonitrile (1c).

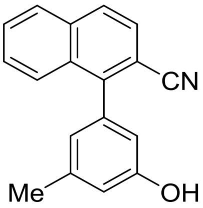

White solid; 83% yield (0.32 g).
^{1}H NMR ($CDCl_3$) δ 7.92 (d, J = 8.0 Hz, 1H), 7.91 (d, J = 8.0 Hz, 1H), 7.76 (dd, J = 8.5, 1.0 Hz, 1H), 7.68 (dd, J = 8.5, 1.0 Hz, 1H), 7.63 (ddd, J = 8.5, 7.0, 1.0 Hz, 1H), 7.51 (ddd, J = 8.5, 7.0, 1.0 Hz, 1H), 6.82 (d, J = 1.5 Hz, 1H), 6.82 (d, J = 1.5 Hz, 1H), 6.72 (dd, J = 1.5, 1.5 Hz, 1H), 5.02 (br s, 1H), 2.40 (s, 3H). ^{13}C NMR ($CDCl_3$) δ 155.6, 146.3, 140.2, 137.6, 134.9, 131.6, 128.7, 128.4, 128.2, 127.50, 127.46, 126.7, 123.3, 118.9, 116.7, 114.2, 109.4, 21.5. Mp. 197.5–198.3 °C. TLC: R_f 0.09 ($CHCl_3$). IR (KBr): 3398, 2933, 2224, 1602, 1391, 1342, 1196, 1159, 1059, 841, 821 cm^{-1}. HRMS Calcd for $C_{18}H_{13}NONa$: $[M+Na]^+$, 282.0889. Found: m/z 282.0890.

1-(3-Hydroxy-5-isopropylphenyl)-2-naphthonitrile (1d).

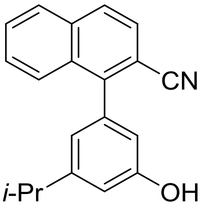

White solid; 99% yield (0.45 g).
^{1}H NMR ($CDCl_3$) δ 7.92 (d, J = 8.5 Hz, 1H), 7.91 (d, J = 8.5 Hz, 1H), 7.79 (dd, J = 8.5, 1.0 Hz, 1H), 7.68 (dd, J = 8.5, 1.0 Hz, 1H), 7.63 (ddd, J = 8.5, 7.0, 1.0 Hz, 1H), 7.52 (ddd, J = 8.5, 7.0, 1.0 Hz, 1H), 6.90 (dd, J = 1.5, 1.5 Hz, 1H), 6.88 (dd, J = 2.5, 1.5 Hz, 1H), 6.75 (dd, J = 2.5, 1.5 Hz, 1H), 5.01 (br s, 1H), 2.95 (sept, J = 6.5 Hz, 1H), 1.307 (d, J = 6.5 Hz, 3H), 1.300 (d, J = 6.5 Hz, 3H). ^{13}C NMR ($CDCl_3$) δ 155.5, 151.2, 146.4, 137.5,

134.8, 131.5, 128.7, 128.4, 128.2, 127.51, 127.46, 126.7, 121.1, 118.9, 114.4, 114.0, 109.5, 34.0, 23.9, 23.8. Mp. 187.5–188.5 °C. TLC: R_f 0.25 (hexane/EtOAc = 5:1). IR (KBr): 3345, 2962, 2236, 1597, 1395, 1344, 1298, 1181, 864, 820 cm^{-1}. HRMS Calcd for $C_{20}H_{17}NONa$: $[M+Na]^+$, 310.1202. Found: *m*/*z* 310.1203.

1-(4-Fluoro-3-hydroxyphenyl)-2-naphthonitrile (1f).

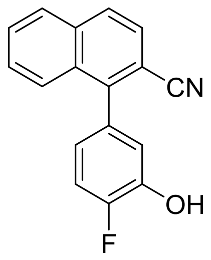

White solid; 95% yield (0.25 g).
^{1}H NMR ($CDCl_3$) δ 7.93 (d, *J* = 8.0 Hz, 1H), 7.93 (d, *J* = 8.0 Hz, 1H), 7.71 (dd, *J* = 8.5, 1.0 Hz, 1H), 7.69 (dd, *J* = 8.5, 1.0 Hz, 1H), 7.64 (ddd, *J* = 8.5, 7.0, 1.0 Hz, 1H), 7.53 (ddd, *J* = 8.5, 7.0, 1.0 Hz, 1H), 7.27 (dd, *J* = 10.0, 8.0 Hz, 1H), 7.10 (dd, *J* = 8.0, 2.0 Hz, 1H), 6.95 (ddd, *J* = 8.0, 4.5, 2.0 Hz, 1H), 5.24 (br s, 1H). ^{13}C NMR ($CDCl_3$) δ 151.3 (d, *J* = 239.7 Hz), 145.1, 143.5 (d, *J* = 16.0 Hz), 134.8, 133.2 (d, *J* = 3.9 Hz), 131.5, 128.8, 128.3, 127.7, 127.1, 126.6, 122.6 (d, *J* = 6.7 Hz), 119.22, 119.20, 118.7, 115.9 (d, *J* = 18.2 Hz), 109.8. ^{19}F NMR ($CDCl_3$) δ 22.1. Mp. 167.8–168.5 °C. TLC: R_f 0.10 ($CHCl_3$). IR (KBr): 3287, 2241, 1610, 1515, 1328, 1280, 1198, 1111, 823 cm^{-1}. HRMS Calcd for $C_{17}H_{10}FNONa$: $[M+Na]^+$, 286.0639. Found: *m*/*z* 286.0640.

1-(4-Chloro-3-hydroxyphenyl)-2-naphthonitrile (1g).

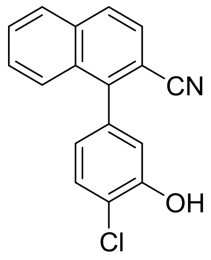

White solid; 88% yield (0.25 g).
^{1}H NMR ($CDCl_3$) δ 7.94 (d, *J* = 8.0 Hz, 1H), 7.94 (d, *J* = 8.0 Hz, 1H), 7.71 (dd, *J* = 8.5, 1.0 Hz, 1H), 7.69 (dd, *J* = 8.5, 1.0 Hz, 1H), 7.65 (ddd, *J* = 8.5, 7.0, 1.0 Hz, 1H), 7.53 (ddd, *J* = 8.5, 7.0, 1.0 Hz, 1H), 7.5 (d, *J* = 8.5 Hz, 1H), 7.12 (d, *J* = 2.0 Hz, 1H), 6.98 (d, *J* = 8.5, 2.0 Hz, 1H), 5.77 (br s, 1H). ^{13}C NMR ($CDCl_3$) δ 151.4, 144.8, 136.8, 134.8, 131.3, 129.3, 128.9, 128.8, 128.3, 127.8, 127.0, 126.6, 123.0, 120.7, 118.6, 118.0, 106.6. Mp.

150.2–150.8 °C. TLC: R_f 0.20 ($CHCl_3$). IR (KBr): 3338, 2228, 1595, 1414, 1297, 1148, 1057, 869, 826 cm^{-1}. HRMS Calcd for $C_{17}H_{10}ClNONa$: $[M+Na]^+$, 302.0343. Found: *m*/*z* 302.0346.

1-(3-Hydroxy-4-methylphenyl)-2-naphthonitrile (1h).

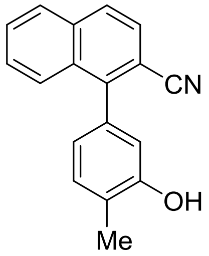

White solid; 95% yield (0.25 g).
^1H NMR ($CDCl_3$) δ 7.92 (d, *J* = 8.0 Hz, 1H), 7.90 (d, *J* = 8.0 Hz, 1H), 7.78 (dd, *J* = 8.5, 1.0 Hz, 1H), 7.68 (dd, *J* = 8.5, 1.0 Hz, 1H), 7.63 (ddd, *J* = 8.5, 7.0, 1.0 Hz, 1H), 7.51 (ddd, *J* = 8.5, 7.0, 1.0 Hz, 1H), 7.30 (d, *J* = 7.5 Hz, 1H), 6.94 (dd, *J* = 7.5, 1.5 Hz, 1H), 6.87 (d, *J* = 1.5 Hz, 1H), 5.01 (br s, 1H), 2.37 (s, 3H). ^{13}C NMR ($CDCl_3$) δ 153.8, 146.1, 135.2, 134.9, 131.6, 131.2, 128.7, 128.4, 128.2, 127.5, 127.4, 126.7, 124.7, 122.4, 119.0, 116.5, 109.5, 15.8. Mp. 149.6–150.5 °C. TLC: R_f 0.10 ($CHCl_3$). IR (KBr): 3405, 2925, 2233, 1510, 1419, 1337, 1237, 1181, 1108, 820 cm^{-1}. HRMS Calcd for $C_{18}H_{13}NONa$: $[M+Na]^+$, 282.0889. Found: *m*/*z* 282.0892.

General procedure for preparation of substrates 1e and 1i

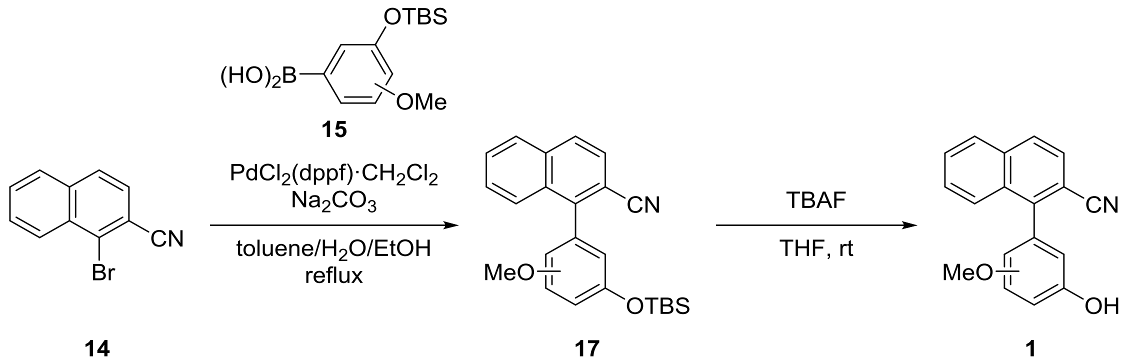

<u>General procedure for preparation of **17**</u>

In a 100 mL round-bottom flask, **14** (0.58 g, 2.5 mmol) and boronic acid **7** (2.5 mmol) were dissolved in toluene (18 mL) and EtOH (2.5 mL). To the mixture were sequentially added $PdCl_2(dppf)·CH_2Cl_2$ (0.061 g, 0.075 mmol), Na_2CO_3 (1.3 g, 13 mmol), and H_2O

(12 mL). The mixture was refluxd in an oil bath maintained at 120 °C for 24 h. After the mixture was cooled to ambient temperature, H_2O was added, and the aqueous phase was extracted with EtOAc (20 mL × 3). The combined organic layers were washed with brine, dried over Na_2SO_4, and concentrated in vacuo. Purification by flush silica gel column chromatography using hexane/$CHCl_3$ (v/v = 3:2 or 1:1) as an eluent gave the corresponding product **17**.

1-(3-((*tert*-Butyldimethylsilyl)oxy)-5-methoxyphenyl)-2-naphthonitrile (17e).

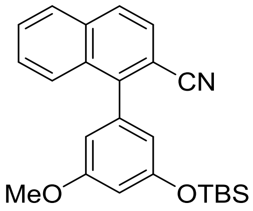

Colorless oil; 51% yield (0.69 g).
^{1}H NMR ($CDCl_3$) δ 7.92 (d, J = 8.0 Hz, 1H), 7.91 (d, J = 8.0 Hz, 1H), 7.77 (d, J = 8.5, 1.0 Hz, 1H), 7.68 (dd, J = 8.5, 1.0 Hz, 1H), 7.63 (ddd, J = 8.5, 7.0, 1.0 Hz, 1H), 7.51 (ddd, J = 8.5, 7.0, 1.0 Hz, 1H), 6.59 (dd, J = 2.5, 1.5 Hz, 1H), 6.56 (dd, J = 2.5, 2.5 Hz, 1H), 6.52 (dd, J = 2.5, 1.5 Hz, 1H), 3.82 (s, 3H), 0.99 (s, 9H), 0.24 (s, 6H). ^{13}C NMR ($CDCl_3$) δ 160.6, 156.8, 146.1, 138.1, 134.7, 131.5, 128.6, 128.4, 128.1, 127.5, 127.4, 126.6, 118.6, 114.5, 109.5, 108.8, 106.7, 55.4, 25.6, 18.1, –4.4, –4.5. TLC: R_f 0.23 (hexane/$CHCl_3$ = 3:2). IR (KBr): 2931, 2857, 2226, 1591, 1432, 1389, 1339, 1254, 1197, 1160, 1060, 985, 839 cm^{-1}. HRMS Calcd for $C_{24}H_{27}NO_2SiNa$: $[M+Na]^+$, 412.1703. Found: m/z 412.1713.

1-(3-((*tert*-Butyldimethylsilyl)oxy)-4-methoxyphenyl)-2-naphthonitrile (17i).

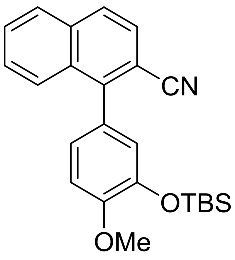

White solid; 80% yield (0.93 g).
^{1}H NMR ($CDCl_3$) δ 7.92 (d, J = 8.0 Hz, 1H), 7.89 (d, J = 8.0 Hz, 1H), 7.78 (dd, J = 8.5, 1.0 Hz, 1H), 7.67 (dd, J = 8.5, 1.0 Hz, 1H), 7.62 (ddd, J = 8.5, 7.0, 1.0 Hz, 1H), 7.51 (ddd, J = 8.5, 7.0, 1.0 Hz, 1H), 7.02 (m, 2H), 6.94 (dd, J = 2.0, 1.0 Hz, 1H), 3.91 (s, 3H), 1.00 (s, 9H), 0.20 (m, 6H). ^{13}C NMR ($CDCl_3$) δ 151.5, 146.1, 144.8, 134.9, 131.9, 128.8, 128.6, 128.2, 127.5, 127.4, 126.8, 123.6, 122.8, 119.0, 111.9, 109.8, 55.5, 25.7, 18.4, –

4.58, –4.63. Mp. 118.8–119.7 °C. TLC: R_f 0.37 (hexane/$CHCl_3$ = 1:1). IR (KBr): 2941, 2223, 1523, 1437, 1292, 1269, 1135, 1029, 951, 850 cm^{-1}. HRMS Calcd for $C_{24}H_{27}NO_2SiNa$: $[M+Na]^+$, 412.1703. Found: *m*/*z* 412.1709.

<u>General procedure for preparation of **1**</u>

In a 20 mL round-bottom flask, to a solution of **17** (1.5 mmol) in THF (3.0 mL) was added a solution of tetrabutylammonium fluoride (1.8 mL, ca. 1.0 M in THF, 1.8 mmol) at ambient temperature. After the mixture was stirred for 24 h, H_2O was added, and the aqueous phase was extracted with EtOAc (10 mL × 3). The combined organic layers were washed with brine, dried over Na_2SO_4, and concentrated in vacuo. Purification by flush silica gel column chromatography using $CHCl_3$ or hexane/$CHCl_3$ (v/v = 1:5) as an eluent gave the corresponding 1-(3-hydroxymethoxyphenyl)-2-naphthonitrile **1**.

1-(3-Hydroxy-5-methoxyphenyl)-2-naphthonitrile (1e).

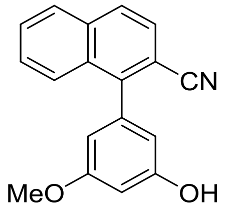

White solid; 73% yield (0.41 g).
^1H NMR ($CDCl_3$) δ 7.922 (d, *J* = 8.0 Hz, 1H), 7.916 (d, *J* = 8.0 Hz, 1H), 7.79 (dd, *J* = 8.0, 1.0 Hz, 1H), 7.68 (dd, *J* = 8.0, 1.0 Hz, 1H), 7.63 (ddd, *J* = 8.0, 7.0, 1.0 Hz, 1H), 7.52 (ddd, *J* = 8.0, 7.0, 1.0 Hz, 1H), 6.57 (dd, *J* = 2.0, 2.0 Hz, 1H), 6.56 (dd, *J* = 2.0, 1.5 Hz, 1H), 6.51 (dd, *J* = 2.0, 1.5 Hz, 1H), 5.24 (br s, 1H), 3.83 (s, 3H). ^{13}C NMR ($CDCl_3$) δ 160.8, 156.9, 146.1, 138.3, 134.8, 131.4, 128.8, 128.5, 128.1, 127.6, 127.4, 126.6, 118.8, 109.7, 109.3, 108.4, 102.0, 55.5. Mp. 153.8–154.2 °C. TLC: R_f 0.07 ($CHCl_3$). IR (KBr): 3428, 2226, 1598, 1461, 1340, 1218, 1157, 850, 822 cm^{-1}. HRMS Calcd for $C_{18}H_{13}NO_2Na$: $[M+Na]^+$, 298.0838. Found: *m*/*z* 298.0841.

1-(3-Hydroxy-4-methoxyphenyl)-2-naphthonitrile (1i).

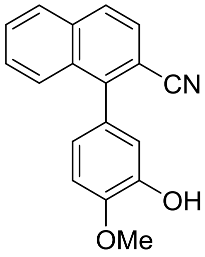

White solid; 48% yield (0.32 g).
^{1}H NMR ($CDCl_3$) δ 7.92 (d, J = 8.5 Hz, 1H), 7.89 (d, J = 8.5 Hz, 1H), 7.80 (dd, J = 8.0, 1.0 Hz, 1H), 7.67 (dd, J = 8.0, 1.0 Hz, 1H), 7.62 (ddd, J = 8.0, 6.5, 1.0 Hz, 1H), 7.51 (ddd, J = 8.0, 6.5, 1.0 Hz, 1H), 7.03 (d, J = 8.0 Hz, 1H), 7.02 (d, J = 2.0 Hz, 1H), 6.97 (dd, J = 8.0, 2.0 Hz, 1H), 5.76 (br s, 1H), 4.00 (s, 3H). ^{13}C NMR ($CDCl_3$) δ 147.0, 146.1, 145.4, 134.8, 131.7, 129.3, 128.6, 128.2, 128.1, 127.4, 126.6, 122.0, 119.0, 116.3, 110.6, 109.9, 109.7, 55.9. Mp. 180.5–181.1 °C. TLC: R_f 0.12 (hexane/$CHCl_3$ = 1:5). IR (KBr): 3382, 2956, 2233, 1517, 1441, 1388, 1281, 1131, 1027, 876, 824 cm^{-1}. HRMS Calcd for $C_{18}H_{13}NO_2Na$: $[M+Na]^+$, 298.0838. Found: m/z 298.0842.

Procedure for preparation of boronic acid 15c

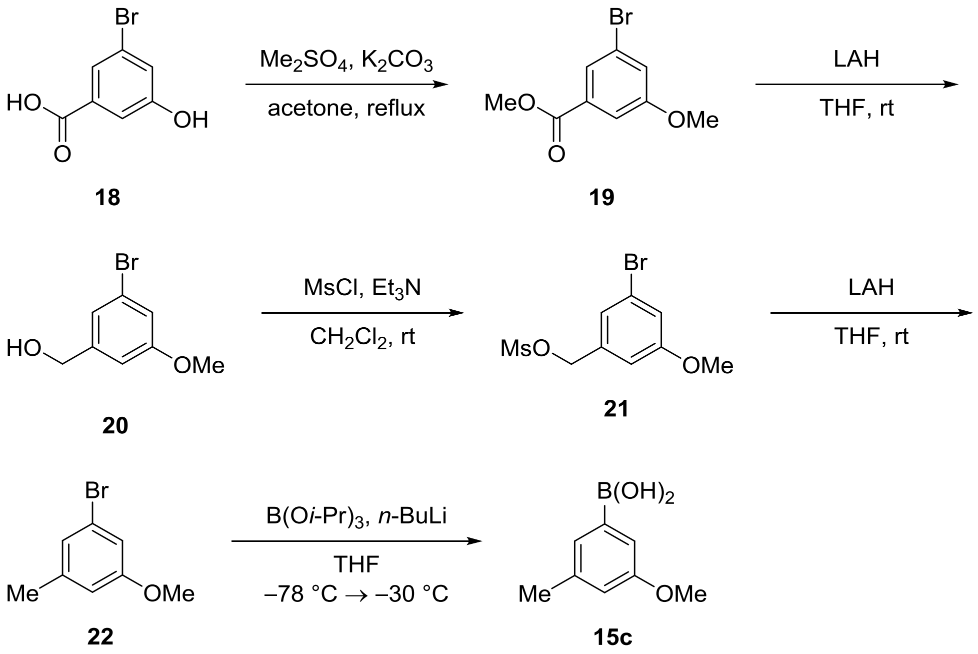

Procedure for preparation of **19**

In a 100 mL round-bottom flask were sequentially placed **18** (5.3 g, 24 mmol), K_2CO_3

(10 g, 72 mmol), acetone (40 mL), and dimethyl sulfate (5.8 mL, 60 mmol). After being refluxed in an oil bath maintained at 80 °C for 16 h, the mixture was concentrated in vacuo. After H_2O was added, the aqueous phase was extracted with CH_2Cl_2 (30 mL × 3). The combined organic layers were washed with brine, dried over Na_2SO_4, and concentrated in vacuo to quantitatively afford methyl 3-bromo-5-methoxybenzoate (**19**), which was used for the next step without further purification.

Methyl 3-bromo-5-methoxybenzoate (19): CAS RN [56709-70-7].

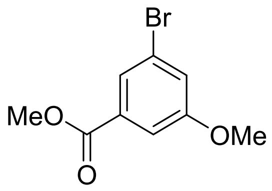

Colorless oil.
^{1}H NMR ($CDCl_3$) δ 7.74 (dd, *J* = 2.0, 1.0 Hz, 1H), 7.47 (dd, *J* = 2.0, 1.0 Hz, 1H), 7.22 (dd, *J* = 2.0, 2.0 Hz, 1H), 3.90 (s, 3H), 3.82 (s, 3H). ^{13}C NMR ($CDCl_3$) δ 165.6, 160.1, 132.6, 124.8, 122.6, 122.1, 113.4, 55.7, 52.4.

Procedure for preparation of **20**

In a 50 mL round-bottom flask, a solution of **19** in THF (10 mL) was added dropwise to a stirred suspension of lithium aluminum hydride (1.9 g, 50 mmol) in THF (15 mL) at 0 °C. The mixture was allowed to warm to ambient temperature and stirred for 5 h. To the mixture were sequentially added H_2O (1.9 mL), 15% aqueous NaOH (1.9 mL), and H_2O (5.9 mL). The resulting mixture was filtered through a Celite pad, and the Celite pad was washed with CH_2Cl_2. The combined filtrate was washed with brine, dried over Na_2SO_4, and concentrated in vacuo. Purification by flush silica gel column chromatography using $CHCl_3$ as an eluent gave (3-bromo-5-methoxyphenyl)methanol (**20**) in 73% yield from **18**.

(3-Bromo-5-methoxyphenyl)methanol (20): CAS RN [262450-64-6].

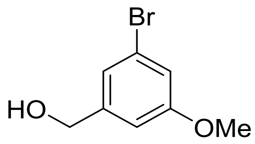

Colorless oil; 73% yield (2 steps, 3.9 g).
^{1}H NMR ($CDCl_3$) δ 7.10 (dd, *J* = 2.0, 2.0 Hz, 1H), 6.97 (dd, *J* = 2.0, 2.0 Hz, 1H), 6.85 (dd, *J* = 2.0, 2.0 Hz, 1H), 4.65 (s, 2H), 3.80 (s, 3H), 1.76 (br s, 1H). ^{13}C NMR ($CDCl_3$)

δ 160.4, 144.0, 122.8, 121.9, 116.1, 111.2, 64.3, 55.5.

Procedure for preparation of **21**

In a 50 mL round-bottom flask were sequentially placed **20** (3.9 g, 18 mmol), CH_2Cl_2 (36 mL), and Et_3N (5.0 mL, 36 mmol). Subsequently, methanesulfonyl chloride (1.5 mL, 20 mmol) was added, and the mixture was stirred at ambient temperature for 2 h. The reaction was quenched with saturated aqueous NH_4Cl (10 mL), and the aqueous phase was extracted with CH_2Cl_2 (10 mL × 3). The combined organic layers were washed with brine, dried over Na_2SO_4, and concentrated in vacuo to afford 3-bromo-5-methoxybenzyl methanesulfonate (**21**), which was used for the next step without further purification. The characterization data were difficult to collect because it was of low purity.

Procedure for preparation of **22**

In a 50 mL round-bottom flask, a solution of **21** in THF (4.0 mL) was added dropwise to a stirred suspension of lithium aluminum hydride (0.76 g, 20 mmol) in THF (6.0 mL) at 0 °C. The mixture was allowed to warm to ambient temperature and stirred for 2 h. To the mixture were sequentially added H_2O (0.80 mL), 15% aqueous NaOH (0.80 mL), and H_2O (2.2 mL). The resulting mixture was filtered through a Celite pad, and the Celite pad was washed with CH_2Cl_2. The combined filtrate was washed with brine, dried over Na_2SO_4, and concentrated in vacuo to afford 1-bromo-3-methoxy-5-methylbenzene (**22**), which was used for the next step without further purification.

1-Bromo-3-methoxy-5-methylbenzene (22): CAS RN [29578-83-4].

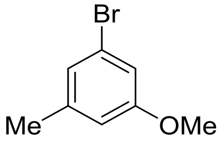

Colorless oil; 61% yield (2 steps, 2.2 g).

^{1}H NMR ($CDCl_3$) δ 6.93 (s, 1H), 6.87 (s, 1H), 6.65 (s, 1H), 3.77 (s, 3H), 2.30 (s, 3H).

^{13}C NMR ($CDCl_3$) δ 160.1, 141.0, 124.5, 122.4, 114.0, 113.9, 55.3, 21.3.

Procedure for preparation of **15c**

In a 50 mL round-bottom flask, to a solution of **22** (1.0 g, 5.0 mmol) in THF (20 mL) was added *n*-butyllithium (3.8 mL, ca. 1.6 M in *n*-hexane, 6.0 mmol) at –78 °C, and the resulting mixture was stirred at the same temperature for 45 min. Subsequently, a solution of triisopropyl borate (1.7 mL, 7.5 mmol) was added, and the mixture was stirred

at –30 °C for 75 min. The reaction was quenched with 2.5 M aqueous HCl (10 mL), and the aqueous phase was extracted with EtOAc (10 mL × 3). The combined organic layers were washed with brine, dried over Na_2SO_4, and concentrated in vacuo. Purification by flush silica gel column chromatography using hexane/EtOAc (v/v = 3:1) as an eluent gave (3-methoxy-5-methylphenyl)boronic acid (**15c**). The characterization data were difficult to collect because it was of low purity.

Procedure for preparation of boronic acid 15d

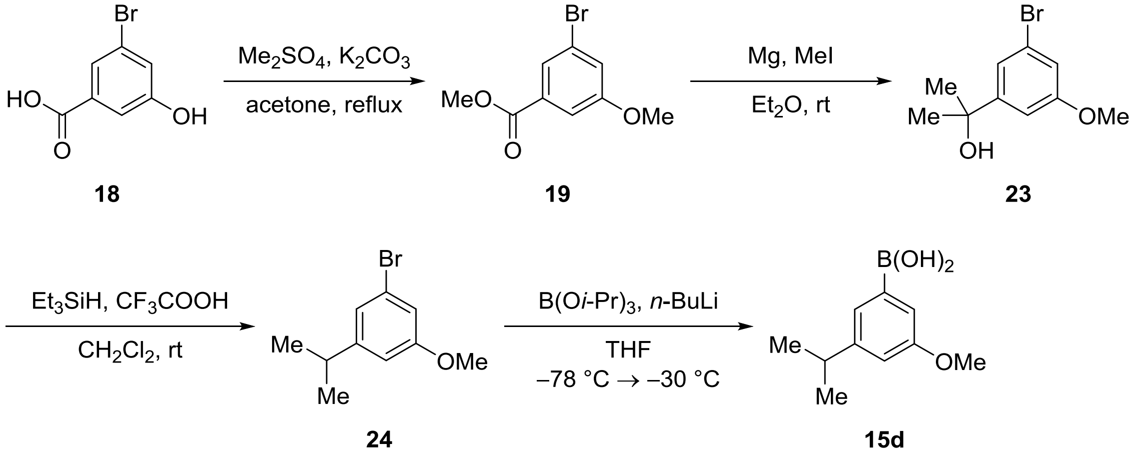

<u>Procedure for preparation of **19**</u>

In a 30 mL round-bottom flask were sequentially placed **18** (3.1 g, 14 mmol), K_2CO_3 (5.7 g, 42 mmol), acetone (21 mL), and dimethyl sulfate (3.4 mL, 35 mmol). After being refluxed in an oil bath maintained at 80 °C for 24 h, the mixture was concentrated in vacuo. After H_2O was added, the aqueous phase was extracted with CH_2Cl_2 (20 mL × 3). The combined organic layers were washed with brine, dried over Na_2SO_4, and concentrated in vacuo to quantitatively afford methyl 3-bromo-5-methoxybenzoate (**19**), which was used for the next step without further purification. The characterization results of **19** are as described above.

<u>Procedure for preparation of **23**</u>

In a 50 mL round-bottom flask, a solution of iodomethane (2.6 mL, 42 mmol) in Et_2O (11 mL) was added dropwise to a stirred suspension of magnesium turnings (1.0 g, 42 mmol) in Et_2O (10 mL) at 0 °C. The mixture was allowed to warm to ambient temperature and stirred for 1 h. To the resulting mixture was added a solution of **19** (3.3 g, 13 mmol) in Et_2O (5.0 mL), and the mixture was stirred at ambient temperature for 15

h. The reaction was quenched with saturated aqueous NH_4Cl (50 mL), and the aqueous phase was extracted with Et_2O (30 mL × 3). The combined organic layers were washed with brine, dried over Na_2SO_4, and concentrated in vacuo to quantitatively afford 2-(3-bromo-5-methoxyphenyl)propan-2-ol (**23**), which was used for the next step without further purification.

2-(3-Bromo-5-methoxyphenyl)propan-2-ol (23): CAS RN [1204344-31-9].

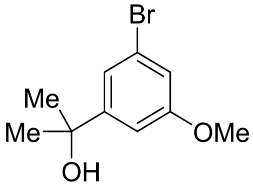

Colorless oil; 96% yield (3.3 g).
^{1}H NMR ($CDCl_3$) δ 7.19 (dd, *J* = 3.0, 2.0 Hz, 1H), 6.97 (dd, *J* = 3.5, 2.0 Hz, 1H), 6.92 (dd, *J* = 3.5, 3.0 Hz, 1H), 3.79 (s, 3H), 1.88 (br s, 1H), 1.54 (s, 3H), 1.53 (s, 3H). ^{13}C NMR ($CDCl_3$) δ 160.2, 152.5, 122.6, 120.2, 114.9, 109.8, 72.3, 55.5, 31.61, 31.57.

Procedure for preparation of **24**

In a 200 mL round-bottom flask were sequentially placed **23** (3.3 g, 13 mmol), CH_2Cl_2 (90 mL), and trifluoroacetic acid (10 mL, 0.14 mol). Subsequently, triethylsilane (6.7 mL, 42 mmol) was added, and the mixture was stirred at ambient temperature for 19 h. It was made alkaline with aqueous NH_3, and the aqueous phase was extracted with CH_2Cl_2 (30 mL × 3). The combined organic layers were washed with brine, dried over Na_2SO_4, and concentrated in vacuo. Purification by flush silica gel column chromatography using hexane as an eluent gave 1-bromo-3-isopropyl-5-methoxybenzene (**24**) in 41% yield from **18**.

1-Bromo-3-isopropyl-5-methoxybenzene (24): CAS RN [1204344-29-5].

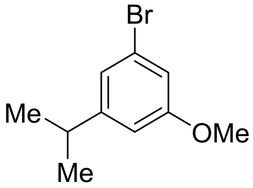

Colorless oil; 42% yield (1.3 g).
^{1}H NMR ($CDCl_3$) δ 6.96 (dd, *J* = 2.0, 1.5 Hz, 1H), 6.87 (dd, *J* = 2.0, 2.0 Hz, 1H), 6.69 (dd, *J* = 2.0, 1.5 Hz, 1H), 3.78 (s, 3H), 2.83 (sept, *J* = 7.0 Hz, 1H), 1.22 (d, *J* = 7.0 Hz,

6H). ^{13}C NMR ($CDCl_3$) δ 160.2, 152.1, 122.5, 122.1, 114.0, 111.7, 55.4, 34.1, 23.7.

Procedure for preparation of **15d**

In a 50 mL round-bottom flask, to a solution of **24** (1.1 g, 5.0 mmol) in THF (20 mL) was added *n*-butyllithium (3.8 mL, ca. 1.6 M in *n*-hexane, 6.0 mmol) at –78 °C, and the resulting mixture was stirred at the same temperature for 45 min. Subsequently, a solution of triisopropyl borate (1.7 mL, 7.5 mmol) was added, and the mixture was stirred at –30 °C for 75 min. The reaction was quenched with 2.5 M aqueous HCl (10 mL), and the aqueous phase was extracted with EtOAc (10 mL × 3). The combined organic layers were washed with brine, dried over Na_2SO_4, and concentrated in vacuo. Purification by flush silica gel column chromatography using hexane/EtOAc (v/v = 3:1) as an eluent gave (3-isopropyl-5-methoxyphenyl)boronic acid (**15d**). The characterization data were difficult to collect because it was of low purity.

Procedure for preparation of boronic acid 15e

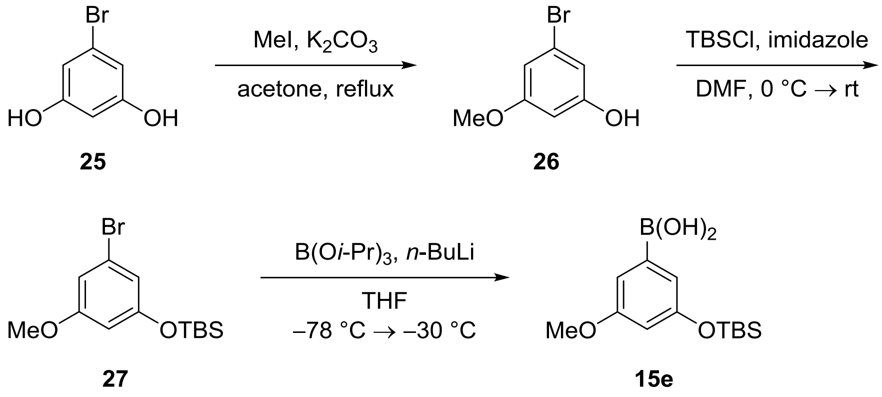

Procedure for preparation of **26**

In a 200 mL round-bottom flask were sequentially added **25** (5.2 g, 28 mmol), K_2CO_3 (5.7 g, 41 mmol), acetone (80 mL), and iodomethane (1.8 mL, 29 mmol). After being refluxed in an oil bath maintained at 80 °C for 18 h, the mixture was concentrated in vacuo. After 1.0 M aqueous HCl was added until the pH of the mixture became 4, the aqueous phase was extracted with CH_2Cl_2 (30 mL × 3). The combined organic layers were washed with brine, dried over Na_2SO_4, and concentrated in vacuo. Purification by flush silica gel column chromatography using hexane/EtOAc (v/v/ = 5:1) as an eluent gave 3-bromo-5-methoxyphenol (**26**) in 36% yield.

3-Bromo-5-methoxyphenol (26): CAS RN [855400-66-7].

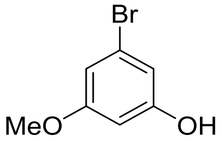

White solid; 36% yield (2.0 g).

^{1}H NMR ($CDCl_3$) δ 6.65 (dd, *J* = 2.0, 2.0 Hz, 1H), 6.61 (dd, *J* = 2.0, 2.0 Hz, 1H), 6.30 (dd, *J* = 2.0, 2.0 Hz, 1H), 5.01 (br s, 1H), 3.76 (s, 3H). ^{13}C NMR ($CDCl_3$) δ 161.3, 157.1, 122.9, 111.4, 109.9, 100.8, 55.5.

Procedure for preparation of **27**

In a 100 mL round-bottom flask were sequentially placed **26** (2.0 g, 10 mmol), DMF (60 mL), imidazole (2.7 g, 40 mmol), and *tert*-butyldimethylchlorosilane (3.1 g, 20 mmol), and the mixture was stirred at ambient temperature for 36 h. The reaction was quenched with H_2O (30 mL), and the aqueous phase was extracted with EtOAc (30 mL × 3). The combined organic layers were washed with brine, dried over Na_2SO_4, and concentrated in vacuo to quantitatively afford (3-bromo-5-methoxyphenoxy)(*tert*-butyl)dimethylsilane (**27**), which was used for the next step without further purification.

(3-Bromo-5-methoxyphenoxy)(*tert*-butyl)dimethylsilane (27): CAS RN [1839511-43-1].

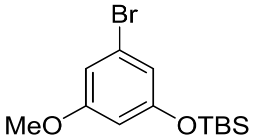

Colorless oil; 99% yield (3.2 g).

^{1}H NMR ($CDCl_3$) δ 6.68 (dd, *J* = 2.0, 2.0 Hz, 1H), 6.62 (dd, *J* = 2.0, 2.0 Hz, 1H), 6.32 (dd, *J* = 2.0, 2.0 Hz, 1H), 3.75 (s, 3H), 0.98 (s, 9H), 0.20 (s, 6H). ^{13}C NMR ($CDCl_3$) δ 161.0, 157.3, 122.5, 116.1, 110.3, 105.5, 55.4, 25.6, 18.1, –4.5.

Procedure for preparation of **15e**

In a 50 mL round-bottom flask, to a solution of **27** (1.6 g, 5.0 mmol) in THF (20 mL) was added *n*-butyllithium (3.8 mL, ca. 1.6 M in *n*-hexane, 6.0 mmol) at –78 °C, and the resulting mixture was stirred at the same temperature for 45 min. Subsequently, a solution of triisopropyl borate (1.7 mL, 7.5 mmol) was added, and the mixture was stirred at –30 °C for 75 min. The reaction was quenched with 2.5 M aqueous HCl (10 mL), and the aqueous phase was extracted with EtOAc (10 mL × 3). The combined organic layers were washed with brine, dried over Na_2SO_4, and concentrated in vacuo.

Purification by flush silica gel column chromatography using hexane/EtOAc (v/v = 3:1) as an eluent gave (3-((*tert*-butyldimethylsilyl)oxy)-5-methoxyphenyl)boronic acid (**15e**). The characterization data were difficult to collect because it was of low purity.

Procedure for preparation of boronic acid 15i

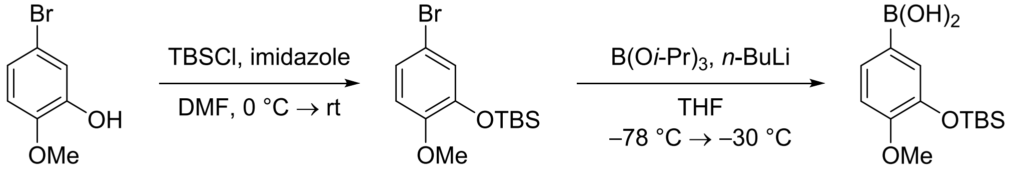

28 **29** **15i**

<u>Procedure for preparation of **29**</u>

In a 100 mL round-bottom flask were sequentially placed **28** (2.0 g, 10 mmol), DMF (60 mL), imidazole (2.7 g, 40 mmol) and *tert*-butyldimethylchlorosilane (3.1 g, 20 mmol), and the mixture was stirred at ambient temperature for 18 h. The reaction was quenched with H_2O (30 mL), and the aqueous phase was extracted with EtOAc (30 mL × 3). The combined organic layers were washed with brine, dried over Na_2SO_4, and concentrated in vacuo to quantitatively afford (5-bromo-2-methoxyphenoxy)(*tert*-butyl)dimethylsilane (**29**), which was used for the next step without further purification.

(5-Bromo-2-methoxyphenoxy)(*tert*-butyl)dimethylsilane (29): CAS RN [177329-71-4].

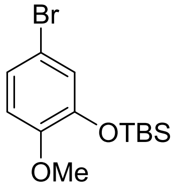

Colorless oil; 99% yield (3.2 g).

^{1}H NMR ($CDCl_3$) δ 7.02 (dd, J = 8.0, 2.0 Hz, 1H), 6.97 (d, J = 2.0 Hz, 1H), 6.71 (d, J = 8.0 Hz, 1H), 3.78 (s, 3H), 0.98 (s, 9H), 0.15 (s, 6H). ^{13}C NMR ($CDCl_3$) δ 150.4, 145.9, 124.4, 124.0, 113.1, 112.2, 55.5, 25.6, 18.4, –4.7.

<u>Procedure for preparation of **15i**</u>

In a 50 mL round-bottom flask, to a solution of **29** (1.6 g, 5.0 mmol) in THF (20 mL) was added *n*-butyllithium (3.8 mL, ca. 1.6 M in *n*-hexane, 6.0 mmol) at –78 °C, and the

resulting mixture was stirred at the same temperature for 45 min. Subsequently, a solution of triisopropyl borate (1.7 mL, 7.5 mmol) was added, and the mixture was stirred at –30 °C for 75 min. The reaction was quenched with 2.5 M aqueous HCl (10 mL), and the aqueous phase was extracted with EtOAc (10 mL × 3). The combined organic layers were washed with brine, dried over Na_2SO_4, and concentrated in vacuo. Purification by flush silica gel column chromatography using hexane/EtOAc (v/v = 3:1) as an eluent gave (3-((*tert*-butyldimethylsilyl)oxy)-4-methoxyphenyl)boronic acid (**15i**). The characterization data were difficult to collect because it was of low purity.

Table S1. Screening of Catalysts[a]

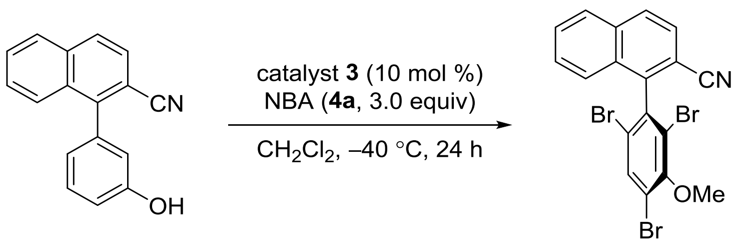

1a **2a**

entry	catalyst	yield (%)[b]	ee (%)
1	**3a**	82	20
2	**3c**	83	41
3	**3g**	68	47
4	**3h**	30	13
5	**3i**	29	47
6	**3j**	71	39
7	**3k**	75	25
8	**3l**	21	50
9	**3m**	95	3
10	**3n**	35	12
11	**3o**	44	15
12	**3p**	<5	—
13	**3q**	<5	—

[a] Reactions were run using **1a** (0.10 mmol), the catalyst (0.010 mmol), and **4a** (0.30 mmol) in CH_2Cl_2 (10 mL) with **4a** added in 1 portion. [b] Isolated yields.

(Table S1)

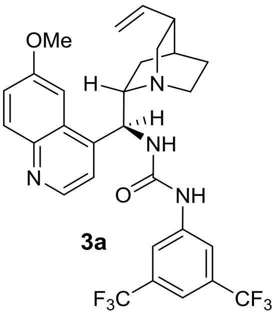

3a

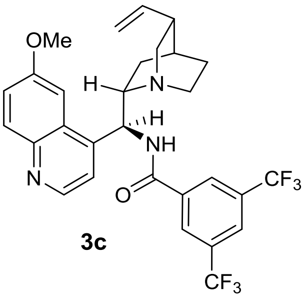

3c

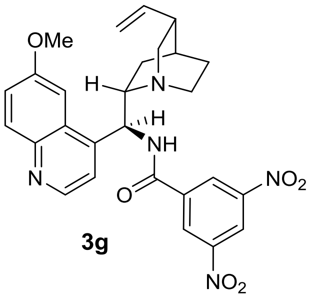

3g

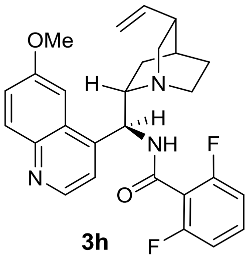

3h

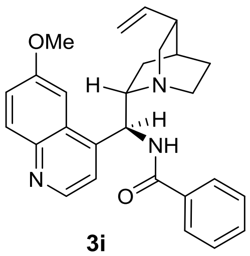

3i

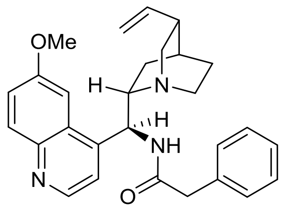

3j

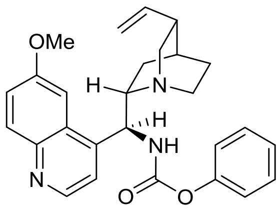

3k

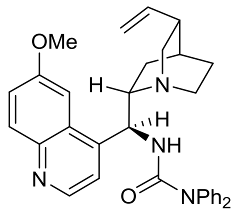

3l

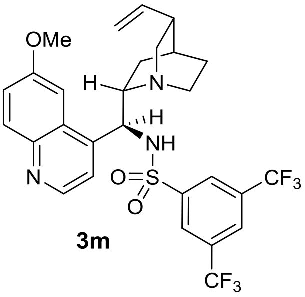

3m

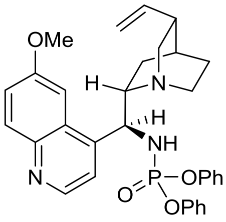

3n

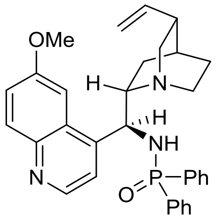

3o

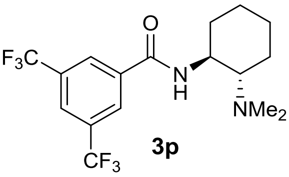

3p

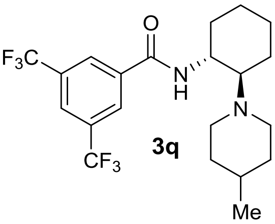

3q

Scheme S1. Reaction of **1a** with 1.5 Equiv of **4a**.

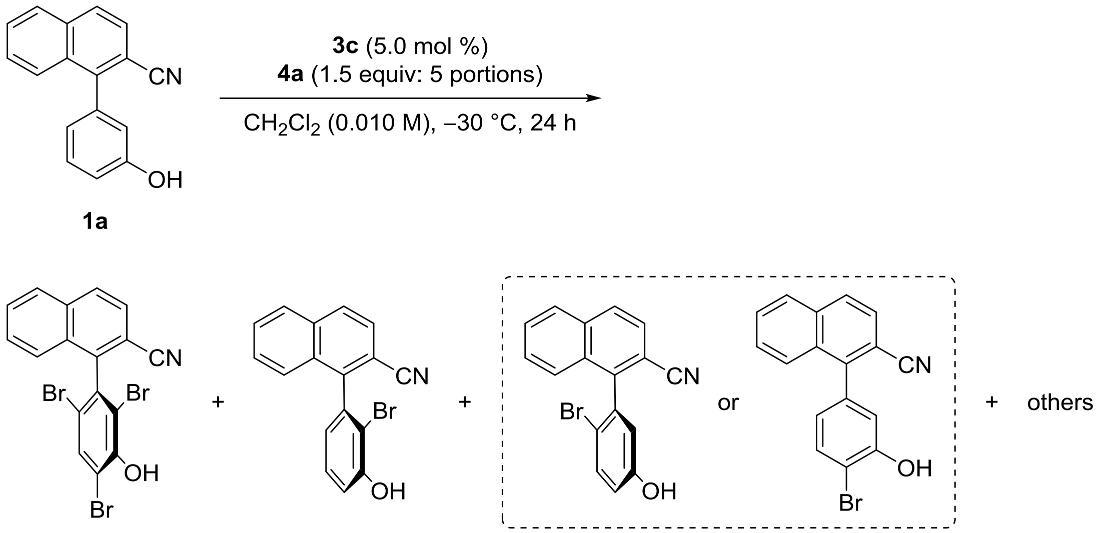

2a
22%
65% *ee*

1a-Br
75% *ee*

1a-Br'

(**1a**-Br:**1a**-Br' = 1:0.7)

A reaction of **1a** using 1.5 equiv of **4a** was carried out; a mixture of *ortho*-monobrominated products **1a**-Br and **1a**-Br' were obtained along with **2a**. These results indicate that **1a**-Br and **1a**-Br' would be the reaction intermediates; the low regioselectivity of the first bromination might be one of the reasons for the moderate enantioselectivity. Indeed, the *ortho*-monobrominated product **1a**-Br was obtained with 75% *ee*; it shows that the bromination at one of the *ortho*-positions introduces a rotational barrier high enough to set the chiral axis, which is consistent with the rotational barriers calculated at the M06-2X/6-311++G(2d,3p)//B3LYP/6-31+G(d,p) level of theory (Figure 4).

Scheme S2. Control Experiments

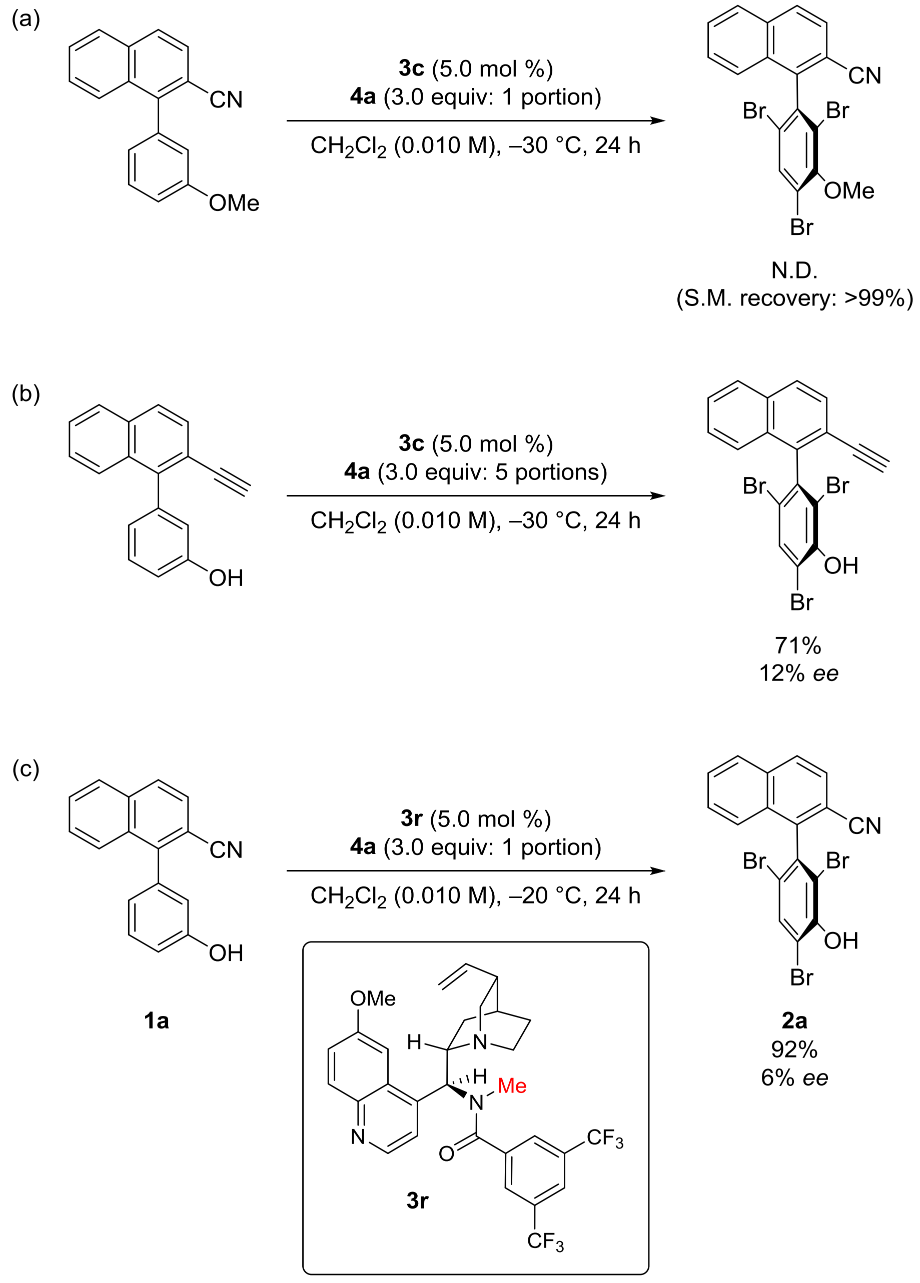

Scheme S3. Reaction of **1h** with 0.5 Equiv of **4a**.

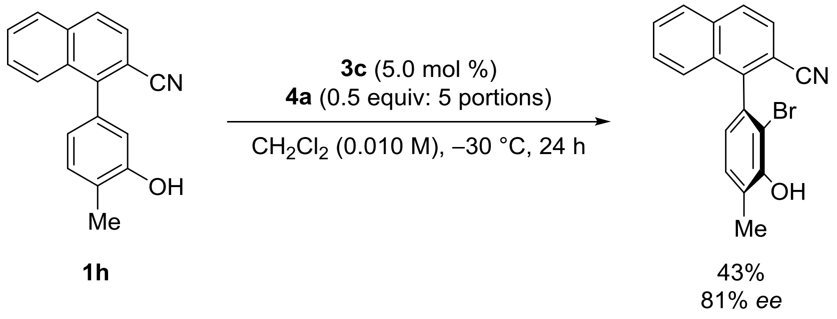

Scheme S4. Reaction of **1a**-Br with 1.0 Equiv of **4a**.

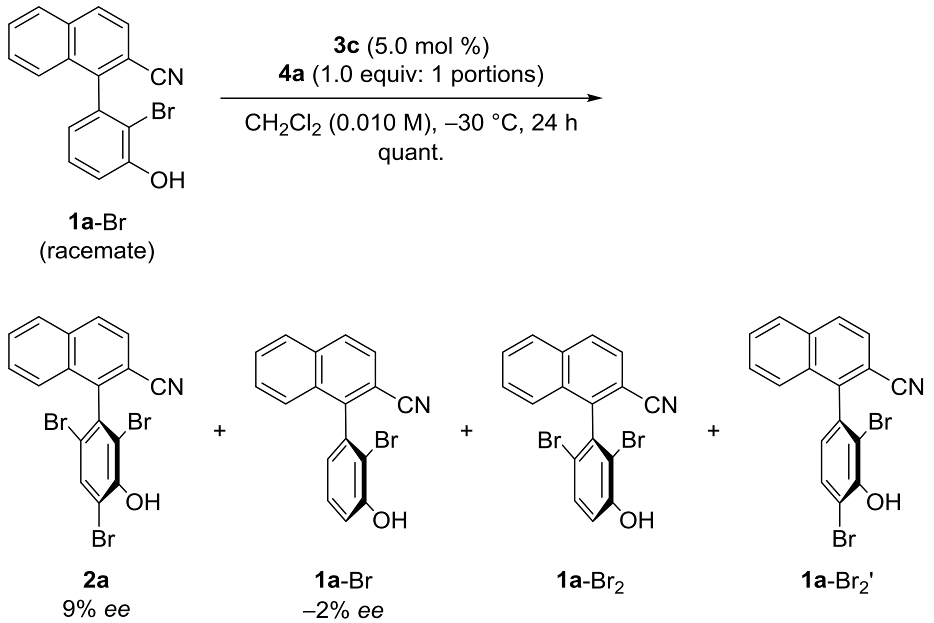

(**2a**:**1a**-Br:**1a**-Br_2:**1a**-Br_2' = 1.3:1:0.2:0.2)

Scheme S5. Reaction of Deuterated Substrate **1a**-*d*.

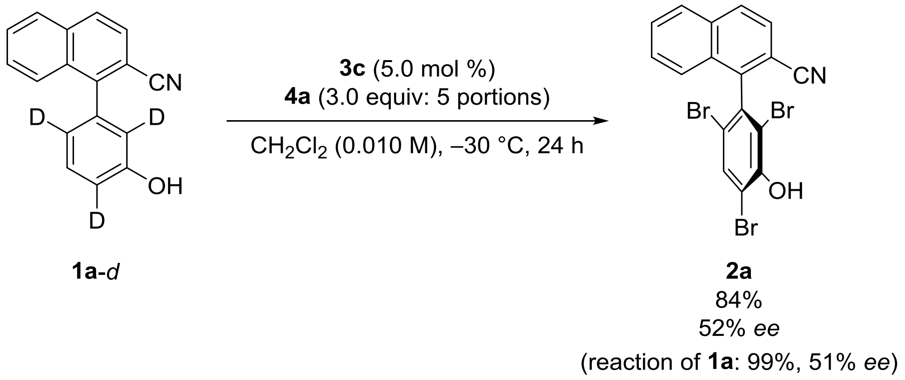

Characterization Data of Products

1-(2,4,6-Tribromo-3-methoxyphenyl)-2-naphthonitrile (5a).

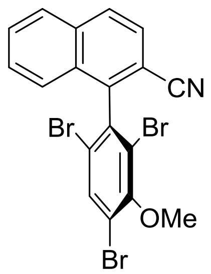

Yield of **2a**: 99% (47.7 mg), 51% *ee*, white solid. $[\alpha]_D^{20}$ –5.5 (*c* 0.93, CH_2Cl_2). ^{1}H NMR ($CDCl_3$) δ 8.03 (d, *J* = 8.5 Hz, 1H), 7.99 (s, 1H), 7.98 (d, *J* = 8.5 Hz, 1H), 7.75 (dd, *J* = 8.5, 1.0 Hz, 1H), 7.67 (ddd, *J* = 8.5, 7.0, 1.0 Hz, 1H), 7.56 (ddd, *J* = 8.5, 7.0, 1.0 Hz, 1H), 7.37 (dd, *J* = 8.5, 1.0 Hz, 1H), 3.97 (s, 3H). ^{13}C NMR ($CDCl_3$) δ 154.6, 144.0, 138.7, 136.0, 134.8, 130.1, 129.8, 129.1, 128.6, 128.4, 126.5, 125.5, 121.2, 119.5, 119.3, 117.4, 110.2, 60.9. Mp. 130.5–131.0 °C. TLC: R_f 0.33 (hexane/$CHCl_3$ = 3:2). IR (KBr): 2941, 2232, 1450, 1382, 1344, 1050, 982, 829 cm^{-1}. HRMS Calcd for $C_{18}H_{10}Br_3NONa$: $[M+Na]^+$, 517.8184. Found: *m*/*z* 517.8184. HPLC (Daicel Chiralpak IB, hexane/*i*-PrOH = 98.5/1.5, flow rate = 0.5 mL/min, λ = 254 nm, 40 °C): t_{minor} = 16.4 min, t_{major} = 18.2 min.

1-(2,4,6-Tribromo-3-methoxy-5-(trifluoromethyl)phenyl)-2-naphthonitrile (5b).

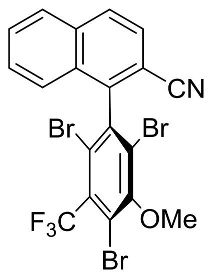

Yield of **2b**: 84% (46.2 mg), 15% *ee*, white solid. $[\alpha]_D^{20}$ –2.3 (*c* 0.22, CH_2Cl_2). ^{1}H NMR ($CDCl_3$) δ 8.05 (d, *J* = 8.0 Hz, 1H), 8.00 (d, *J* = 8.0 Hz, 1H), 7.76 (dd, *J* = 8.0, 1.0 Hz, 1H), 7.69 (ddd, *J* = 8.0, 7.0, 1.0 Hz, 1H), 7.58 (ddd, *J* = 8.0, 7.0, 1.0 Hz, 1H), 7.32 (dd, *J* = 8.0, 1.0 Hz, 1H), 3.99 (s, 3H). ^{13}C NMR ($CDCl_3$) δ 155.8, 144.7, 141.5, 134.8, 130.7 (q, *J* = 30.7 Hz), 130.0, 129.8, 129.3, 128.8, 128.7, 126.5, 125.1, 124.8, 121.9 (q, *J* = 277.2 Hz), 120.4, 118.6, 117.2, 110.1, 60.9. ^{19}F NMR ($CDCl_3$) δ 106.5. Mp. 156.0–156.6 °C. TLC: R_f 0.25 (hexane/$CHCl_3$ = 2:1). IR (KBr): 2941, 2227, 1543, 1356, 1309, 1137, 1063, 991, 823 cm^{-1}. HRMS Calcd for $C_{19}H_9Br_3F_3NONa$: $[M+Na]^+$, 585.8058. Found: *m*/*z* 585.8064. HPLC (Daicel Chiralpak IB, hexane/*i*-PrOH = 98.5/1.5, flow rate = 0.5 mL/min, λ = 254 nm, 40 °C): t_{minor} = 16.6 min, t_{major} = 15.2 min.

(*S*)-1-(2,4,6-Tribromo-3-methoxy-5-methylphenyl)-2-naphthonitrile (5c).

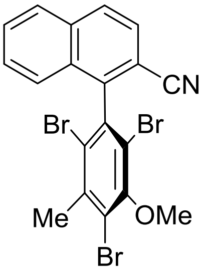

Yield of **2c**: 83% (41.2 mg), 73% *ee*, white solid. $[\alpha]_D^{20}$ –0.5 (*c* 1.03, CH_2Cl_2). ^{1}H NMR ($CDCl_3$) δ 8.01 (d, *J* = 8.5 Hz, 1H), 7.98 (d, *J* = 8.5 Hz, 1H), 7.74 (dd, *J* = 8.5, 1.0 Hz, 1H), 7.67 (ddd, *J* = 8.5, 7.0, 1.0 Hz, 1H), 7.54 (ddd, *J* = 8.5, 7.0, 1.5 Hz, 1H), 7.37 (dd, *J* = 8.5, 1.0 Hz, 1H), 3.95 (s, 3H), 2.71 (s, 3H). ^{13}C NMR ($CDCl_3$) δ 154.2, 145.4, 139.6, 138.2, 134.9, 130.1, 129.5, 129.1, 128.6, 128.3, 126.5, 125.6, 122.4, 121.7, 117.6, 117.5, 110.1, 60.6, 25.2. Mp. 176.0–176.4 °C. TLC: R_f 0.37 (hexane/$CHCl_3$ = 3:2). IR (KBr): 2941, 2853, 2234, 1461, 1352, 1074, 1031, 943, 868, 820 cm^{-1}. HRMS Calcd for $C_{19}H_{12}Br_3NONa$: $[M+Na]^+$, 531.8341. Found: *m*/*z* 531.8343. HPLC (Daicel Chiralpak IE, hexane/*i*-PrOH = 98.0/2.0, flow rate = 1.5 mL/min, λ = 254 nm, 40 °C): t_{minor} = 10.2 min, t_{major} = 8.0 min.

1-(2,4,6-Tribromo-3-isopropyl-5-methoxyphenyl)-2-naphthonitrile (5d).
Rotamers resulting from the isopropyl group were ovserved in the NMR analysis.

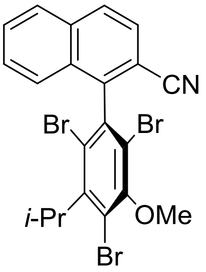

Yield of **2d**: 93% (48.8 mg), 60% *ee*, white solid. $[\alpha]_D^{20}$ 0.0 (*c* 0.63, CH_2Cl_2). ^{1}H NMR ($CDCl_3$) δ 8.00 (d, *J* = 8.5 Hz, 1H), 7.97 (d, *J* = 8.5 Hz, 1H), 7.73 (dd, *J* = 8.5, 1.0 Hz, 1H), 7.66 (ddd, *J* = 8.5, 7.0, 1.0 Hz, 1H), 7.55 (ddd, *J* = 8.5, 7.0, 1.0 Hz, 1H), 7.37 (dd, *J* = 8.5, 1.0 Hz, 1H), 4.10 (m, 1H), 3.95 (s, 3H), 1.50 (m, 6H). ^{13}C NMR ($CDCl_3$) δ 154.9, 154.2, 146.7, 146.5, 146.1, 145.8, 139.7, 138.8, 134.8 (2C), 130.3, 130.1, 129.8, 129.4, 129.0, 128.6, 128.3, 126.6, 125.7, 124.3, 123.1, 120.5, 119.4, 117.9, 117.8, 117.5 (2C), 110.3, 110.0, 60.7, 60.5, 37.0, 36.7, 19.43, 19.39, 19.3, 19.2. Mp. 65.6–66.6 °C. TLC: R_f 0.20 (hexane/$CHCl_3$ = 2:1). IR (KBr): 2933, 2853, 2228, 1444, 1353, 1314, 1063, 1006, 939, 820 cm^{-1}. HRMS Calcd for $C_{21}H_{16}Br_3NONa$: $[M+Na]^+$, 559.8654. Found: *m*/*z* 559.8656. HPLC (Daicel Chiralpak IE, hexane/*i*-PrOH = 98.0/2.0, flow rate = 1.0

mL/min, λ = 254 nm, 40 °C): t_{minor} = 11.1 min, t_{major} = 10.0 min.

2,4,6-Tribromo-3-(2-cyanonaphthalen-1-yl)-5-methoxyphenyl acetate (6e).

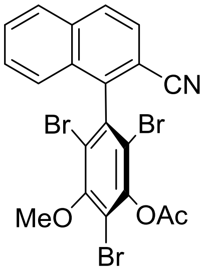

Yield of **2e**: 99% (53.5 mg), 19% *ee*, white solid. $[\alpha]_D^{20}$ –0.7 (*c* 0.92, CH_2Cl_2). ^1H NMR ($CDCl_3$) δ 8.03 (d, *J* = 8.5 Hz, 1H), 7.98 (d, *J* = 8.5 Hz, 1H), 7.74 (dd, *J* = 8.5, 1.0 Hz, 1H), 7.68 (ddd, *J* = 8.5, 7.0, 1.0 Hz, 1H), 7.67 (ddd, *J* = 8.5, 7.0, 1.0 Hz, 1H), 7.40 (dd, *J* = 8.5, 1.0 Hz, 1H), 3.99 (s, 3H), 2.42 (s, 3H). ^{13}C NMR ($CDCl_3$) δ 166.8, 155.3, 147.2, 143.8, 138.6, 134.8, 130.0, 129.9, 129.2, 128.61, 128.58, 126.4, 125.6, 117.9, 117.3, 115.5, 115.2, 110.2, 61.0, 20.5. Mp. 146.6–147.2 °C. TLC: R_f 0.30 (hexane/$CHCl_3$ = 1:1). IR (KBr): 2941, 2227, 1782, 1365, 1182, 1077, 960, 867, 824 cm^{-1}. HRMS Calcd for $C_{20}H_{12}Br_3NO_3Na$: $[M+Na]^+$, 575.8239. Found: *m*/*z* 575.8239. HPLC (Daicel Chiralpak IE, hexane/*i*-PrOH = 98.0/2.0, flow rate = 1.0 mL/min, λ = 254 nm, 40 °C): t_{minor} = 25.6 min, t_{major} = 30.1 min.

1-(2,6-Dibromo-4-fluoro-3-methoxyphenyl)-2-naphthonitrile (5f).

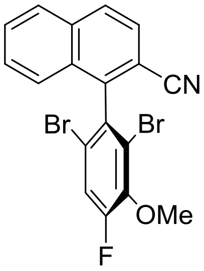

Yield of **2f**: 96% (40.5 mg), 16% *ee*, white solid. $[\alpha]_D^{20}$ –4.7 (*c* 0.88, CH_2Cl_2). ^1H NMR ($CDCl_3$) δ 8.02 (d, *J* = 8.5 Hz, 1H), 7.98 (d, *J* = 8.5 Hz, 1H), 7.74 (dd, *J* = 8.5, 1.0 Hz, 1H), 7.67 (ddd, *J* = 8.5, 7.0, 1.0 Hz, 1H), 7.57 (s, 1H), 7.55 (ddd, *J* = 8.5, 7.0, 1.0 Hz, 1H), 7.36 (dd, *J* = 8.5, 1.0 Hz, 1H), 4.06 (s, 3H). ^{13}C NMR ($CDCl_3$) δ 155.4 (d, *J* = 256.1 Hz), 145.5 (d, *J* = 12.6 Hz), 144.1, 134.8, 134.7 (d, *J* = 4.8 Hz), 130.4, 129.7, 129.1, 128.6, 128.3, 126.5, 125.6, 120.9 (d, *J* = 23.0 Hz), 120.3 (q, *J* = 3.8 Hz), 117.5, 117.3 (d, *J* = 9.7 Hz), 110.6, 61.7 (d, *J* = 4.9 Hz). ^{19}F NMR ($CDCl_3$) δ 37.8. Mp. 182.8–183.8 °C. TLC: R_f 0.30 (hexane/$CHCl_3$ = 3:2). IR (KBr): 3081, 2224, 1561, 1478, 1355, 1331, 1292, 1199, 1054, 981, 936, 875, 849, 821 cm^{-1}. HRMS Calcd for $C_{18}H_{10}Br_2FNONa$:

[M+Na]$^+$, 457.8985. Found: *m/z* 457.8989. HPLC (Daicel Chiralpak IB, hexane/*i*-PrOH = 98.5/1.5, flow rate = 0.5 mL/min, λ = 254 nm, 40 °C): t_{minor} = 17.0 min, t_{major} = 20.3 min.

1-(2,6-Dibromo-4-chloro-3-methoxyphenyl)-2-naphthonitrile (5g).

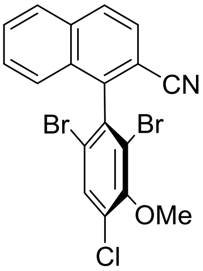

Yield of **2g**: 96% (42.1 mg), 36% *ee*, white solid. $[\alpha]_D^{20}$ –4.4 (*c* 1.04, CH_2Cl_2). ^{1}H NMR ($CDCl_3$) δ 8.03 (d, *J* = 8.5 Hz, 1H), 7.98 (d, *J* = 8.5 Hz, 1H), 7.83 (s, 1H), 7.75 (dd, *J* = 8.0, 1.0 Hz, 1H), 7.68 (ddd, *J* = 8.0, 7.0, 1.0 Hz, 1H), 7.56 (ddd, *J* = 8.0, 7.0, 1.0 Hz, 1H), 7.37 (dd, *J* = 8.0, 1.0 Hz, 1H), 3.97 (s, 3H). ^{13}C NMR ($CDCl_3$) δ 153.6, 144.0, 138.0, 134.8, 133.2, 130.6, 130.1, 129.8, 129.1, 128.6, 128.4, 126.5, 125.5, 121.5, 118.9, 117.4, 110.3, 60.9. Mp. 149.6–150.0 °C. TLC: R_f 0.37 (hexane/$CHCl_3$ = 3:2). IR (KBr): 3072, 2933, 2225, 1448, 1382, 1346, 1254, 1053, 985, 979, 867, 822 cm^{-1}. HRMS Calcd for $C_{18}H_{10}Br_2ClNONa$: [M+Na]$^+$, 473.8689. Found: *m/z* 473.8691. HPLC (Daicel Chiralpak IB, hexane/*i*-PrOH = 98.5/1.5, flow rate = 0.5 mL/min, λ = 254 nm, 40 °C): t_{minor} = 16.1 min, t_{major} = 18.3 min.

2,4-Dibromo-3-(2-cyanonaphthalen-1-yl)-6-methylphenyl acetate (6h).

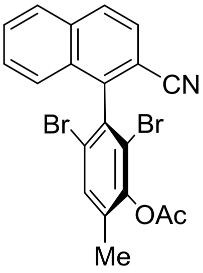

Yield of **2h**: 86% (38.5 mg), 84% *ee*, white solid. $[\alpha]_D^{20}$ –8.2 (*c* 0.58, CH_2Cl_2). ^{1}H NMR ($CDCl_3$) δ 8.01 (d, *J* = 8.5 Hz, 1H), 7.96 (d, *J* = 8.5 Hz, 1H), 7.73 (dd, *J* = 8.5, 1.0 Hz, 1H), 7.67 (d, *J* = 0.5 Hz, 1H), 7.66 (ddd, *J* = 8.5, 7.0, 1.0 Hz, 1H), 7.55 (ddd, *J* = 8.5, 7.0, 1.0 Hz, 1H), 7.43 (dd, *J* = 8.5, 1.0 Hz, 1H), 2.39 (s, 3H), 2.34 (d, *J* = 0.5 Hz, 3H). ^{13}C NMR ($CDCl_3$) δ 167.8, 147.2, 144.3, 136.6, 135.1, 134.8, 134.1, 130.4, 129.6, 129.1, 128.5, 128.4, 126.5, 125.9, 121.0, 119.6, 117.5, 110.5, 20.5, 16.9. Mp. 210.0–211.0 °C. TLC: R_f 0.15 (hexane/$CHCl_3$ = 1:1). IR (KBr): 2934, 2228, 1769, 1452, 1371, 1152,

1008, 888, 869, 821 cm^{-1}. HRMS Calcd for $C_{20}H_{13}Br_2NO_2Na$: [M+Na]$^+$, 481.9185. Found: *m*/*z* 481.9177. HPLC (Daicel Chiralpak IE, hexane/*i*-PrOH = 97.0/3.0, flow rate = 1.5 mL/min, λ = 254 nm, 30 °C): t_{minor} = 16.6 min, t_{major} = 13.7 min.

1-(2,6-Dibromo-3,4-dimethoxyphenyl)-2-naphthonitrile (5i).

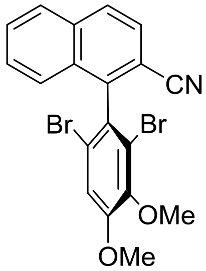

Yield of **2i**: 79% (34.3 mg), 38% *ee*, white solid. $[\alpha]_D^{20}$ –3.1 (*c* 0.26, CH_2Cl_2). ^1H NMR ($CDCl_3$) δ 8.00 (d, *J* = 8.5 Hz, 1H), 7.96 (d, *J* = 8.5 Hz, 1H), 7.73 (dd, *J* = 8.0, 1.0 Hz, 1H), 7.65 (ddd, *J* = 8.0, 6.5, 1.0 Hz, 1H), 7.54 (ddd, *J* = 8.0, 6.5, 1.0 Hz, 1H), 7.41 (dd, *J* = 8.0, 1.0 Hz, 1H), 7.30 (s, 1H), 3.98 (s, 3H), 3.93 (s, 3H). ^{13}C NMR ($CDCl_3$) δ 154.2, 146.5, 144.8, 134.8, 130.9, 130.2, 129.4, 128.9, 128.5, 128.1, 126.5, 126.0, 120.3, 118.5, 117.8, 115.9, 111.0, 60.8, 56.3. Mp. 189.5–190.5 °C. TLC: R_f 0.18 (hexane/$CHCl_3$ = 1:1). IR (KBr): 2942, 2231, 1582, 1483, 1298, 1022, 981, 857, 822 cm^{-1}. HRMS Calcd for $C_{19}H_{13}Br_2NO_2Na$: [M+Na]$^+$, 469.9185. Found: *m*/*z* 469.9191. HPLC (Daicel Chiralpak IE, hexane/*i*-PrOH = 98.5/1.5, flow rate = 1.5 mL/min, λ = 254 nm, 40 °C): t_{minor} = 35.0 min, t_{major} = 20.4 min.

1-(2-Bromo-3-hydroxyphenyl)-2-naphthonitrile (1a-Br).

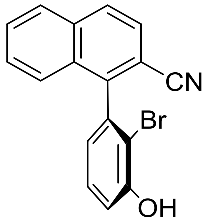

Yield: not determined, 75% *ee*, white solid. $[\alpha]_D^{20}$ –30.0 (*c* 0.54, CH_2Cl_2, 69% *ee*). ^1H NMR ($CDCl_3$) δ 7.99 (d, *J* = 8.5 Hz, 1H), 7.96 (d, *J* = 8.5 Hz, 1H), 7.72 (d, *J* = 8.5 Hz, 1H), 7.65 (dd, *J* = 8.5 Hz, 8.5 Hz, 1H), 7.52 (dd, *J* = 8.5, 8.5, 1H), 7.47 (d, *J* = 8.5 Hz, 1H), 7.41 (dd, *J* = 8.5, 7.5 Hz, 1H), 7.21 (d, *J* = 8.5 Hz, 1H), 6.95 (d, *J* = 7.5 Hz, 1H), 5.83 (br s, 1H). ^{13}C NMR ($CDCl_3$) δ 152.9, 144.6, 138.0, 134.7, 131.1, 129.2, 129.0, 128.9, 128.3, 127.9, 126.8, 126.4, 123.3, 118.0, 116.5, 112.0, 110.1. Mp. 171.5–172.5 °C. TLC: R_f 0.45 (toluene/Et_2O = 3:1). IR (KBr): 3393, 3072, 2231, 1570, 1468, 1388, 1298, 1037, 895, 817 cm^{-1}. HRMS Calcd for $C_{17}H_{10}BrNONa$: [M+Na]$^+$,

345.9838. Found: *m*/*z* 345.9847. HPLC (Daicel Chiralpak IB, hexane/*i*-PrOH = 95.0/5.0, flow rate = 1.0 mL/min, λ = 254 nm, 40 °C): t_{minor} = 16.4 min, t_{major} = 18.2 min.

2-Ethynyl-1-(2,4,6-tribromo-3-methoxyphenyl)naphthalene.

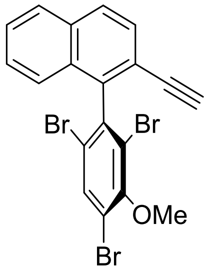

Yield of **2a**: 71% (34.1 mg), 12% *ee*, white solid. $[\alpha]_D^{20}$ –1.5 (*c* 0.46, CH_2Cl_2). ^1H NMR ($CDCl_3$) δ 7.96 (s, 1H), 7.91 (dd, *J* = 8.0, 1.0 Hz, 1H), 7.90 (d, *J* = 8.0 Hz, 1H), 7.66 (d, *J* = 8.0 Hz, 1H), 7.54 (ddd, *J* = 8.0, 7.0, 1.0 Hz, 1H), 7.46 (ddd, *J* = 8.0, 7.0, 1.0 Hz, 1H), 7.26 (dd, *J* = 8.0, 1.0 Hz, 1H), 3.95 (s, 3H), 3.06 (s, 1H). ^{13}C NMR ($CDCl_3$) δ 154.2, 141.7, 141.0, 135.4, 133.1, 130.3, 128.8, 128.6, 128.4, 127.4, 127.1, 124.9, 121.6, 119.8, 119.3, 118.0, 81.7, 81.3, 60.8. Mp. 163.5–164.5 °C. TLC: R_f 0.25. (hexane). IR (KBr): 3296, 2928, 1448, 1341, 1244, 1049, 983, 872, 829 cm^{-1}. HRMS Calcd for $C_{19}H_{12}Br_3O$: $[M+H]^+$, 492.8433. Found: *m*/*z* 492.8428. HPLC (Daicel Chiralpak IB, hexane/*i*-PrOH = 99.2/0.8, flow rate = 0.5 mL/min, λ = 254 nm, 40 °C): t_{minor} = 13.5 min, t_{major} = 14.2 min.

1-(2,4,6-Tribromo-3-methoxyphenyl)-2-naphthonitrile (5a)

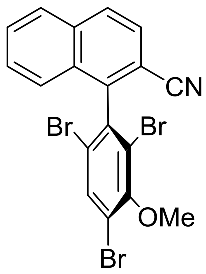

^{1}H NMR

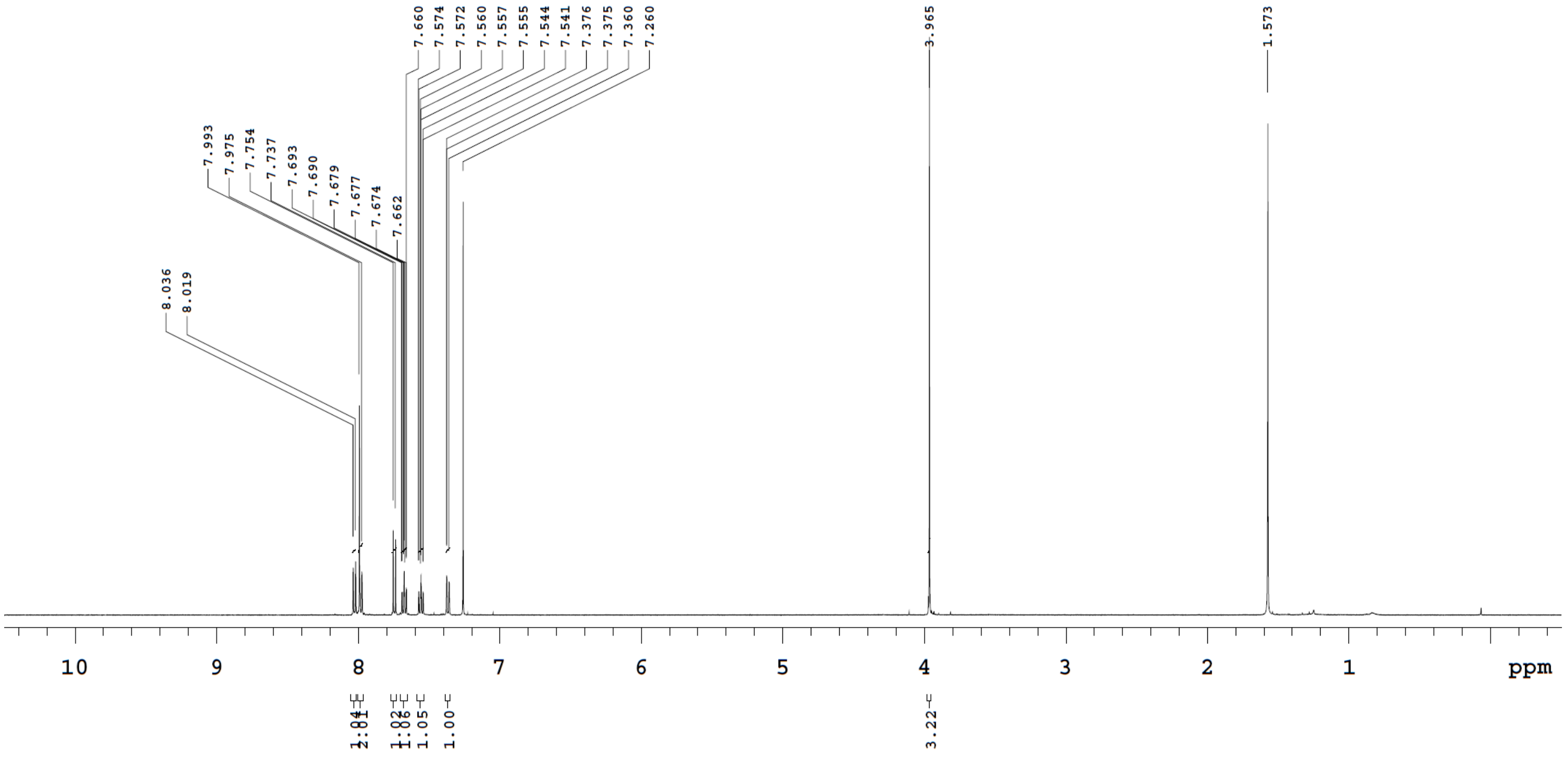

1-(2,4,6-Tribromo-3-methoxyphenyl)-2-naphthonitrile (5a)

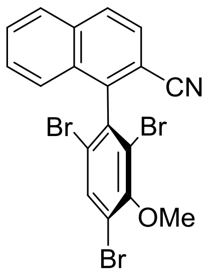

^{13}C NMR

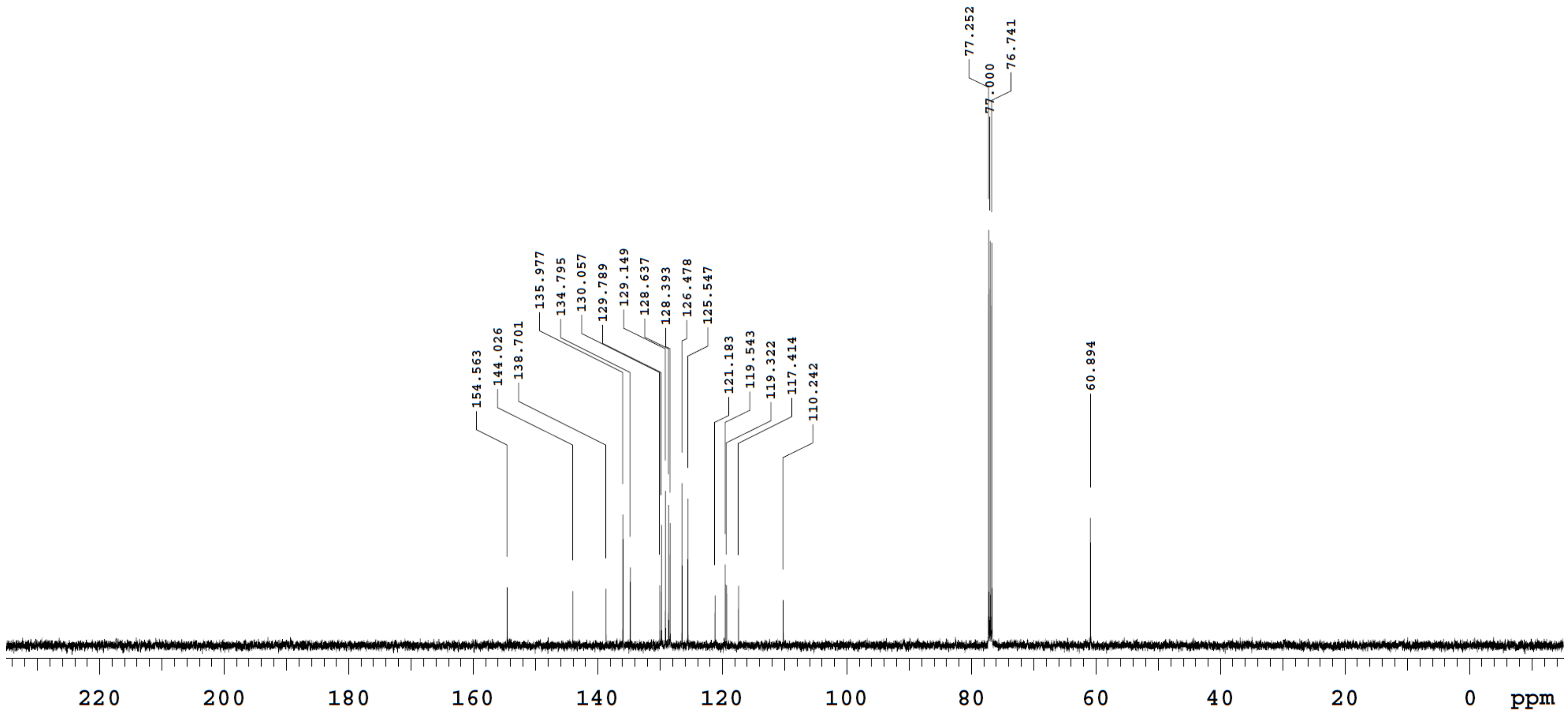

1-(2,4,6-Tribromo-3-methoxy-5-(trifluoromethyl)phenyl)-2-naphthonitrile (5b)

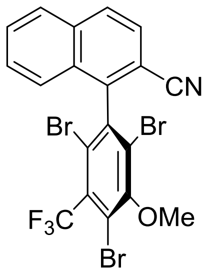

^{1}H NMR

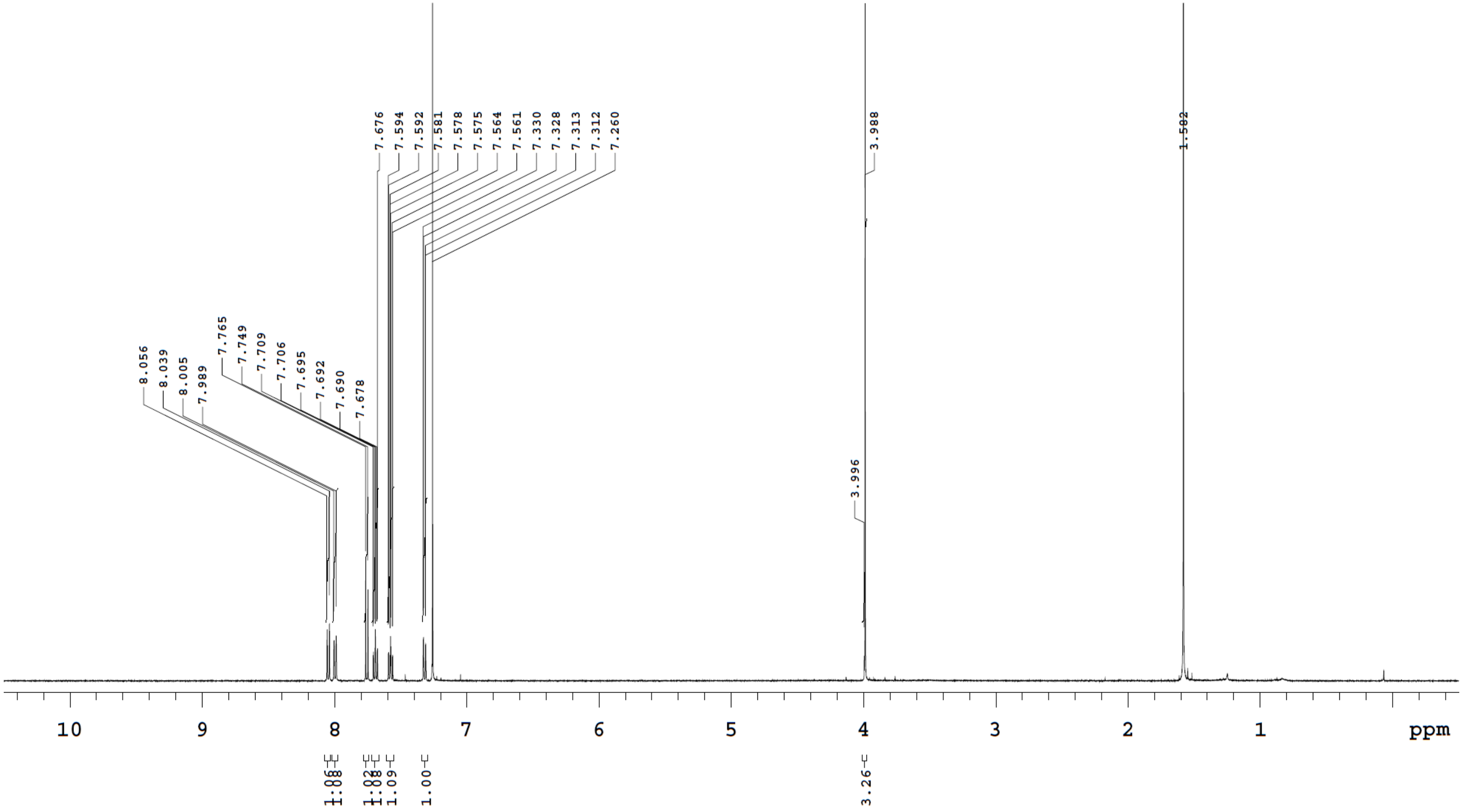

1-(2,4,6-Tribromo-3-methoxy-5-(trifluoromethyl)phenyl)-2-naphthonitrile (5b)

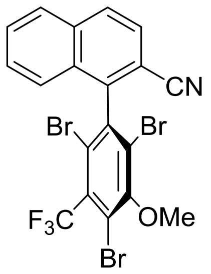

^{13}C NMR

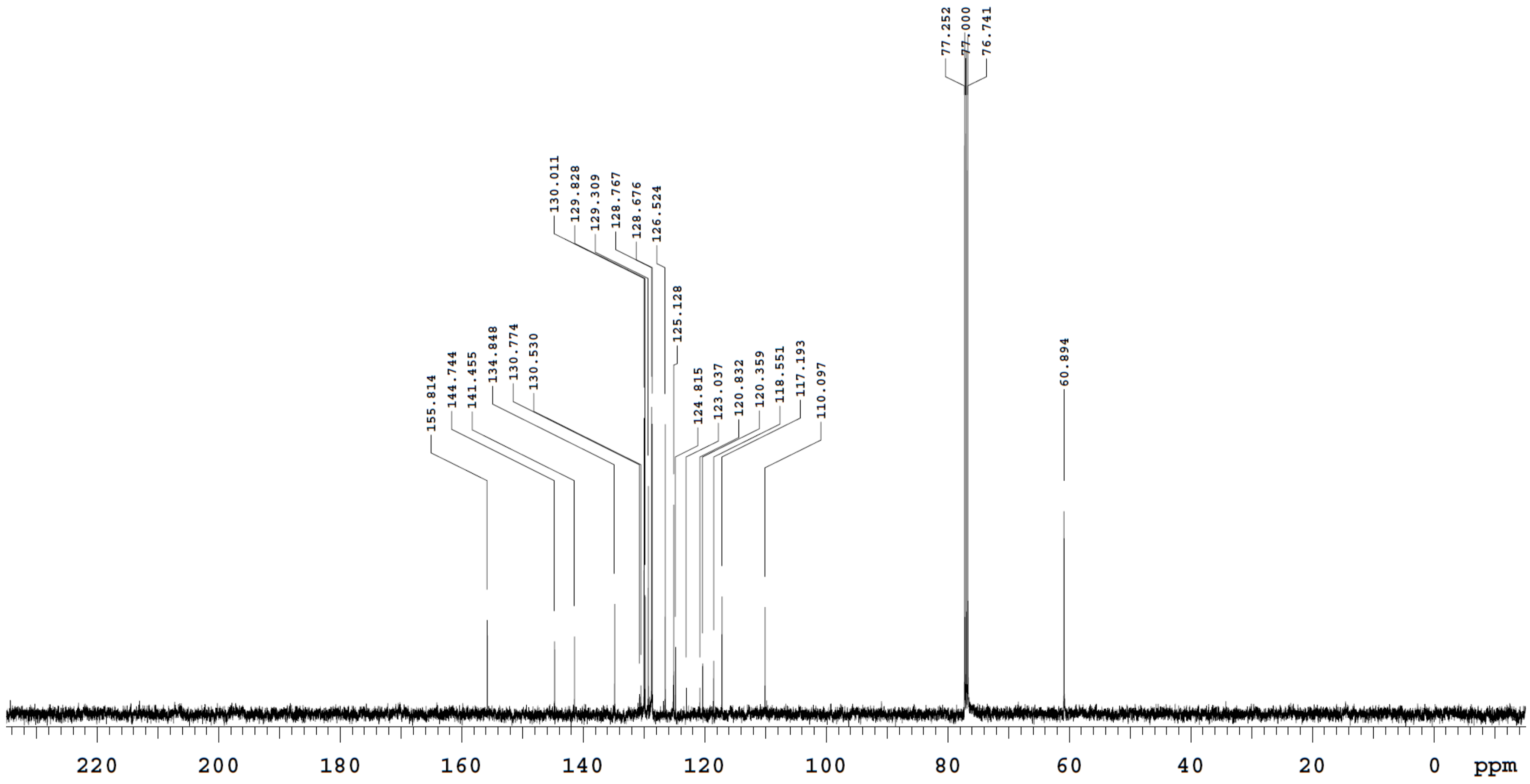

(*S*)-1-(2,4,6-Tribromo-3-methoxy-5-methylphenyl)-2-naphthonitrile (5c)

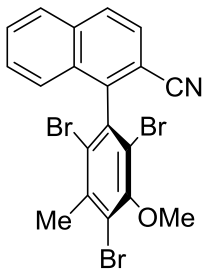

^{1}H NMR

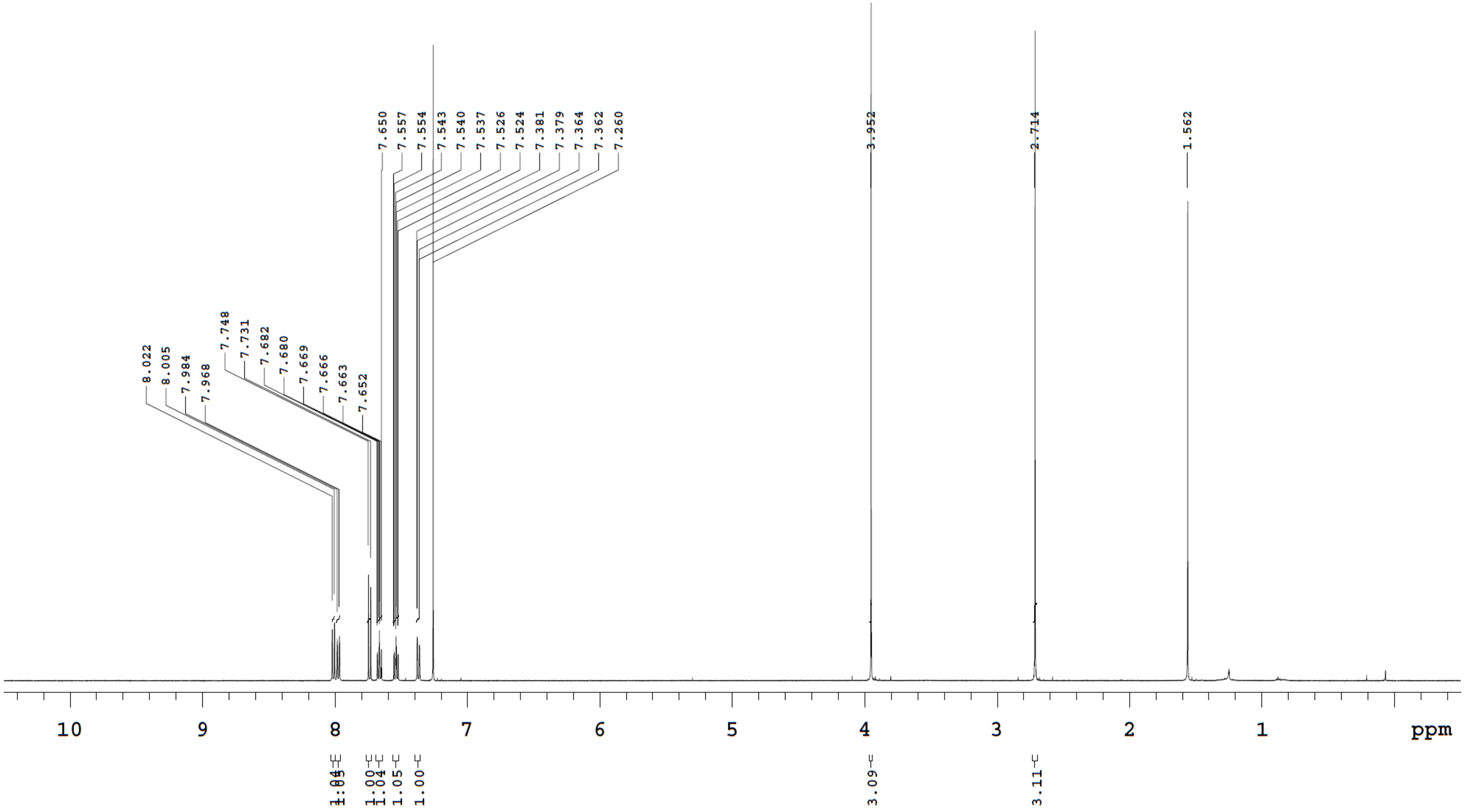

(*S*)-1-(2,4,6-Tribromo-3-methoxy-5-methylphenyl)-2-naphthonitrile (5c)

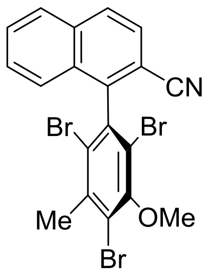

^{13}C NMR

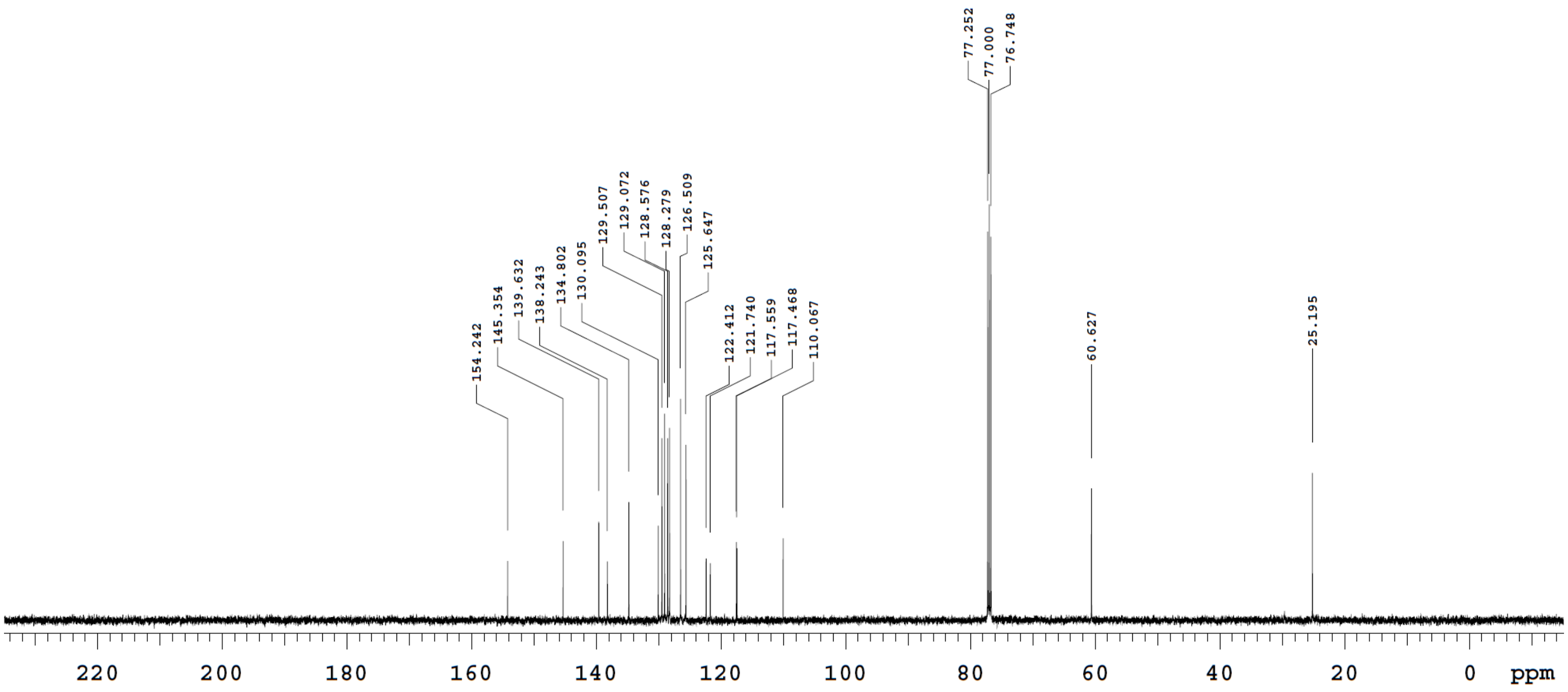

1-(2,4,6-Tribromo-3-isopropyl-5-methoxyphenyl)-2-naphthonitrile (5d)

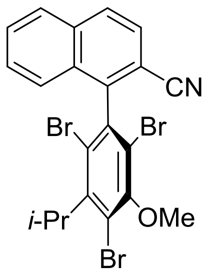

^{1}H NMR

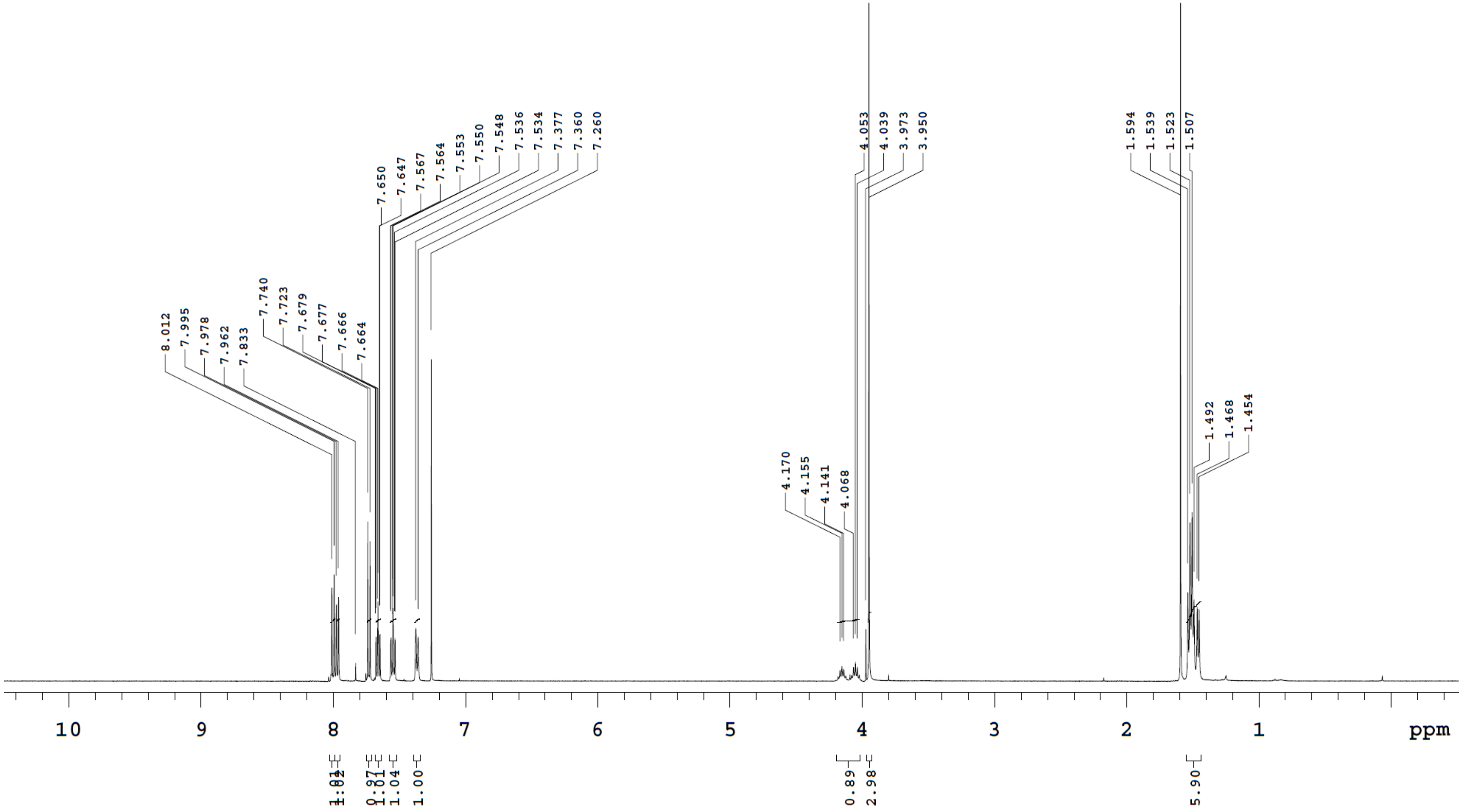

1-(2,4,6-Tribromo-3-isopropyl-5-methoxyphenyl)-2-naphthonitrile (5d)

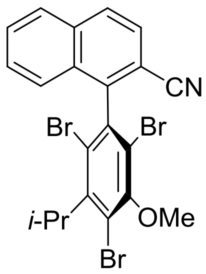

^{13}C NMR

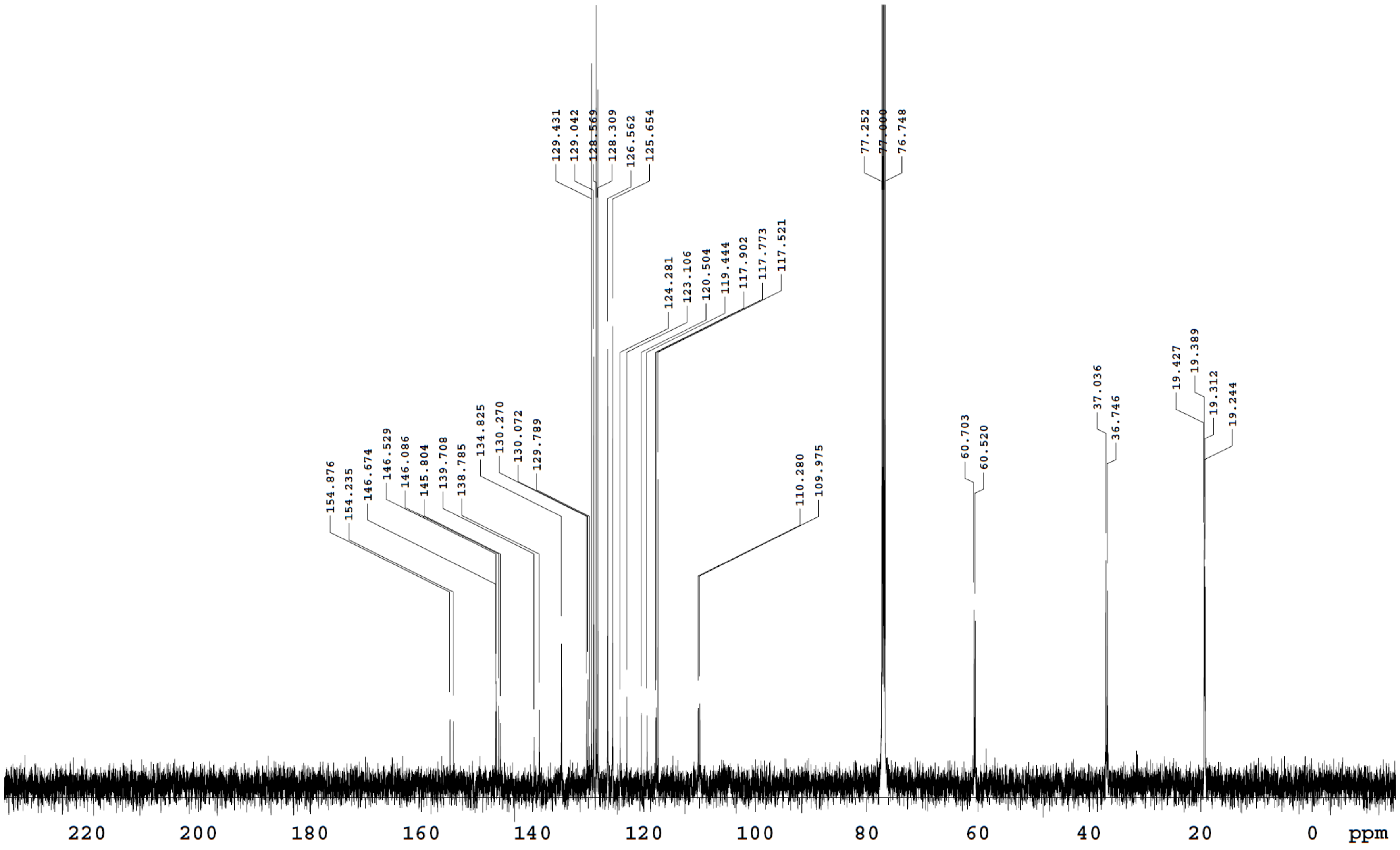

2,4,6-Tribromo-3-(2-cyanonaphthalen-1-yl)-5-methoxyphenyl acetate (6e)

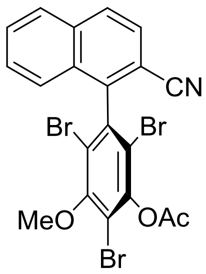

^{1}H NMR

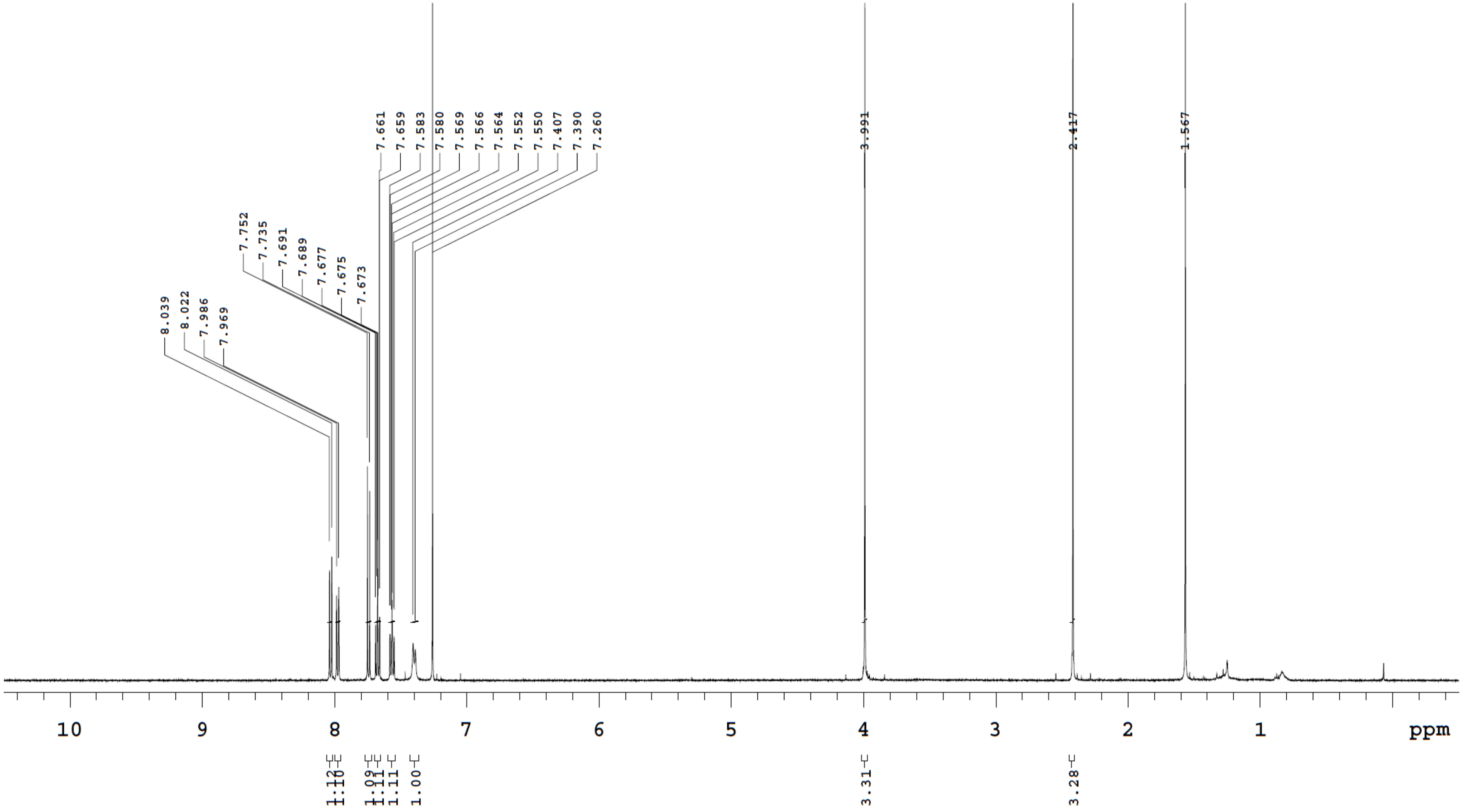

2,4,6-Tribromo-3-(2-cyanonaphthalen-1-yl)-5-methoxyphenyl acetate (6e)

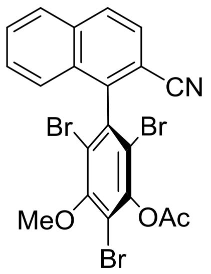

^{13}C NMR

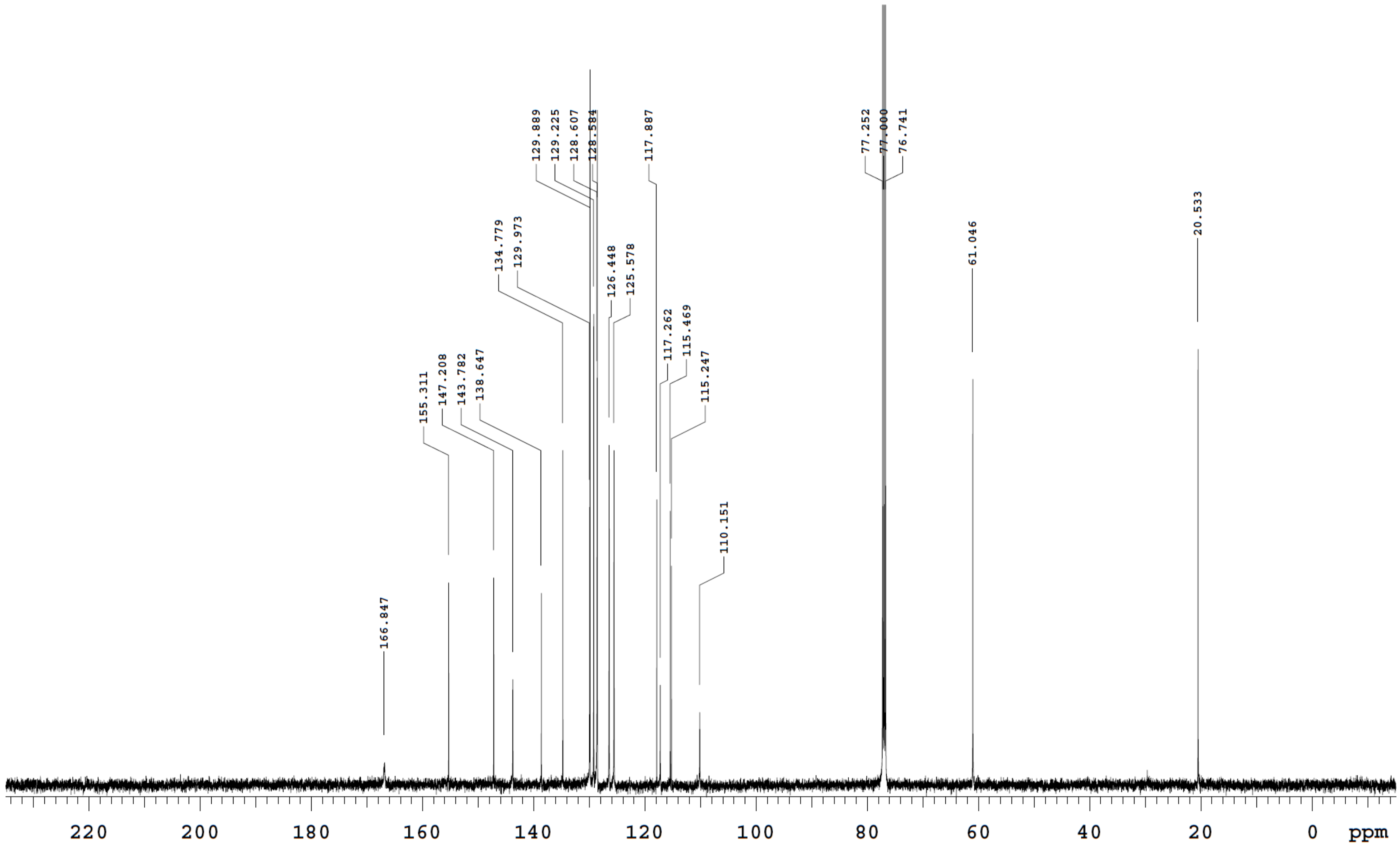

1-(2,6-Dibromo-4-fluoro-3-methoxyphenyl)-2-naphthonitrile (5f)

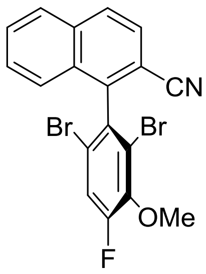

^{1}H NMR

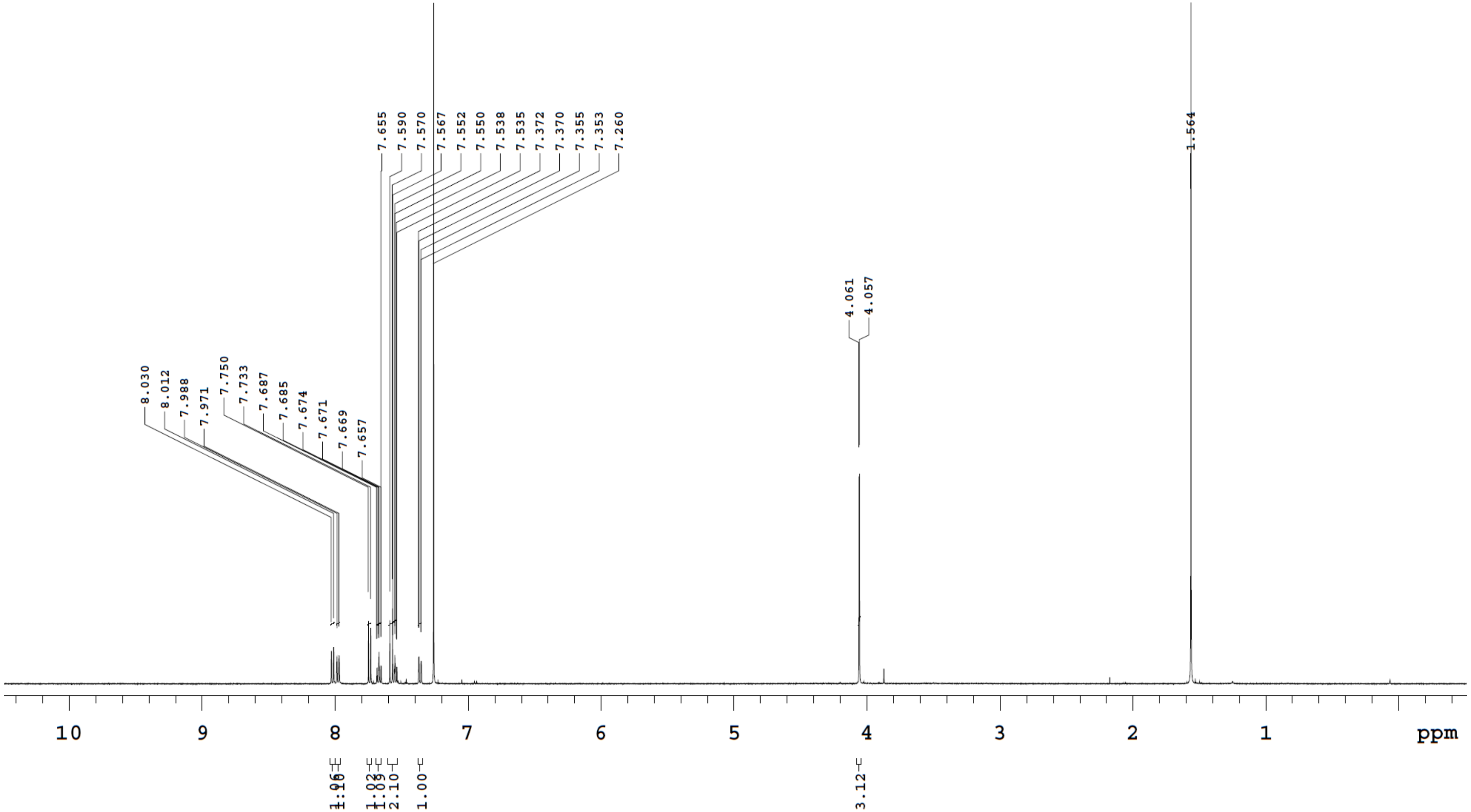

1-(2,6-Dibromo-4-fluoro-3-methoxyphenyl)-2-naphthonitrile (5f)

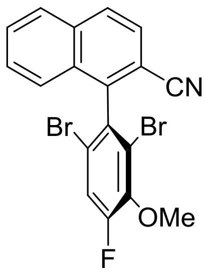

^{13}C NMR

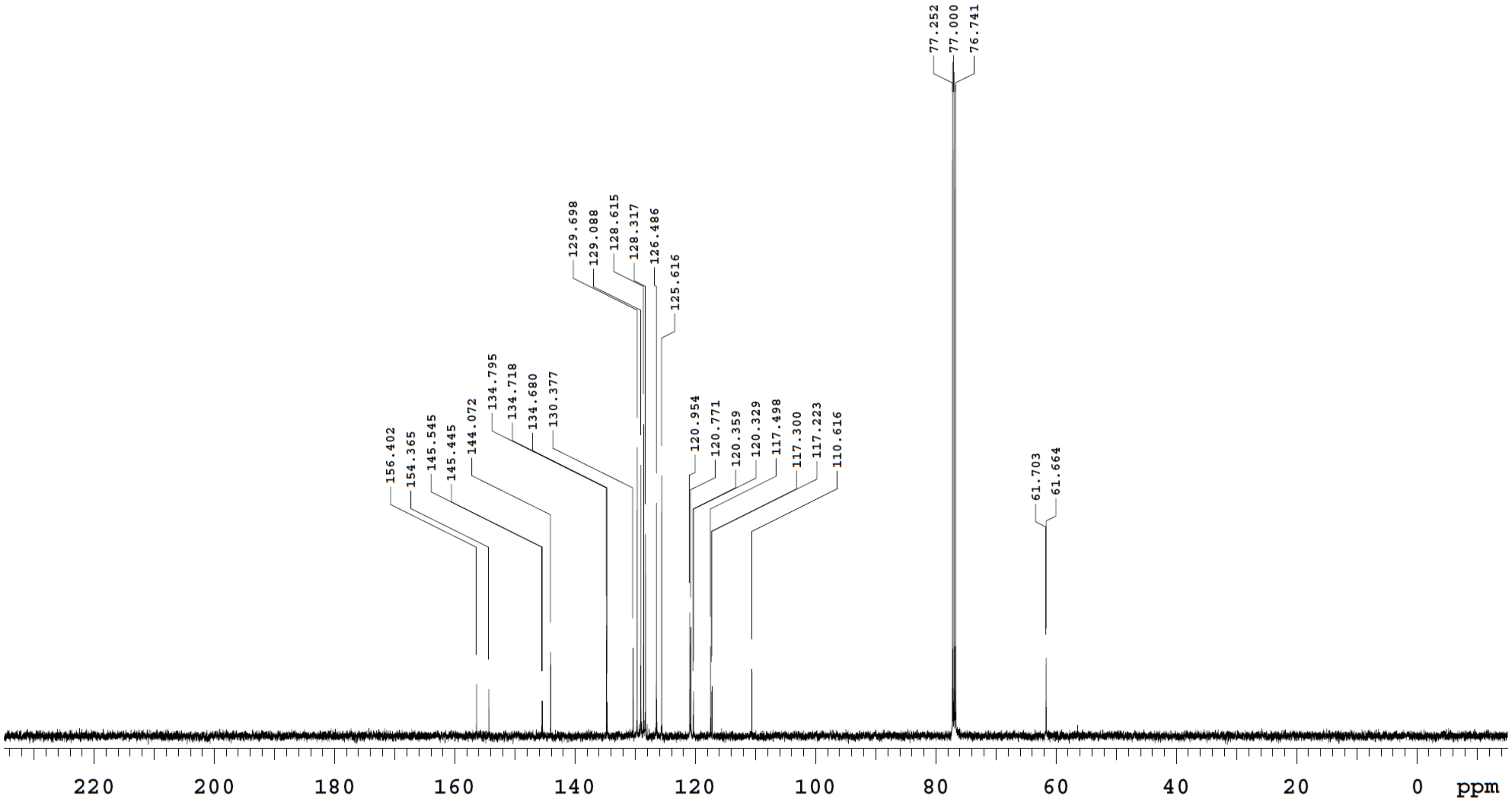

1-(2,6-Dibromo-4-chloro-3-methoxyphenyl)-2-naphthonitrile (5g)

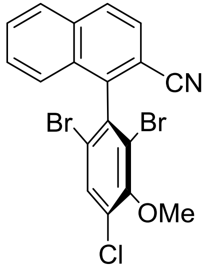

^{1}H NMR

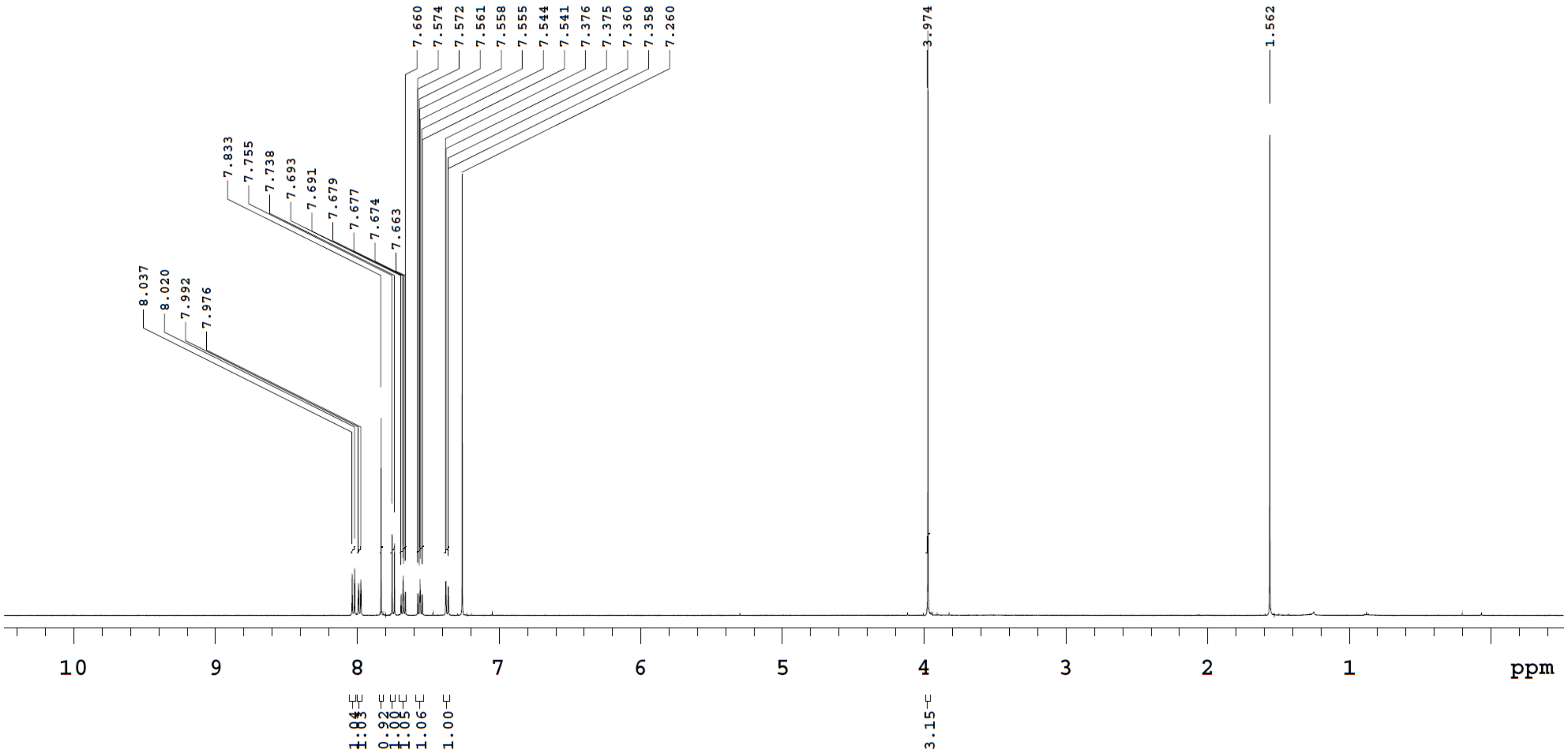

1-(2,6-Dibromo-4-chloro-3-methoxyphenyl)-2-naphthonitrile (5g)

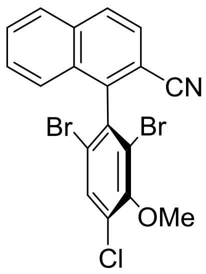

^{13}C NMR

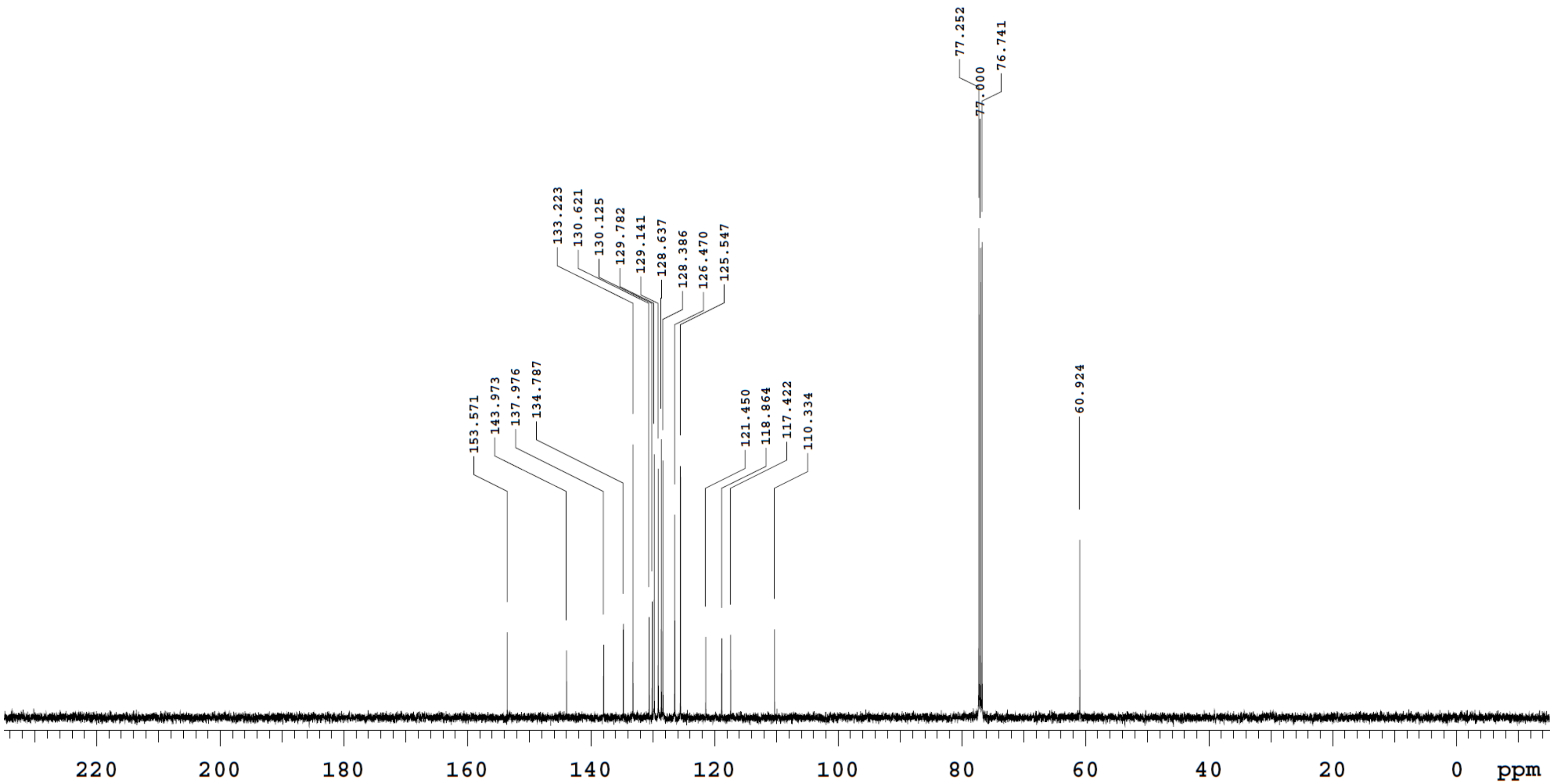

2,4-Dibromo-3-(2-cyanonaphthalen-1-yl)-6-methylphenyl acetate (6h)

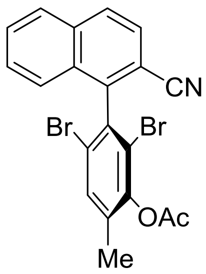

^{1}H NMR

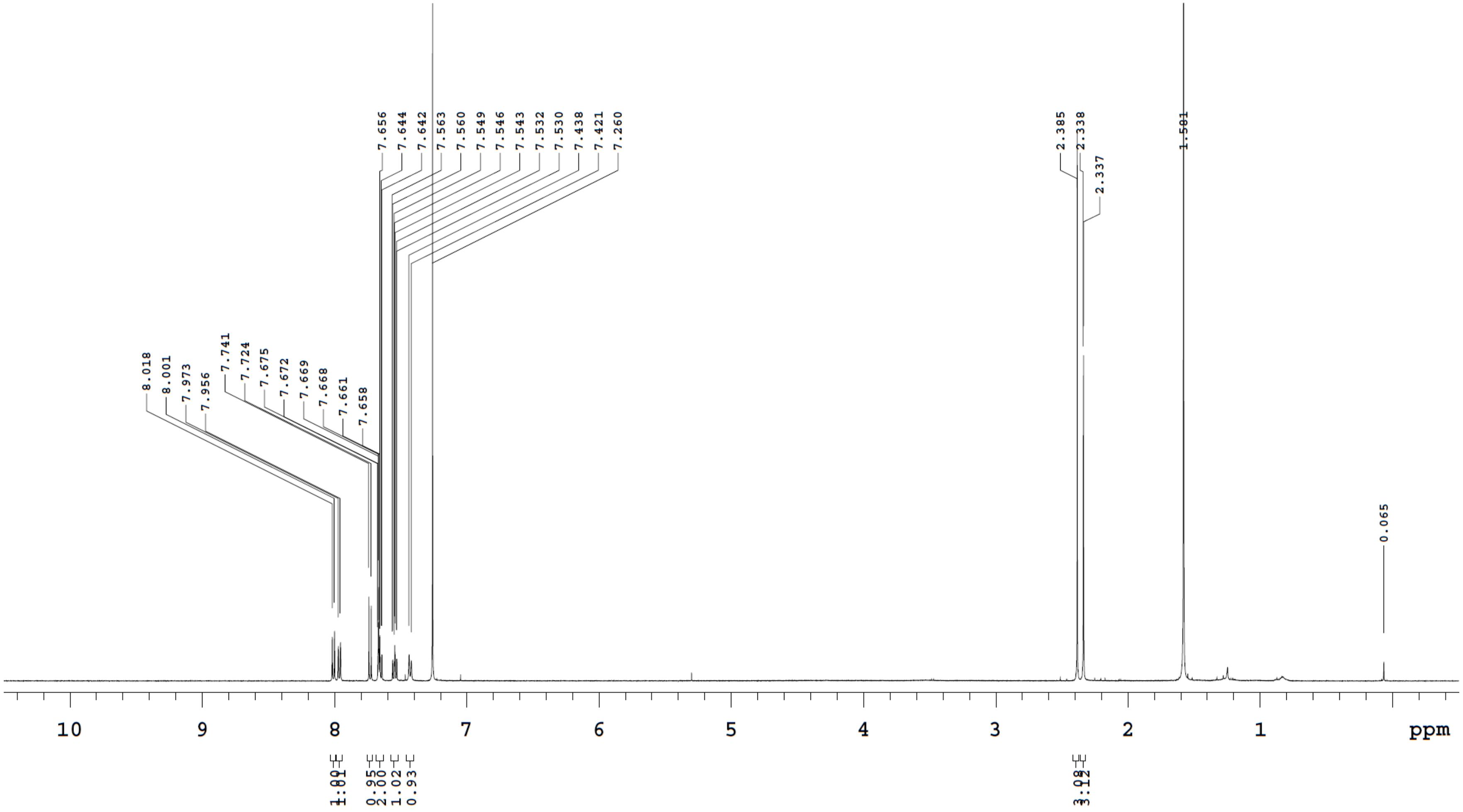

2,4-Dibromo-3-(2-cyanonaphthalen-1-yl)-6-methylphenyl acetate (6h)

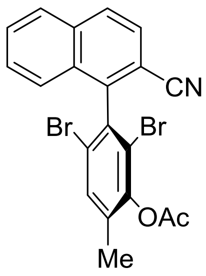

^{13}C NMR

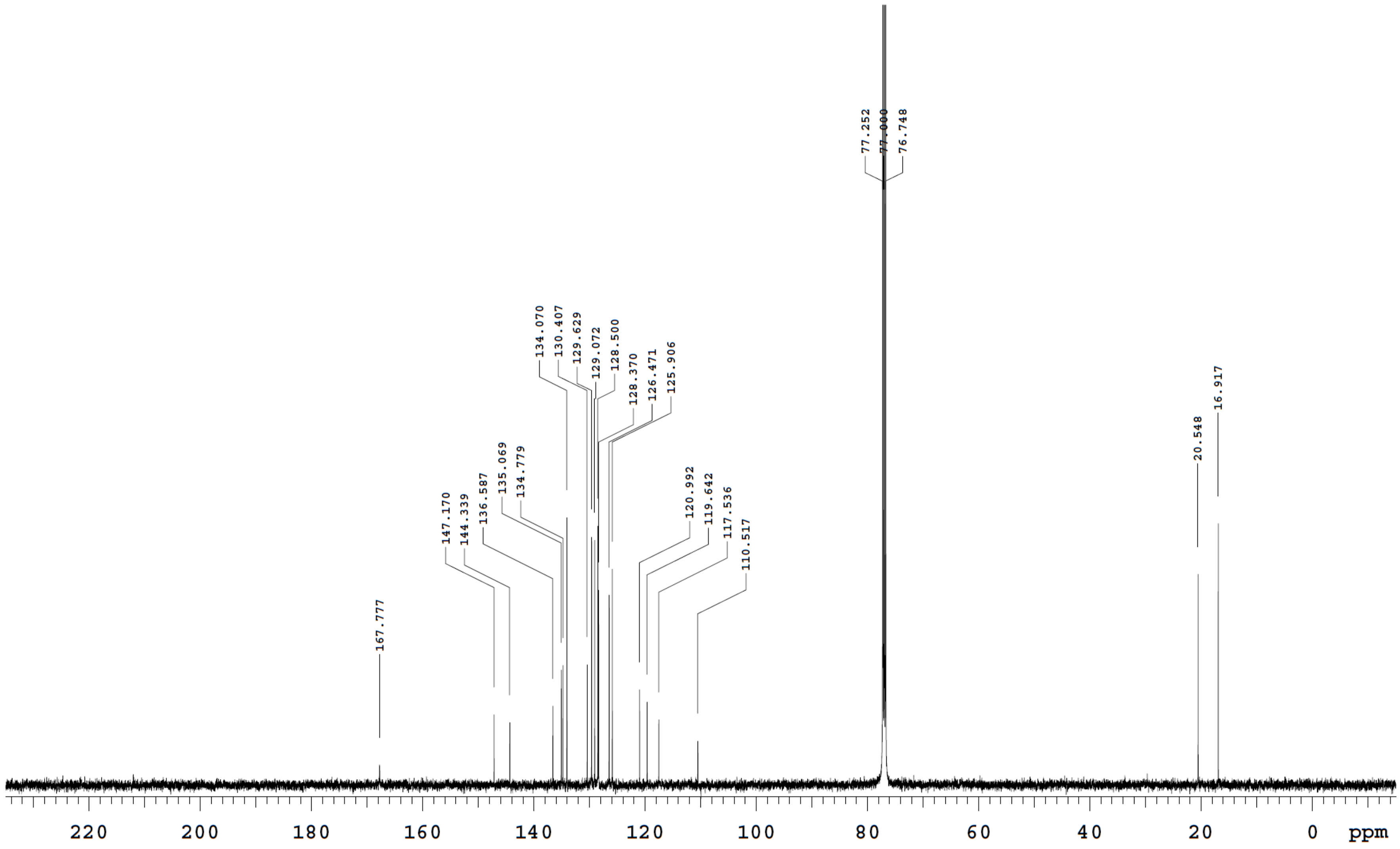

1-(2,6-Dibromo-3,4-dimethoxyphenyl)-2-naphthonitrile (5i)

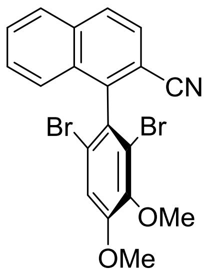

^{1}H NMR

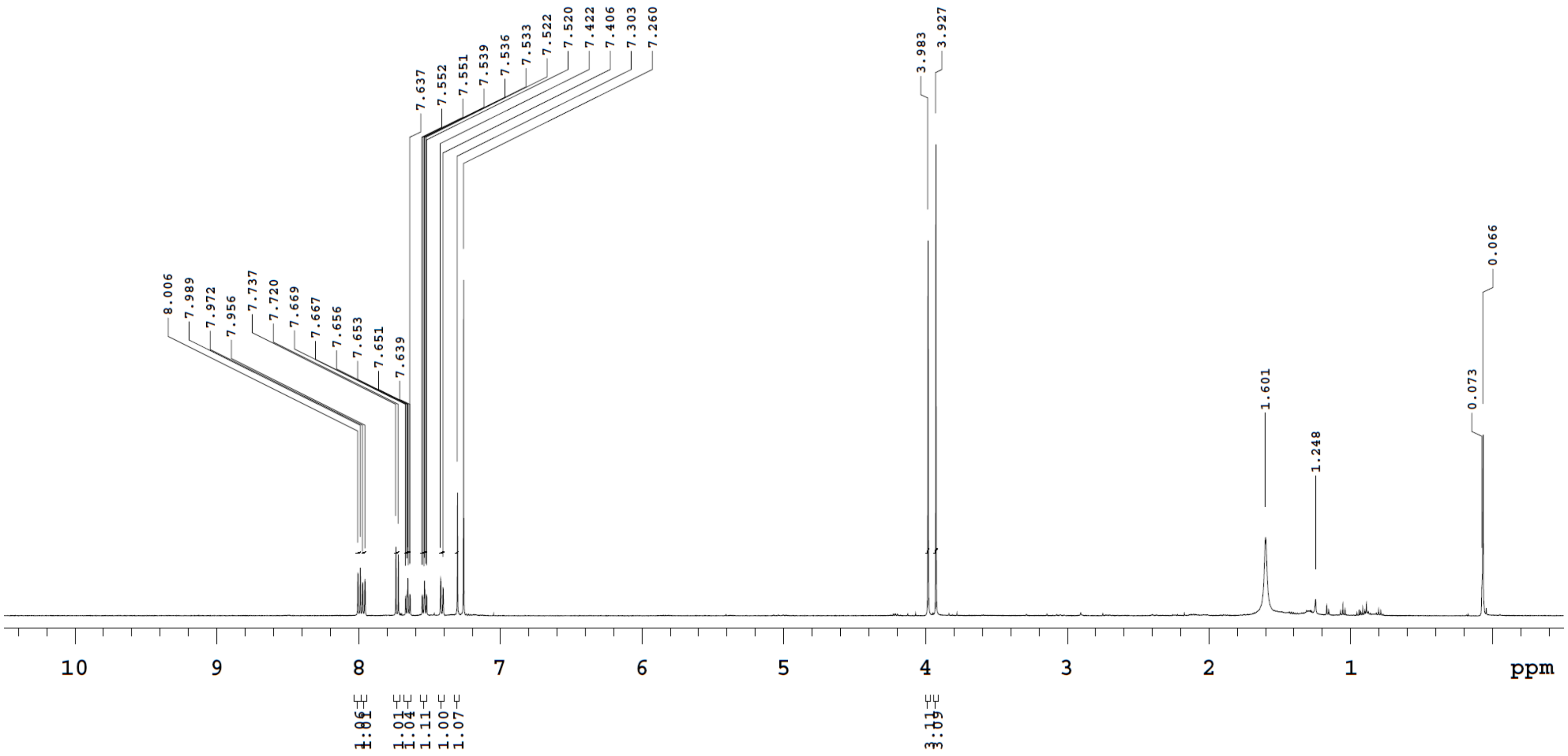

1-(2,6-Dibromo-3,4-dimethoxyphenyl)-2-naphthonitrile (5i)

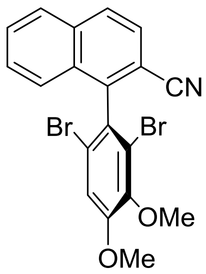

^{13}C NMR

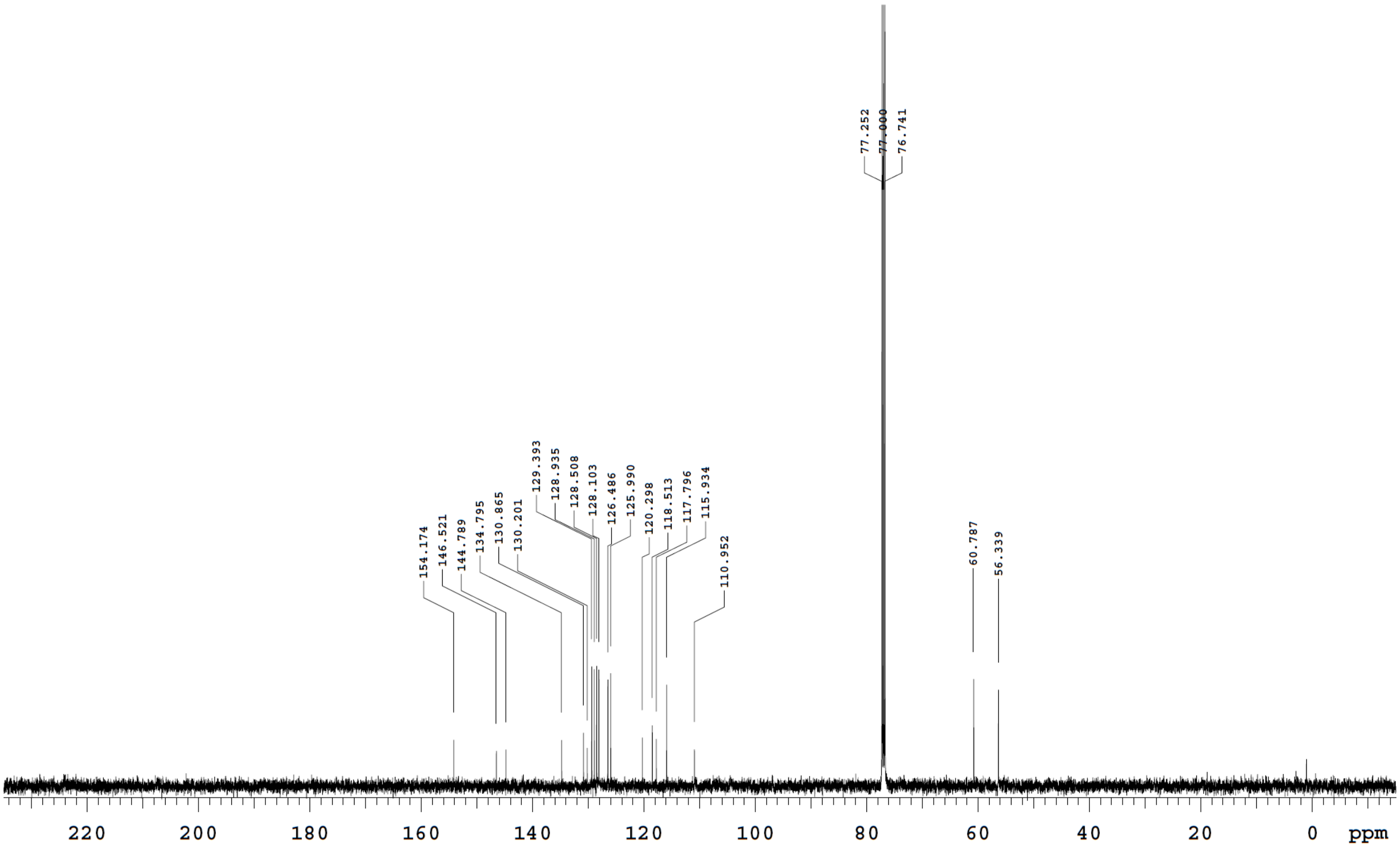

1-(2-Bromo-3-hydroxyphenyl)-2-naphthonitrile (1a-Br)

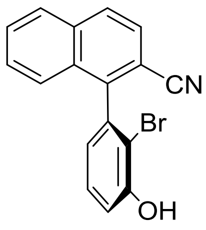

^{1}H NMR

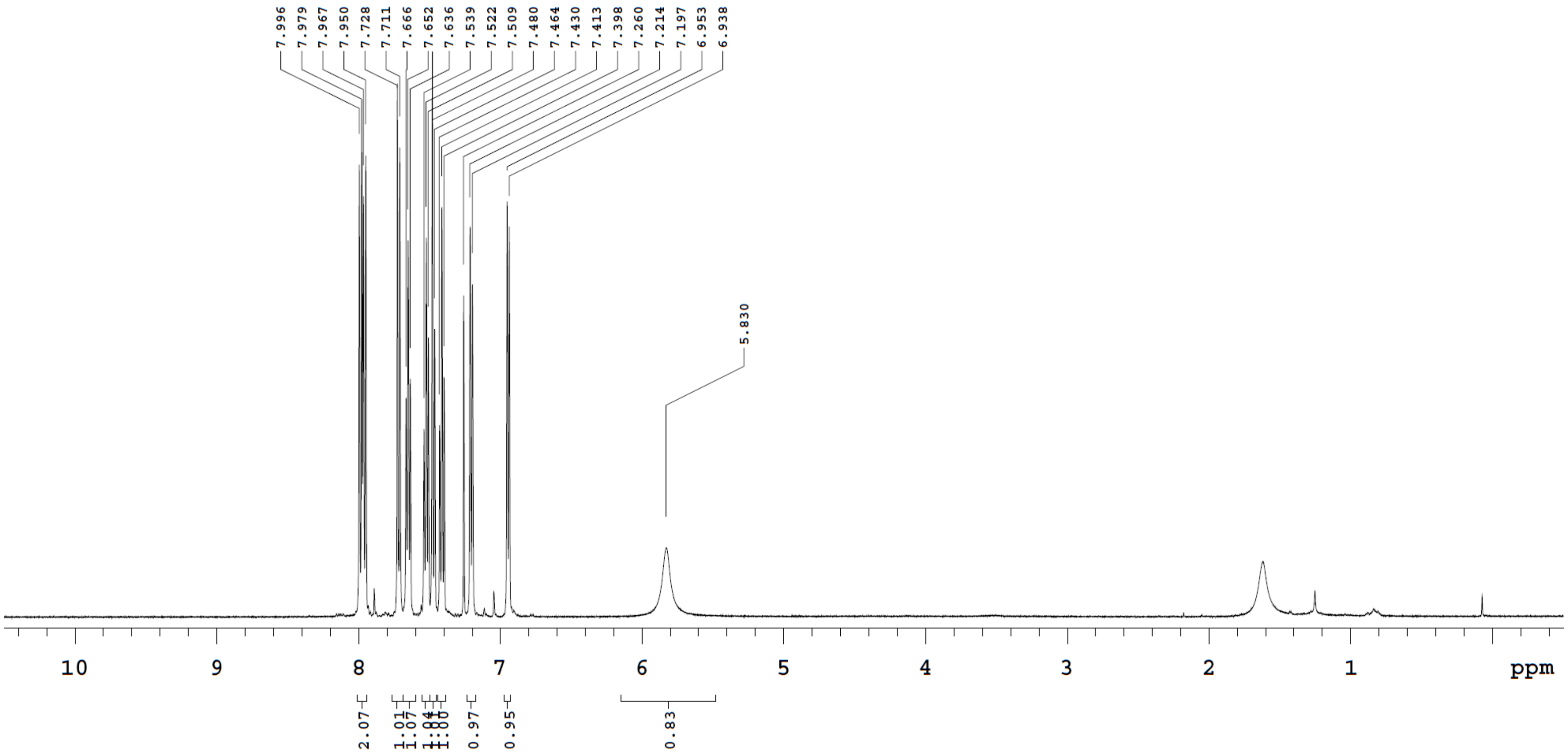

1-(2-Bromo-3-hydroxyphenyl)-2-naphthonitrile (1a-Br)

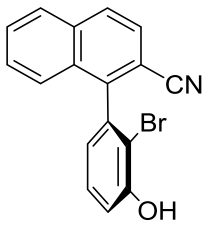

^{13}C NMR

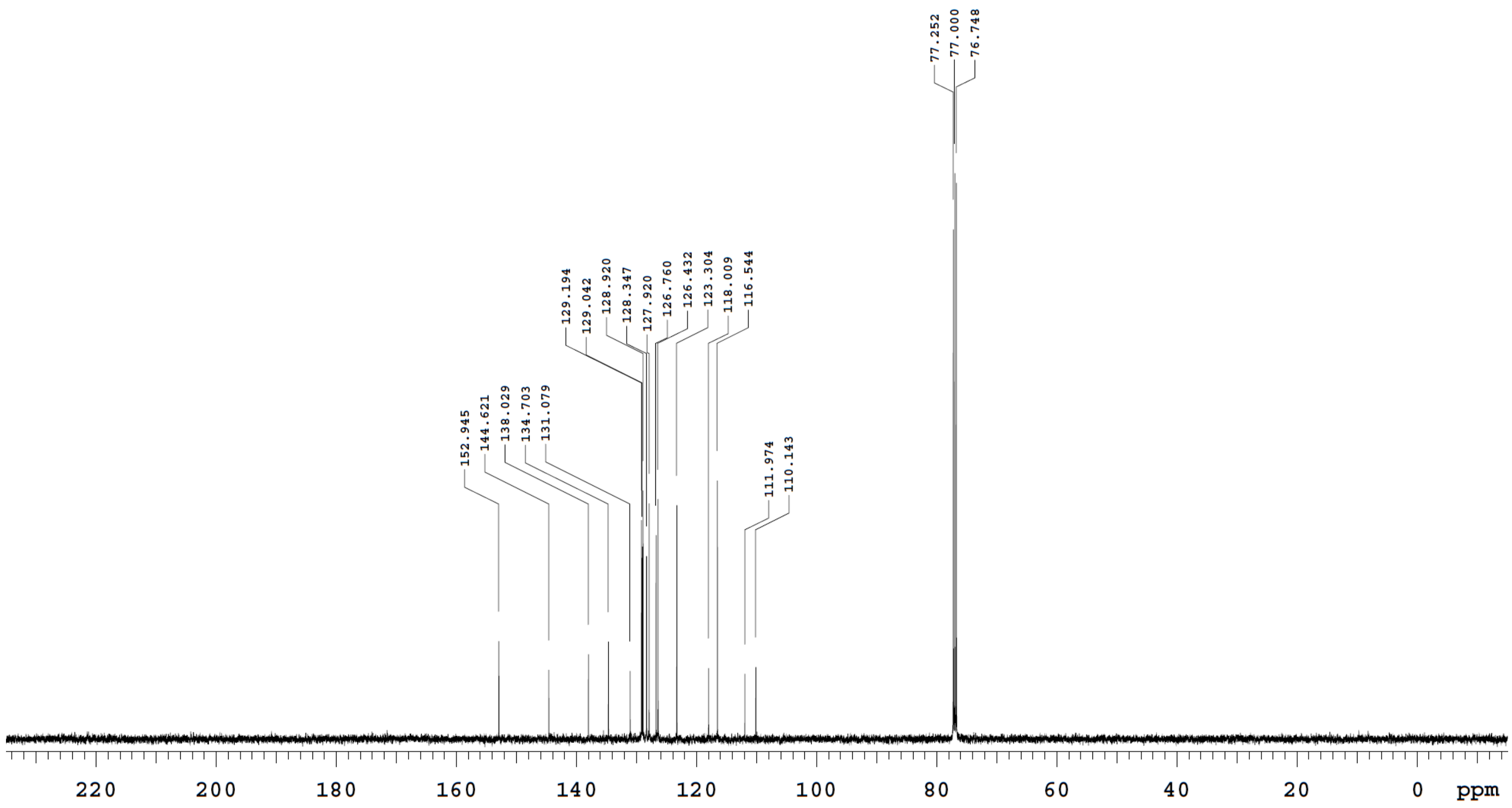

2-Ethynyl-1-(2,4,6-tribromo-3-methoxyphenyl)naphthalene

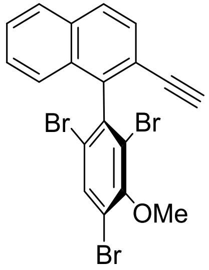

^{1}H NMR

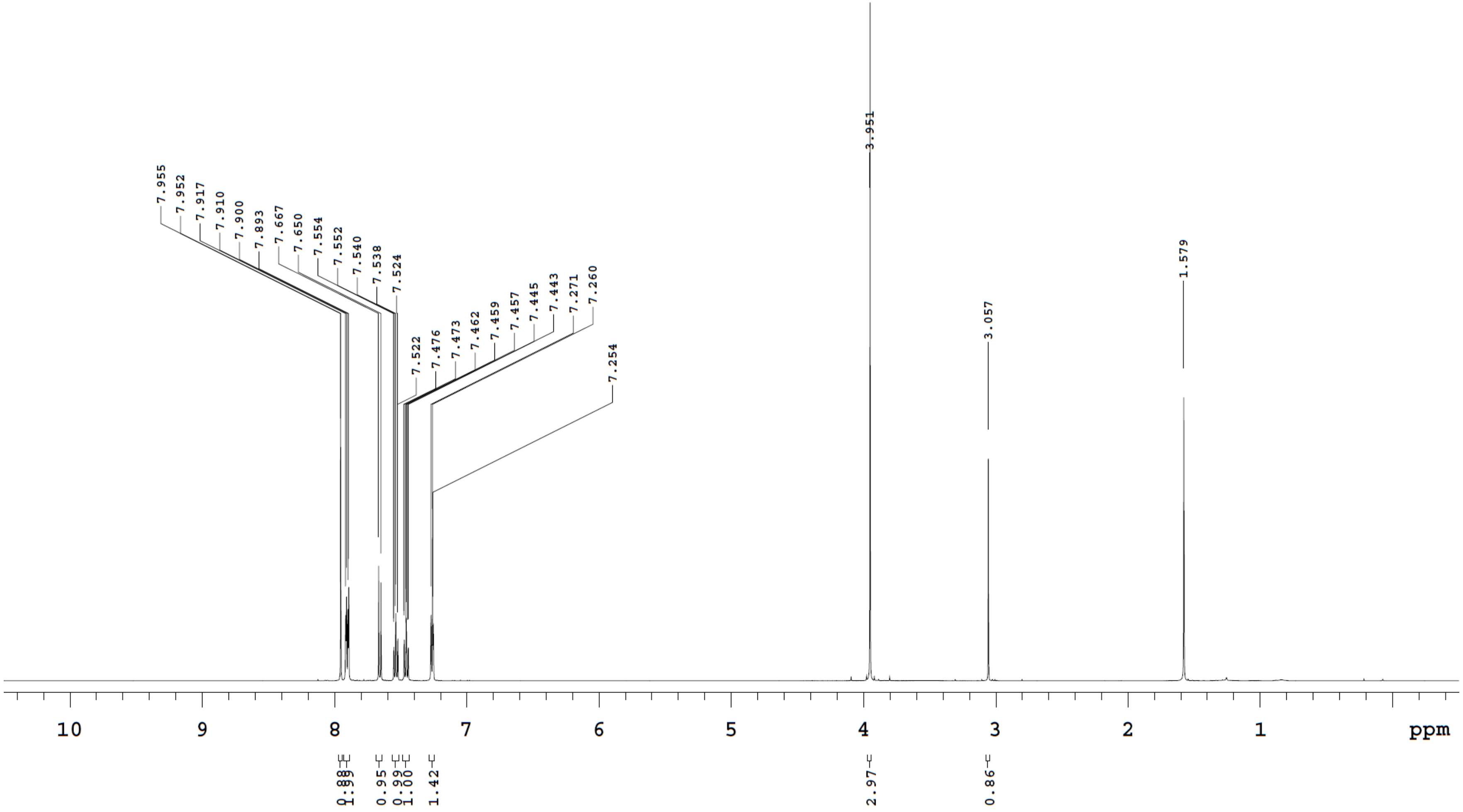

2-Ethynyl-1-(2,4,6-tribromo-3-methoxyphenyl)naphthalene

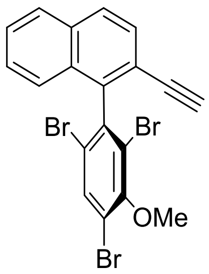

^{13}C NMR

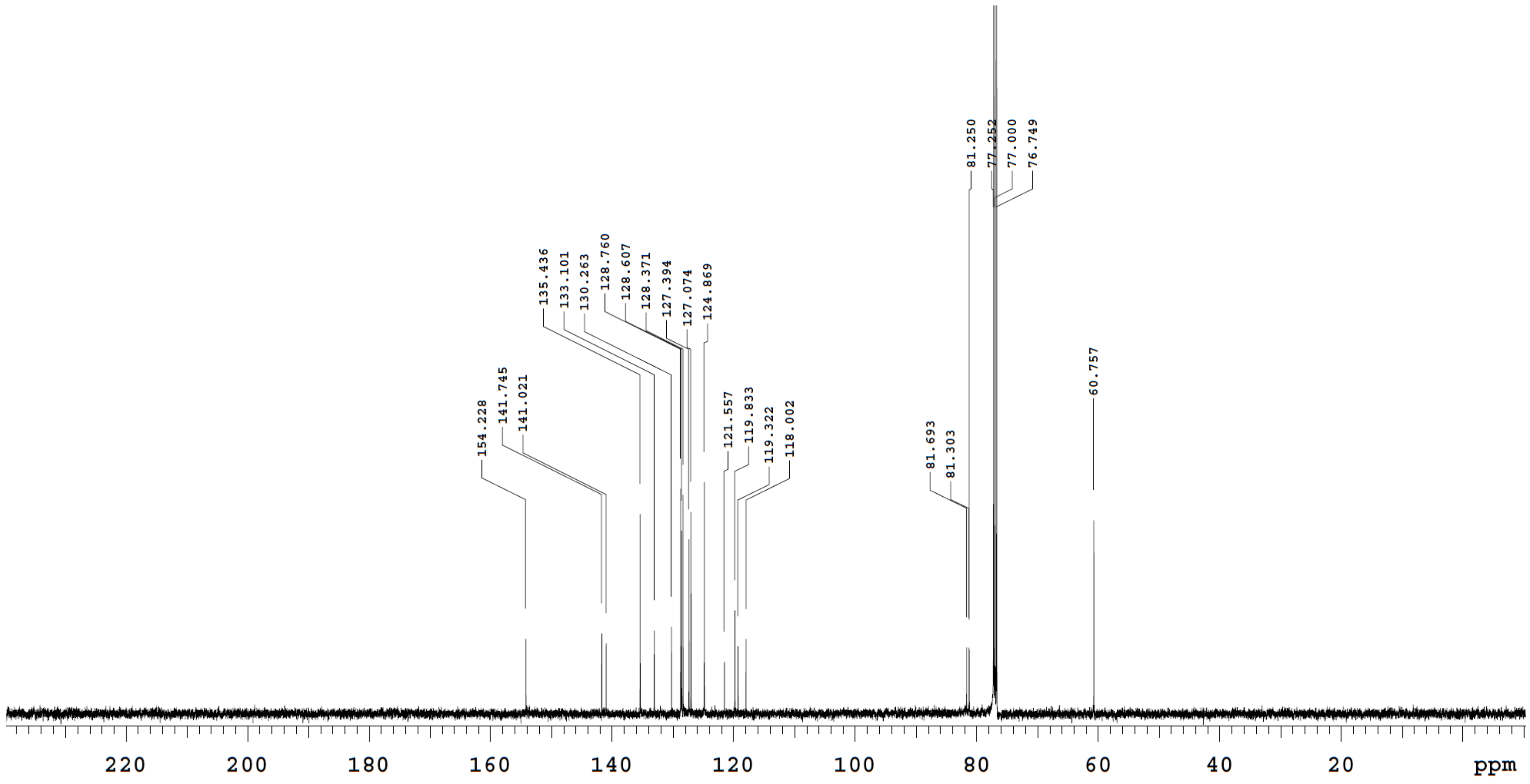

HPLC Chromatogram Profiles

1-(2,4,6-Tribromo-3-methoxyphenyl)-2-naphthonitrile (5a).

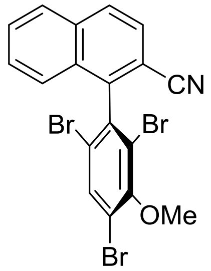

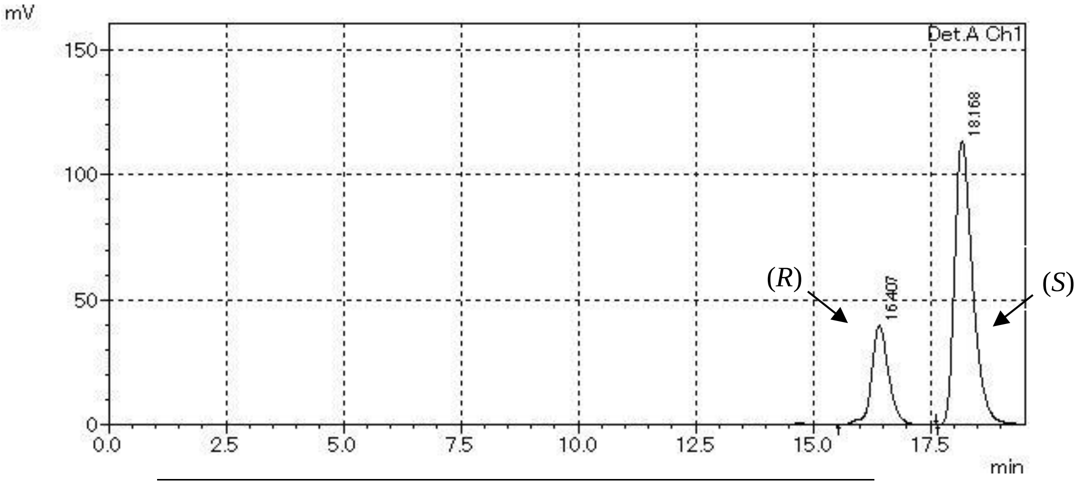

peak	retention time (min)	area %
1	16.407	24.880
2	18.168	75.120

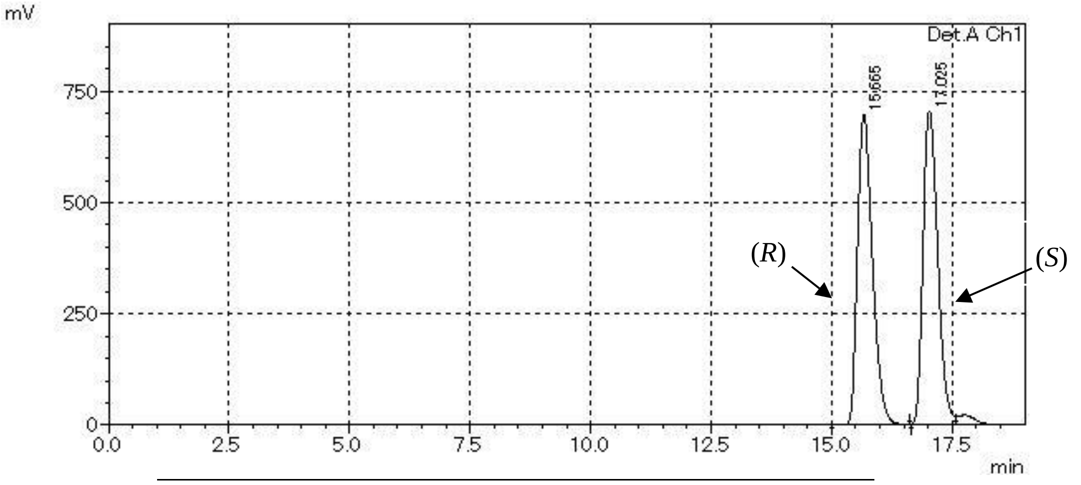

peak	retention time (min)	area %
1	15.665	50.186
2	17.025	49.814

1-(2,4,6-Tribromo-3-methoxy-5-(trifluoromethyl)phenyl)-2-naphthonitrile (5b).

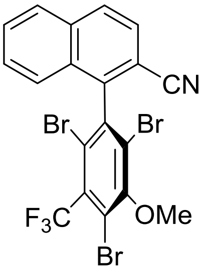

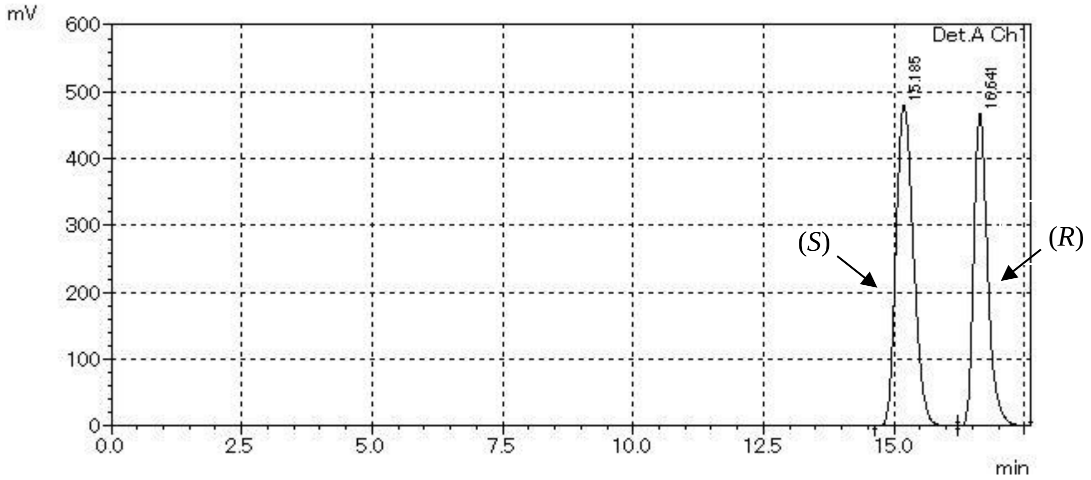

peak	retention time (min)	area %
1	15.185	56.459
2	16.641	43.541

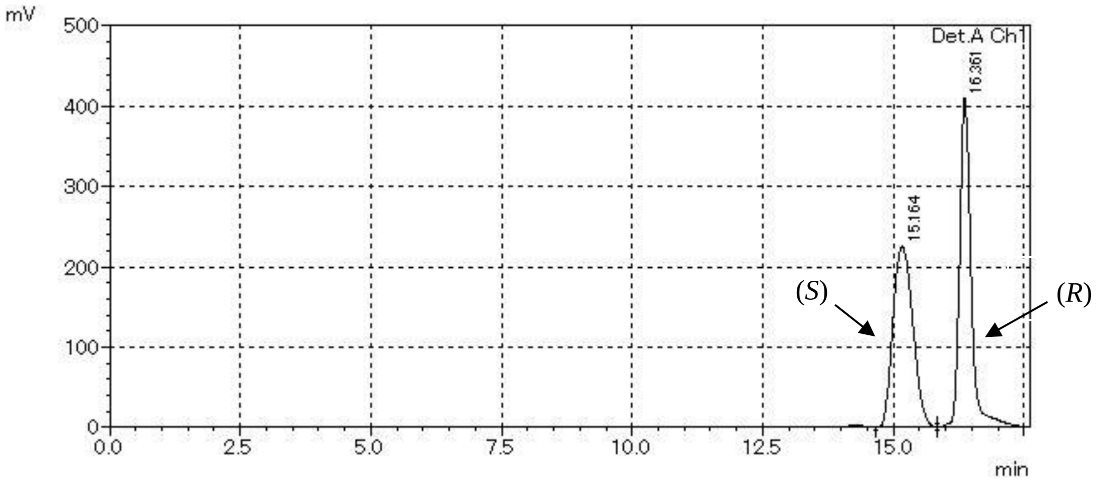

peak	retention time (min)	area %
1	15.164	48.923
2	16.361	51.077

(*S*)-1-(2,4,6-Tribromo-3-methoxy-5-methylphenyl)-2-naphthonitrile (5c).

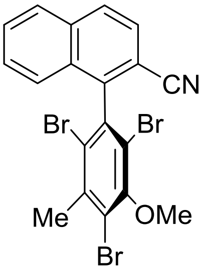

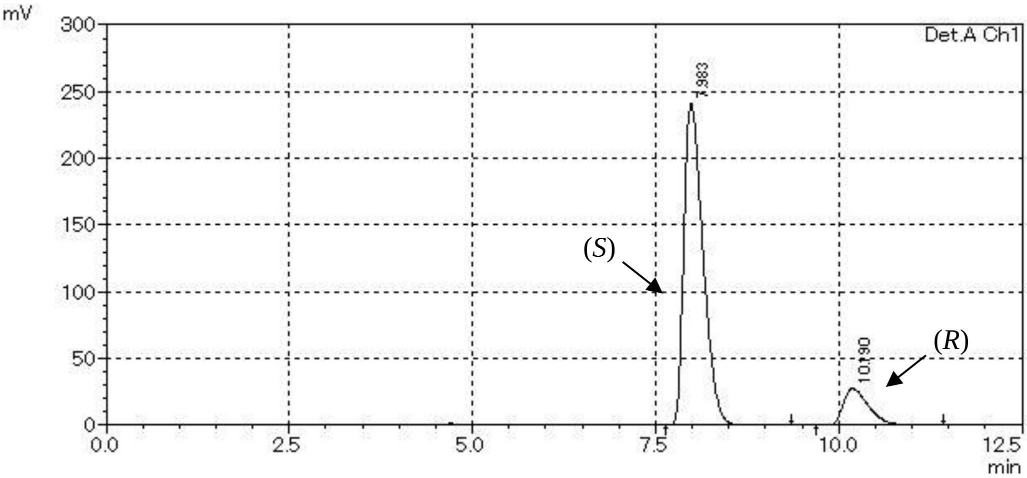

peak	retention time (min)	area %
1	7.983	86.563
2	10.190	13.437

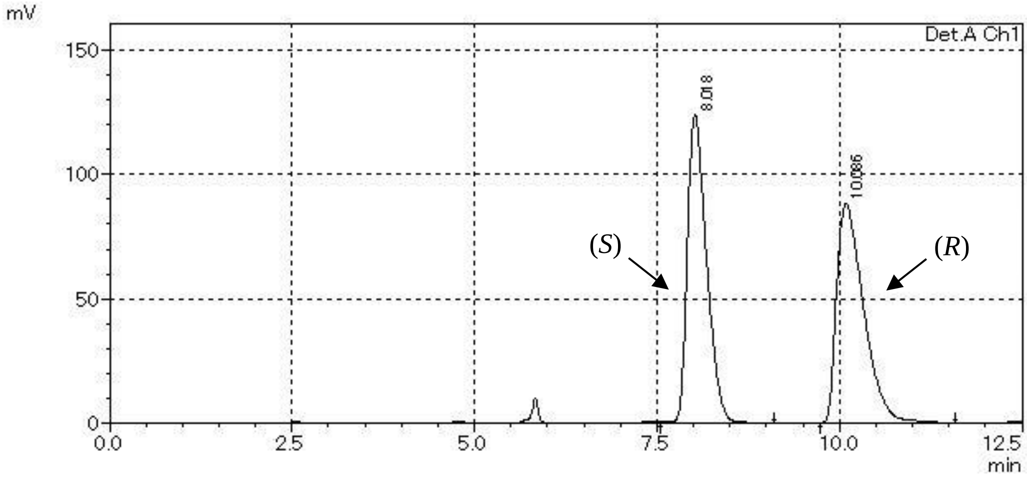

peak	retention time (min)	area %
1	8.018	49.884
2	10.086	50.116

1-(2,4,6-Tribromo-3-isopropyl-5-methoxyphenyl)-2-naphthonitrile (5d).

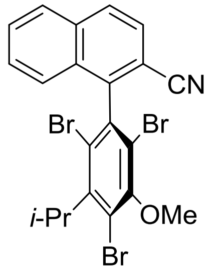

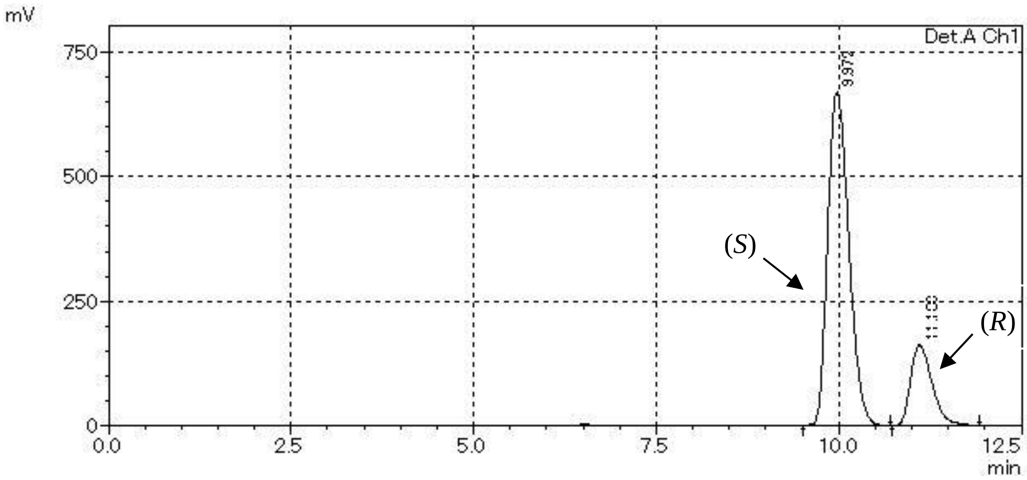

peak	retention time (min)	area %
1	9.972	80.138
2	11.103	19.862

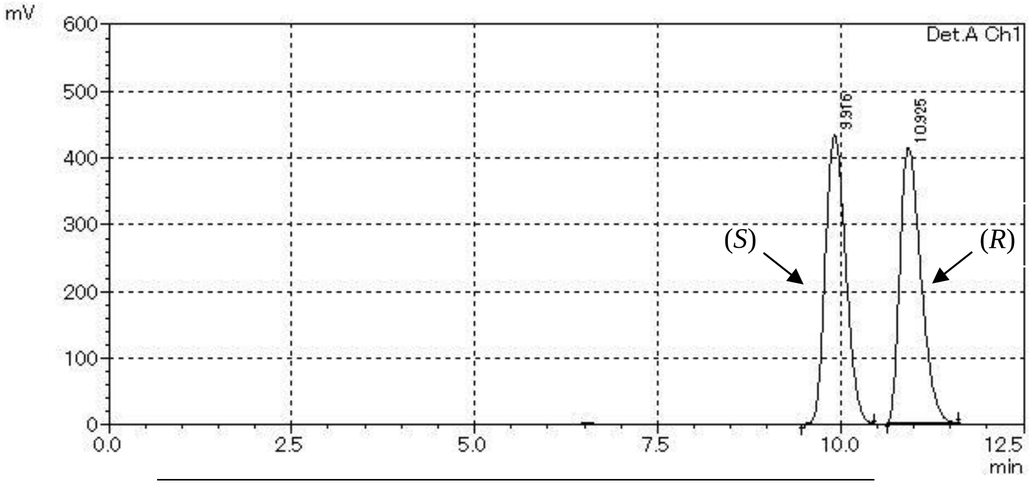

peak	retention time (min)	area %
1	9.916	50.170
2	10.925	49.830

2,4,6-Tribromo-3-(2-cyanonaphthalen-1-yl)-5-methoxyphenyl acetate (6e).

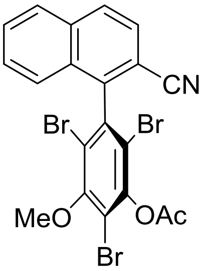

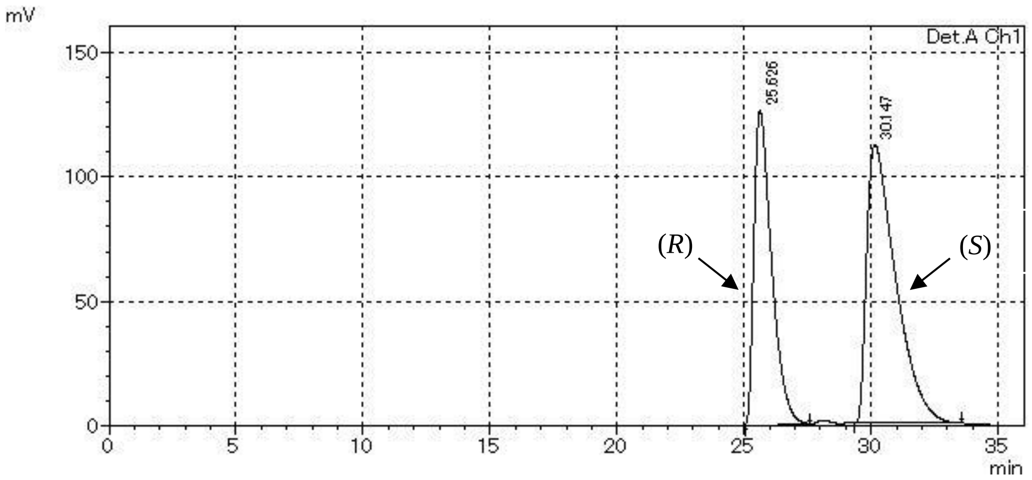

peak	retention time (min)	area %
1	25.626	40.825
2	30.147	59.175

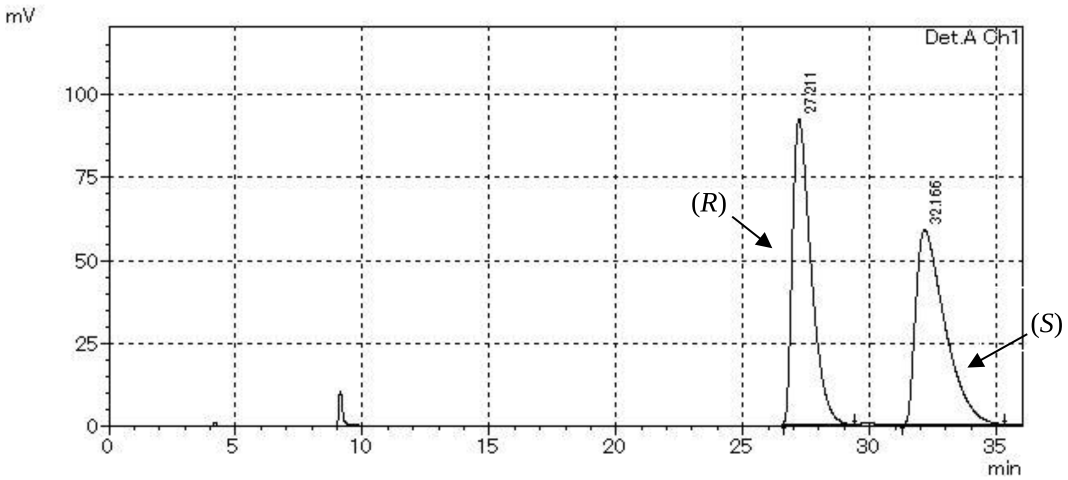

peak	retention time (min)	area %
1	27.211	50.162
2	32.166	49.838

1-(2,6-Dibromo-4-fluoro-3-methoxyphenyl)-2-naphthonitrile (5f).

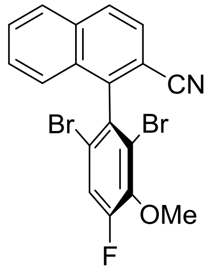

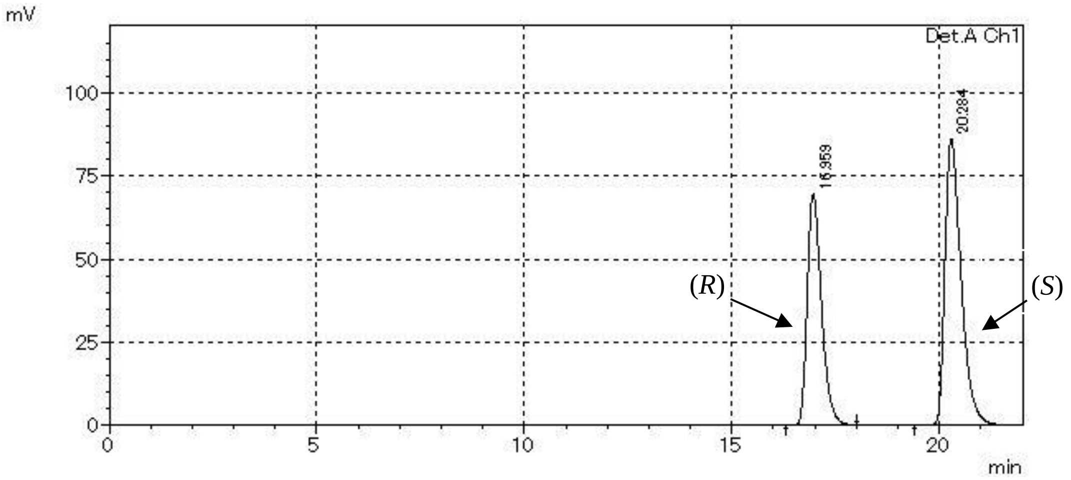

peak	retention time (min)	area %
1	16.959	41.366
2	20.284	58.634

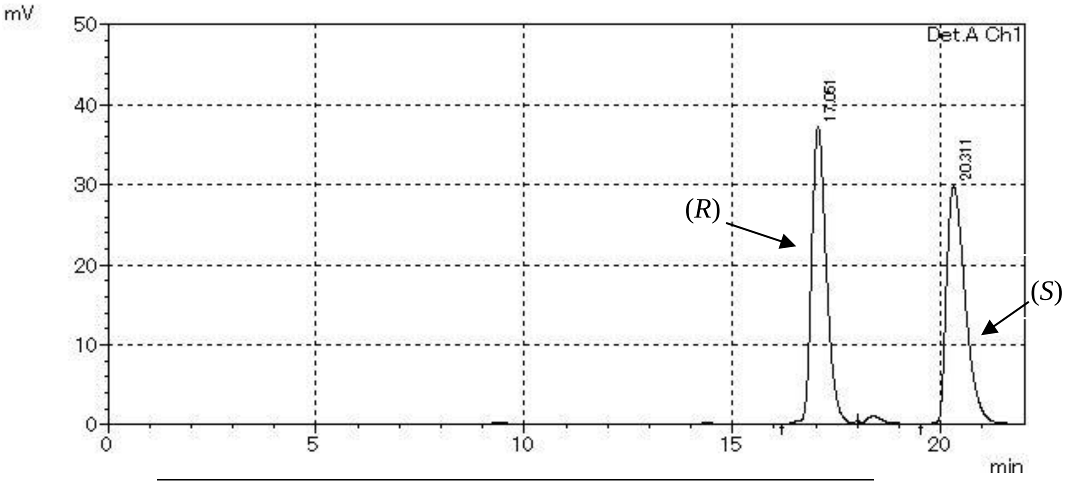

peak	retention time (min)	area %
1	17.051	49.459
2	20.311	50.541

1-(2,6-Dibromo-4-chloro-3-methoxyphenyl)-2-naphthonitrile (5g).

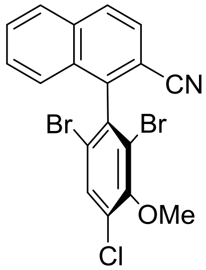

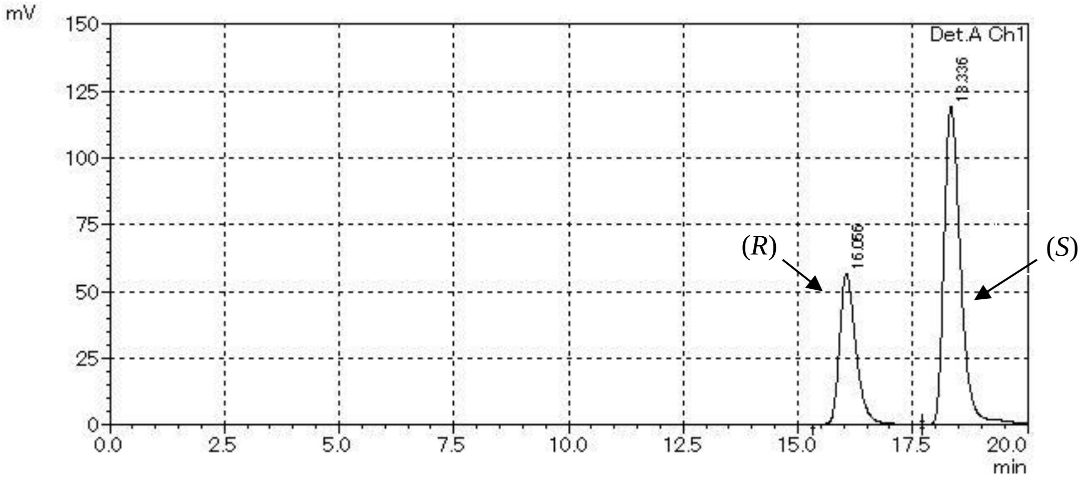

peak	retention time (min)	area %
1	16.056	32.013
2	18.336	67.987

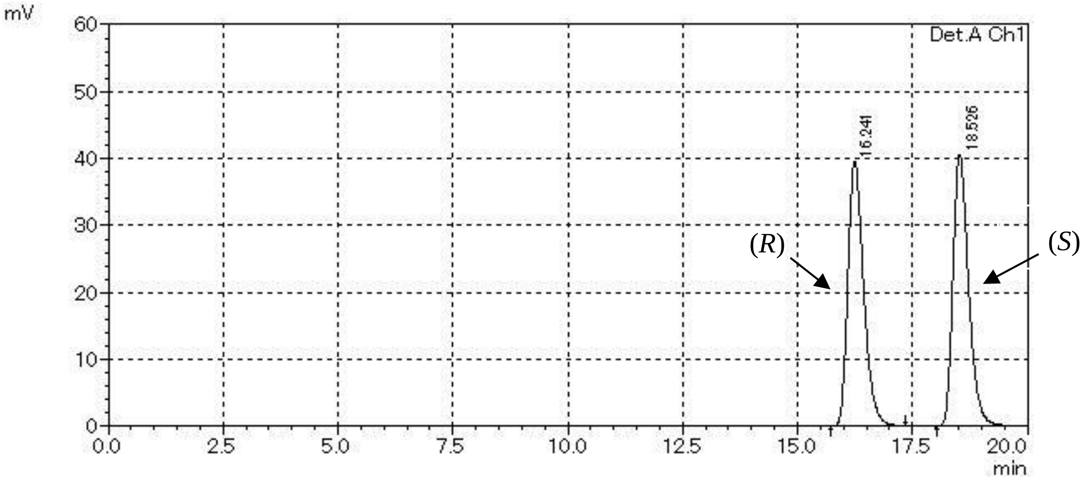

peak	retention time (min)	area %
1	16.241	49.733
2	18.526	50.582

2,4-Dibromo-3-(2-cyanonaphthalen-1-yl)-6-methylphenyl acetate (6h).

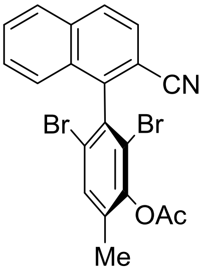

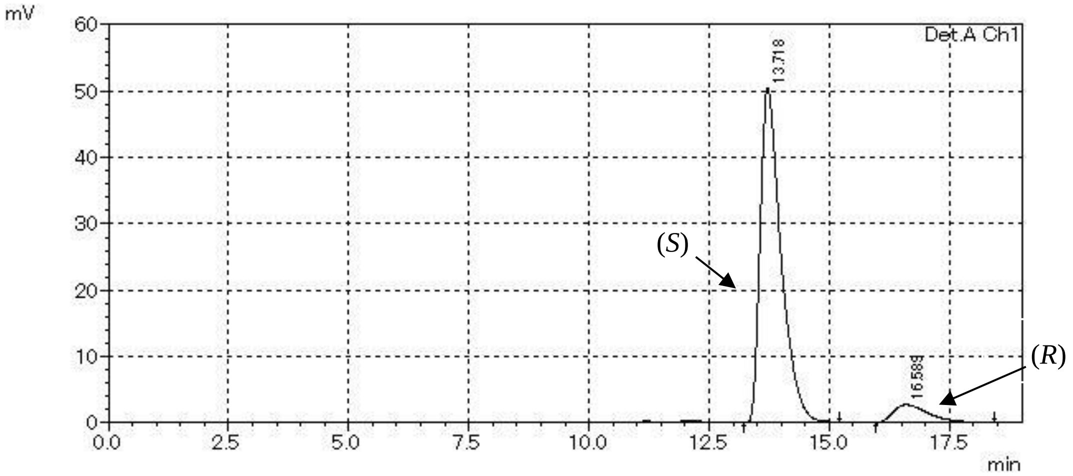

peak	retention time (min)	area %
1	13.718	91.965
2	16.589	8.035

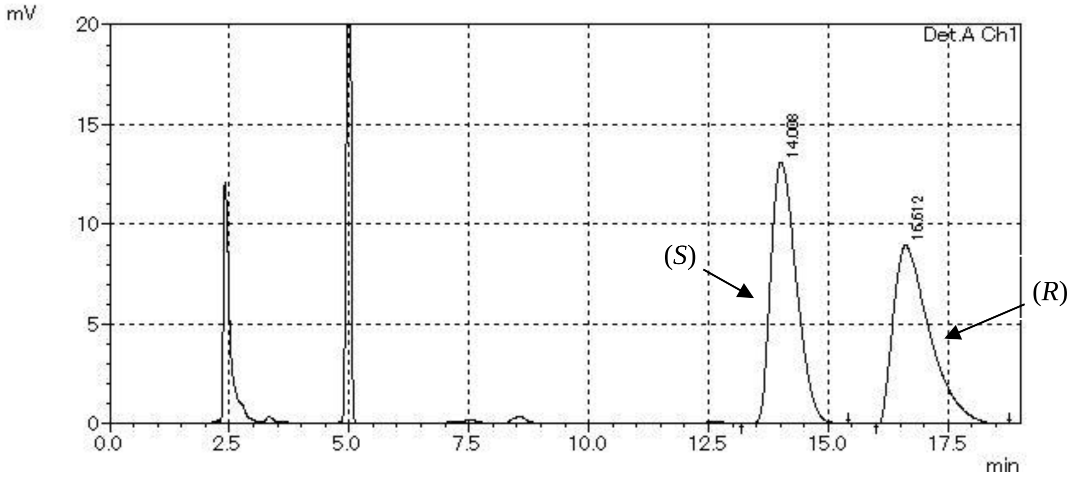

peak	retention time (min)	area %
1	14.008	50.601
2	16.612	49399

1-(2,6-Dibromo-3,4-dimethoxyphenyl)-2-naphthonitrile (5i).

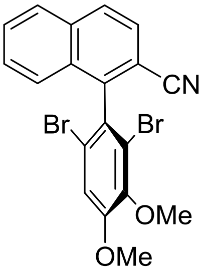

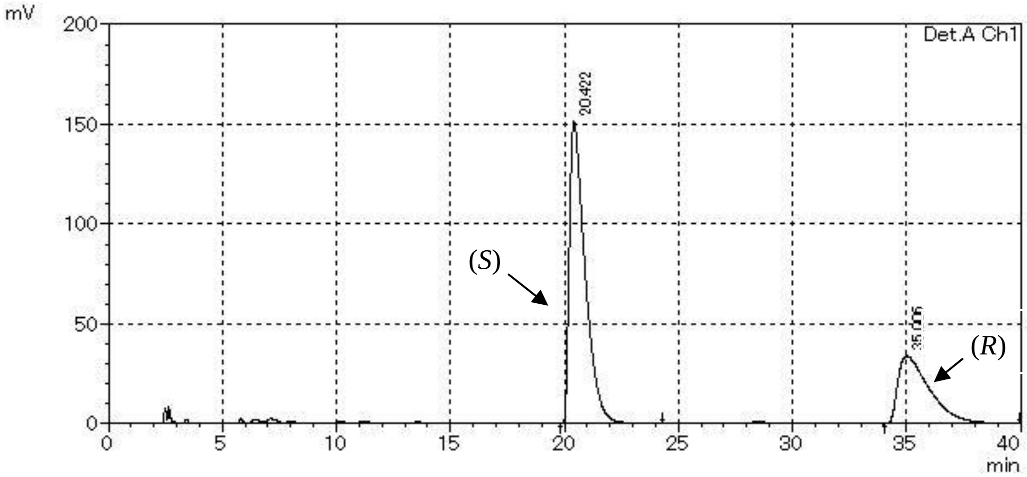

peak	retention time (min)	area %
1	20.422	69.051
2	35.006	30.949

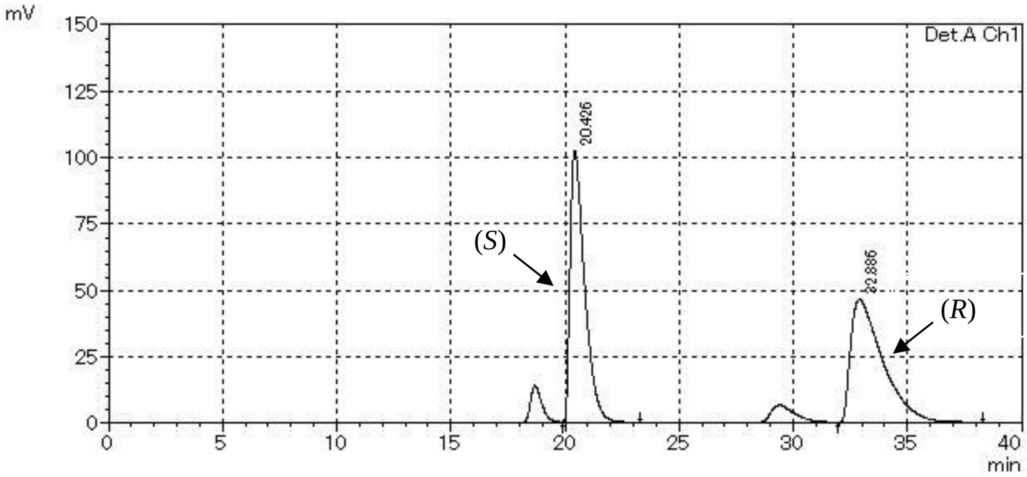

peak	retention time (min)	area %
1	20.426	49.961
2	32.886	50.039

1-(2-Bromo-3-hydroxyphenyl)-2-naphthonitrile (1a-Br) (69% *ee*).

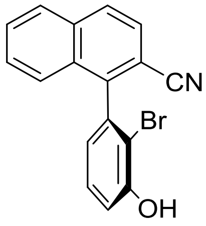

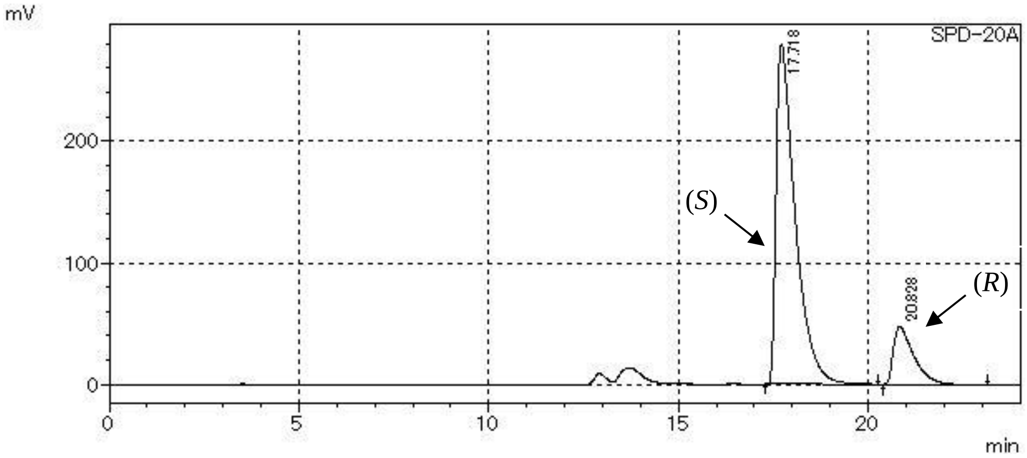

peak	retention time (min)	area %
1	17.718	84.526
2	20.828	15.474

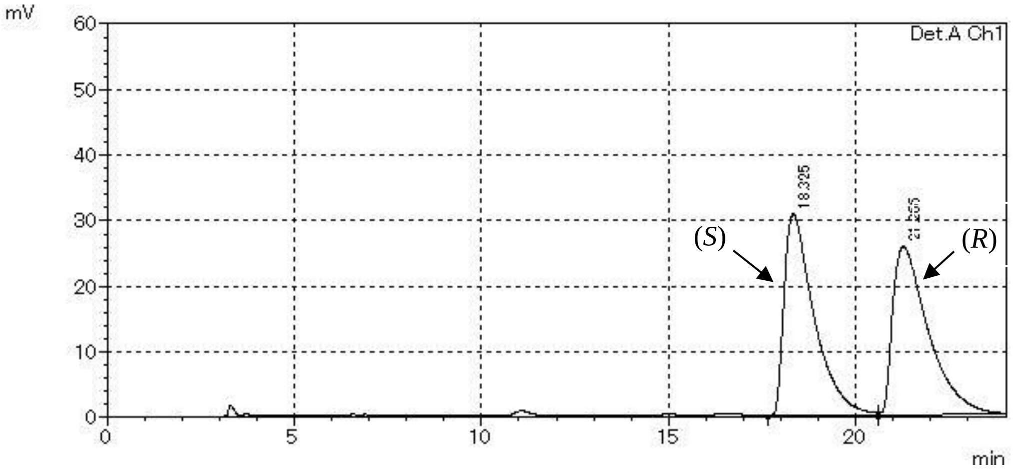

peak	retention time (min)	area %
1	18.325	50.133
2	21.266	49.867

2-Ethynyl-1-(2,4,6-tribromo-3-methoxyphenyl)naphthalene.

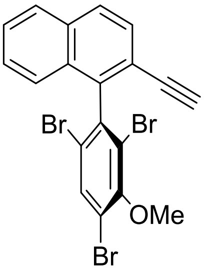

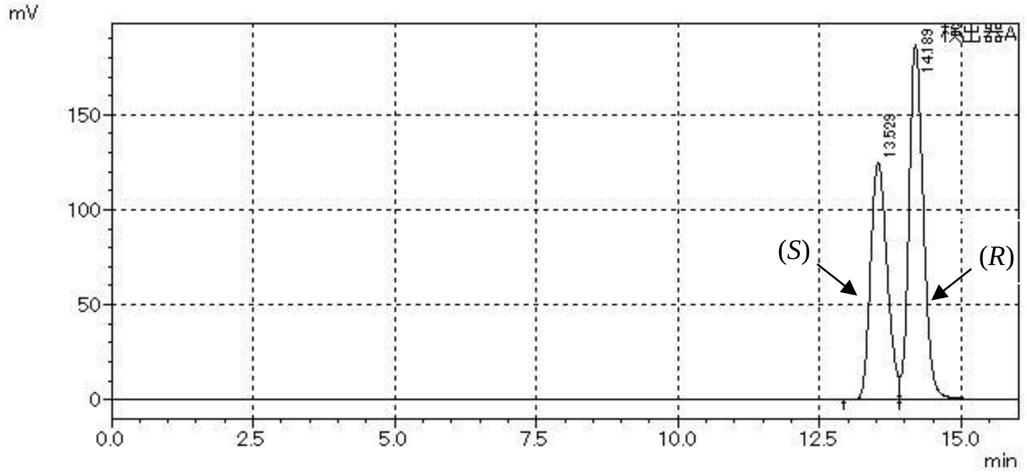

peak	retention time (min)	area %
1	13.529	43.489
2	14.189	56.511

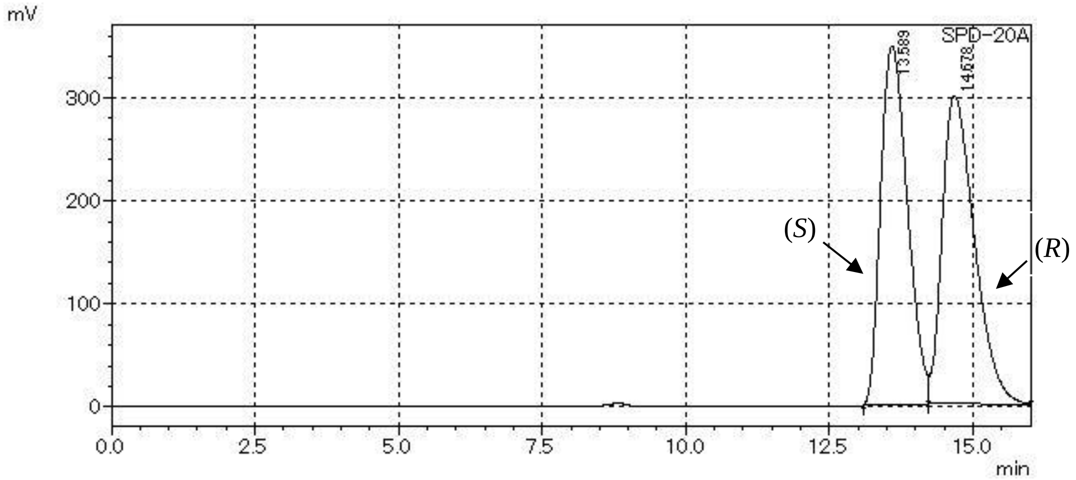

peak	retention time (min)	area %
1	13.589	49.264
2	14.678	50.736

ORTEP Drawing of 5c

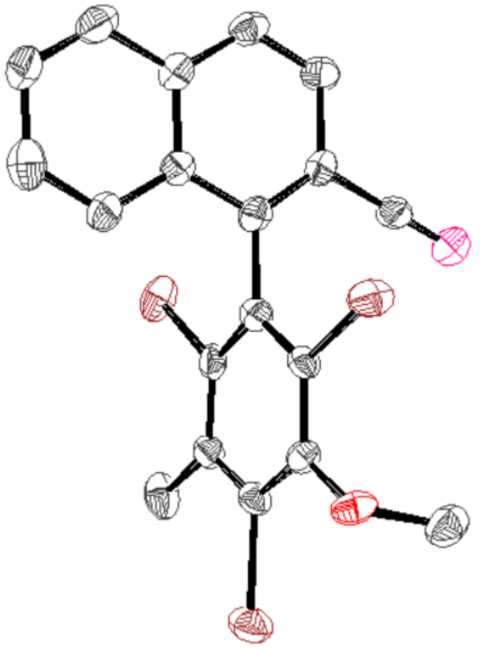

A. Crystal Data

Empirical Formula	$C_{19}H_{12}Br_3NO$
Formula Weight	510.02
Crystal Color, Habit	Colorless, Prism
Crystal Dimensions	0.390 × 0.120 × 0.090 mm
Crystal System	Monoclinic
Lattice Type	Primitive
Lattice Parameters	a = 12.330(2) Å
	b = 7.9001(9) Å
	c = 18.630(2) Å
	β = 96.052(7)°
	V = 1804.6(4) $Å^3$
Space Group	$P2_1$ (#4)
Z value	4
D_{calc}	1.877 g/cm^3
F_{000}	984.00
μ(MoKα)	67.312 cm^{-1}

B. Intensity Measurements

Diffractometer	XtaLAB mini
Radiation	MoKα (λ = 0.71075 Å)
	Graphite monochromated
Voltage, Current	50 kV, 12 mA
Temperature	20.0 °C
Detector Aperture	75 mm (diameter)
Data Images	1080 exposures
ω Oscillation Range	–60.0–120.0°
Exposure Rate	64.0 sec./°
Detector Swing Angle	30.00°
ω Oscillation Range	–60.0–120.0°
Exposure Rate	64.0 sec./°
Detector Swing Angle	30.00°
ω Oscillation Range	–60.0–120.0°
Exposure Rate	64.0 sec./°
Detector Swing Angle	30.00°
ω Oscillation Range	–60.0–120.0°
Exposure Rate	64.0 sec./°
Detector Swing Angle	30.00°
ω Oscillation Range	–60.0–120.0°
Exposure Rate	64.0 sec./°
Detector Swing Angle	30.00°
ω Oscillation Range	–60.0–120.0°
Exposure Rate	64.0 sec./°
Detector Swing Angle	30.00°
Detector Position	50.00 mm
Pixel Size	0.146 mm
$2\theta_{max}$	55.0°
No. of Reflections Measured	Total: 13812
	Unique: 7729 (R_{int} = 0.0595)
	Friedel pairs: 3336
Corrections	Lorentz-polarization
	Absorption
	(trans. factors: 0.315–0.546)

C. Structure Solution and Refinement

Structure Solution	Direct Methods (SHELX97)
Refinement	Full-matrix least-squares on F^2
Function Minimized	Σ w $(F_0^2 – F_c^2)^2$
Least Squares Weights	w = $1/[\sigma^2(F_0^2) + (0.0284\cdot P)^2$ + $0.0000\cdot P]$<br>where $P = (Max(F_0^2,0) + 2F_c^2)/3$
$2\theta_{max}$ cutoff	55.0°
Anomalous Dispersion	All non-hydrogen atoms
No. Observations (All reflections)	7729
No. Variables	433
Reflection/Parameter Ratio	17.85
Residuals: R1 (I>2.00σ(I))	0.0552
Residuals: R (All reflections)	0.1033
Residuals: wR2 (All reflections)	0.1047
Goodness of Fit Indicator	1.007
Flack Parameter (Friedel pairs = 2089)	–0.020(15)
Max Shift/Error in Final Cycle	0.001
Maximum peak in Final Diff. Map	0.46 $e^-/Å^3$
Minimum peak in Final Diff. Map	–0.38 $e^-/Å^3$

DFT Calculations of Rotational Barriers of 1a, 1a-Br, and 2a

A. Reference.

Gaussian 09, Revision B.01.
Frisch, M. J.; Trucks, G. W.; Schlegel, H. B.; Scuseria, G. E.; Robb, M. A.; Cheeseman, J. R.; Scalmani, G.; Barone, V.; Mennucci, B.; Petersson, G. A.; Nakatsuji, H.; Caricato, M.; Li, X.; Hratchian, H. P.; Izmaylov, A. F. ; Bloino, J.; Zheng, G.; Sonnenberg, J. L.; Hada, M.; Ehara, M.; Toyota, K.; Fukuda, R.; Hasegawa, J.; Ishida, M.; Nakajima, T.; Honda, Y.; Kitao, O.; Nakai, H.; Vreven, T.; Montgomery, J. A. Jr.; Peralta, J. E.; Ogliaro, F.; Bearpark, M.; Heyd, J. J.; Brothers, E.; Kudin, K. N.; Staroverov, V. N.; Keith, T.; Kobayashi, R.; Normand, J.; Raghavachari, K.; Rendell, A.; Burant, J. C.; Iyengar, S. S.; Tomasi, J.; Cossi, M.; Rega, N.; Millam, J. M.; Klene, M.; Knox, J. E.; Cross, J. B.; Bakken, V.; Adamo, C.; Jaramillo, J.; Gomperts, R.; Stratmann, R. E.; Yazyev, O.; Austin, A. J.; Cammi, R.; Pomelli, C.; Ochterski, J. W.; Martin, R. L.; Morokuma, K.; Zakrzewski, V. G.; Voth, G. A.; Salvador, P.; Dannenberg, J. J.; Dapprich, S.; Daniels, A. D.; Farkas, O.; Foresman, J. B.; Ortiz, J. V.; Cioslowski, J.; Fox, D. J., Gaussian, Inc., Wallingford CT, 2010.

Method of calculation:
Diener, M. E.; Metrano, A. J.; Kusano, S.; Miller. S. J. *J. Am. Chem. Soc.* **2015**, *137*, 12369.

B. General procedure for calculation of rotational barriers.

Geometry optimizations of **1a**, **1a**-Br, and **2a** were carried out using the B3LYP hybrid functional with the 6-31+G(d,p) basis set. Potential energy surface scan for dihedral angles of rotational axes from the obtaind structures were carried out using the same level of theory. The structures of ground states (GS) and transition states (TS) were calculated from the structure with minimum and maximum energies using the same level of theory. If the calculation of TS were difficult, QST2 method was carried out using the same level of theory. As a result, two GS and two TS for each compound were given.

Single-point calculations for all GS and TS were carried out using M06-2X hybrid functional with the 6-311++G(2d,3p) basis set.

Rotational barriers for each TS, $\Delta G^{\ddagger}$ (1) and $\Delta G^{\ddagger}$ (2), are energy differences between

GS and TS. Net rotational barrires $\Delta G^{\ddagger}$ were calculated using the forrowing equation.

$\Delta G^{\ddagger} = -RT*\ln(k_BT/k_{obs}h)$

$k_{obs} = k_1 + k_2 = k_BT/h*\{\exp(-\Delta G^{\ddagger}(1)/RT) + \exp(-\Delta G^{\ddagger}(2)/RT)\}$

R (Gas Constant): $1.99*10^{-3}$ kcal/(molK)

T (Temperature): 298.15 K

k_B (Boltzmann Constant): $1.38*10^{-23}$ $(m^2\ kg)/(s^2\ K)$

h (Planck's Constant): $6.63*10^{-34}$ $(m^2\ kg)/(s^2)$

C. Energy diagrams for rotation and rotational barriers of **1a**, **1a**-Br, and **2a**

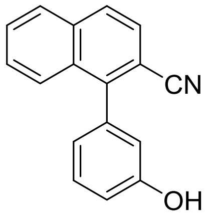

1a

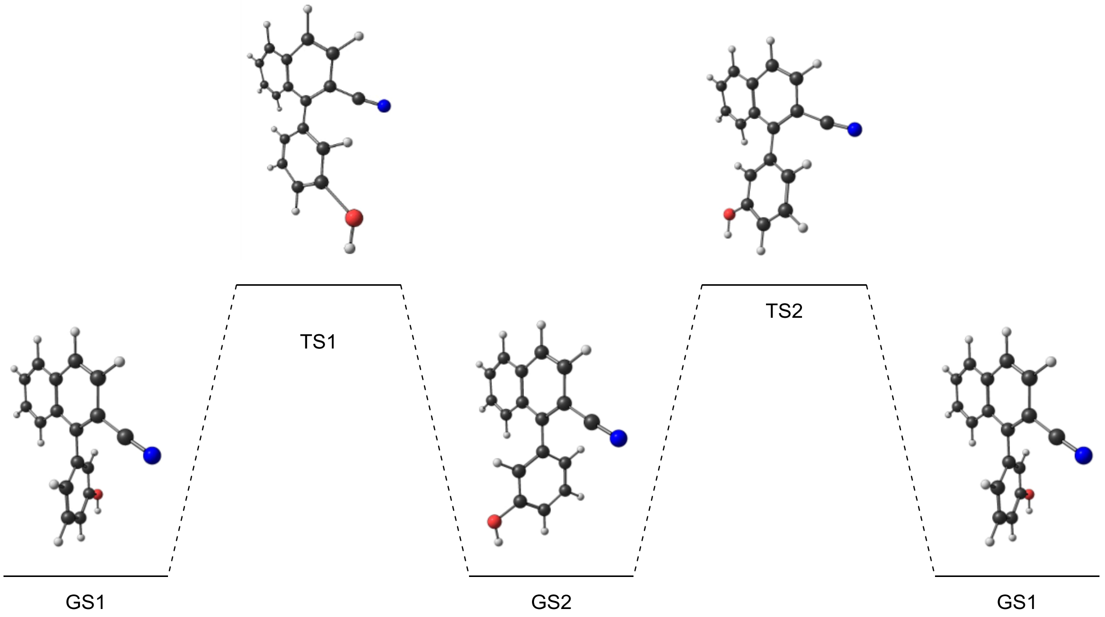

$\Delta G^{\ddagger}$ (1) = 19.3 kcal/mol

$\Delta G^{\ddagger}$ (2) = 18.6 kcal/mol

$\Delta G^{\ddagger}$ = 18.4 kcal/mol

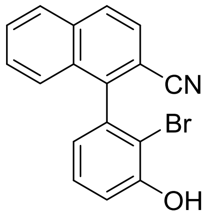

1a-Br

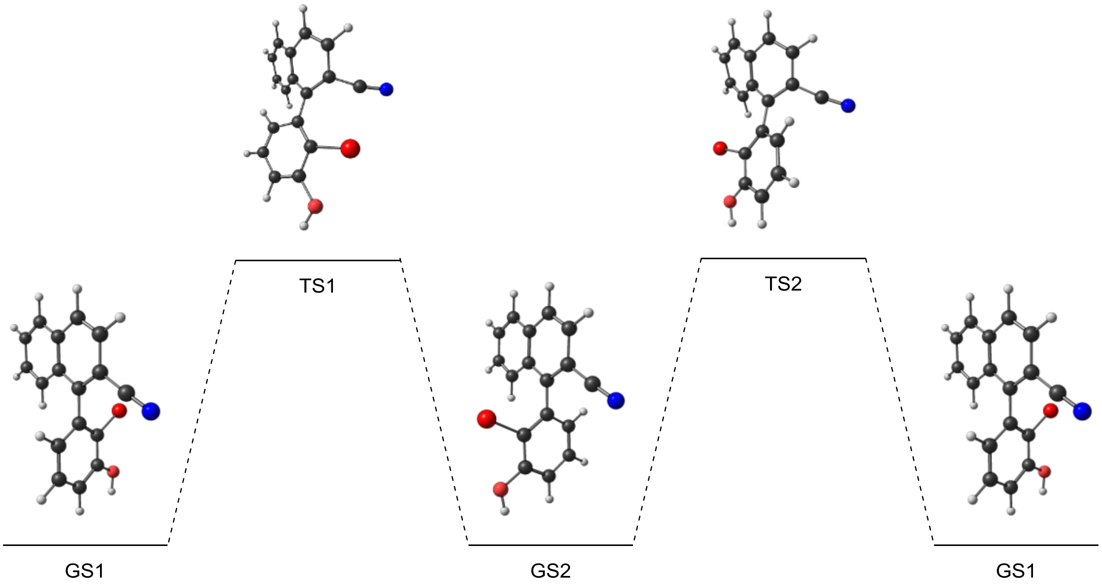

ΔG‡ (1) = 39.0 kcal/mol
ΔG‡ (2) = 40.0 kcal/mol

ΔG‡ = 38.9 kcal/mol

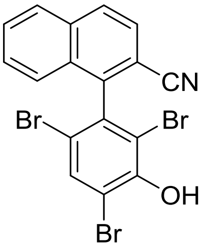

2a

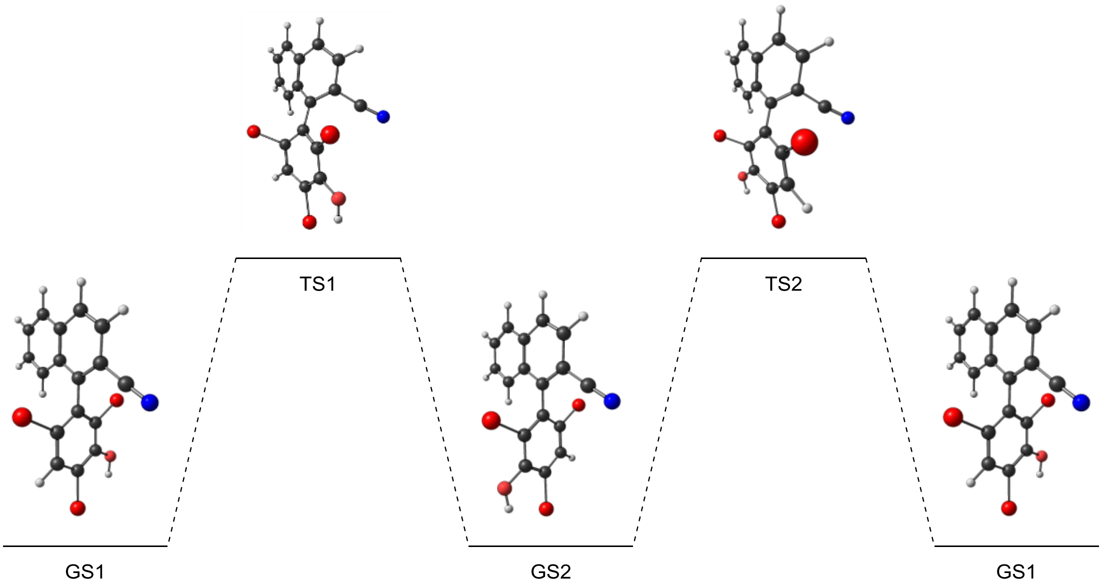

ΔG‡ (1) = 52.3 kcal/mol

ΔG‡ (2) = 52.1 kcal/mol

ΔG‡ = 51.7 kcal/mol

D. Cartesian coordinates

1a GS1

C	2.291230000000	–2.647575000000	–0.367116000000
C	1.220848000000	–1.779715000000	–0.334308000000
C	1.410295000000	–0.389340000000	–0.087215000000
C	2.750052000000	0.089698000000	0.107850000000
C	3.830002000000	–0.833080000000	0.072394000000
C	3.608818000000	–2.173465000000	–0.156676000000
H	2.123467000000	–3.703199000000	–0.558806000000
H	0.217481000000	–2.152954000000	–0.502867000000
C	0.315936000000	0.536652000000	–0.049579000000
C	2.971212000000	1.477249000000	0.323871000000
H	4.838798000000	–0.459534000000	0.226660000000
H	4.443411000000	–2.867829000000	–0.182221000000
C	1.921856000000	2.359319000000	0.344232000000
C	0.588092000000	1.889461000000	0.159464000000
H	3.987151000000	1.832793000000	0.470936000000
H	2.085910000000	3.418793000000	0.509063000000
C	–0.470511000000	2.858467000000	0.223373000000
N	–1.284609000000	3.688265000000	0.287561000000
C	–1.092324000000	0.071493000000	–0.233688000000
C	–1.716111000000	–0.685679000000	0.765817000000
C	–1.802608000000	0.402791000000	–1.398433000000
C	–3.040503000000	–1.102768000000	0.605495000000
H	–1.191511000000	–0.945439000000	1.679525000000
C	–3.122095000000	–0.023853000000	–1.551364000000
H	–1.325125000000	0.991639000000	–2.174629000000
C	–3.748327000000	–0.776235000000	–0.556057000000
H	–3.671985000000	0.234477000000	–2.451313000000
H	–4.778880000000	–1.101776000000	–0.678915000000
O	–3.589770000000	–1.833552000000	1.626517000000
H	–4.513475000000	–2.036930000000	1.429141000000

1a TS1

C	3.127784000000	–2.171037000000	0.459985000000
C	1.874006000000	–1.608440000000	0.591284000000
C	1.570079000000	–0.299343000000	0.111044000000
C	2.695513000000	0.463110000000	–0.366658000000
C	3.961390000000	–0.159001000000	–0.544938000000
C	4.176807000000	–1.463606000000	–0.165681000000
H	3.310865000000	–3.160233000000	0.869209000000
H	1.143145000000	–2.155003000000	1.164700000000
C	0.250250000000	0.321281000000	0.162066000000
C	2.568282000000	1.859717000000	–0.579277000000
H	4.772565000000	0.439073000000	–0.951289000000
H	5.151823000000	–1.923937000000	–0.292699000000
C	1.406546000000	2.488608000000	–0.229238000000
C	0.258916000000	1.734169000000	0.155643000000
H	3.420810000000	2.425700000000	–0.942438000000
H	1.321958000000	3.569270000000	–0.262801000000
C	–0.829817000000	2.570626000000	0.577767000000
N	–1.598188000000	3.357694000000	0.962620000000
C	–1.045246000000	–0.444527000000	0.099622000000
C	–2.243700000000	0.238148000000	–0.200040000000
C	–1.148295000000	–1.854345000000	0.191463000000
C	–3.469616000000	–0.419300000000	–0.309154000000
H	–2.274622000000	1.300238000000	–0.384512000000
C	–2.374528000000	–2.506367000000	0.078425000000
H	–0.283504000000	–2.480022000000	0.307541000000
C	–3.554484000000	–1.803604000000	–0.154050000000
H	–2.403448000000	–3.588771000000	0.161680000000
H	–4.507277000000	–2.320125000000	–0.241558000000
O	–4.557201000000	0.362236000000	–0.596485000000
H	–5.350712000000	–0.184481000000	–0.663880000000

1a GS2

C	2.291112000000	–2.647526000000	0.367861000000
C	1.220761000000	–1.779639000000	0.334820000000
C	1.410240000000	–0.389328000000	0.087371000000
C	2.750028000000	0.089616000000	–0.107735000000
C	3.829944000000	–0.833197000000	–0.072050000000
C	3.608717000000	–2.173517000000	0.157320000000
H	2.123304000000	–3.703094000000	0.559832000000
H	0.217389000000	–2.152813000000	0.503477000000
C	0.315907000000	0.536702000000	0.049429000000
C	2.971271000000	1.477111000000	–0.324024000000
H	4.838756000000	–0.459711000000	–0.226381000000
H	4.443287000000	–2.867904000000	0.183030000000
C	1.921960000000	2.359232000000	–0.344588000000
C	0.588161000000	1.889470000000	–0.159852000000
H	3.987236000000	1.832570000000	–0.471121000000
H	2.086075000000	3.418664000000	–0.509607000000
C	–0.470362000000	2.858554000000	–0.223974000000
N	–1.284364000000	3.688439000000	–0.288279000000
C	–1.092412000000	0.071738000000	0.233437000000
C	–1.715688000000	–0.686956000000	–0.765251000000
C	–1.803336000000	0.404813000000	1.397302000000
C	–3.040163000000	–1.103783000000	–0.604987000000
H	–1.190629000000	–0.948110000000	–1.678311000000
C	–3.122906000000	–0.021570000000	1.550186000000
H	–1.326250000000	0.994825000000	2.172870000000
C	–3.748612000000	–0.775486000000	0.555698000000
H	–3.673271000000	0.238136000000	2.449457000000
H	–4.779227000000	–1.100852000000	0.678498000000
O	–3.588894000000	–1.836125000000	–1.625195000000
H	–4.512712000000	–2.039175000000	–1.428003000000

1a TS2

C	2.317654000000	–2.661218000000	0.500235000000
C	1.267432000000	–1.769788000000	0.589749000000
C	1.361136000000	–0.428650000000	0.110676000000
C	2.672504000000	–0.013129000000	–0.318655000000
C	3.720179000000	–0.964689000000	–0.452527000000
C	3.546733000000	–2.276652000000	–0.077248000000
H	2.198956000000	–3.661943000000	0.904913000000
H	0.392070000000	–2.091388000000	1.129635000000
C	0.266266000000	0.536561000000	0.114573000000
C	2.948846000000	1.361988000000	–0.529399000000
H	4.681582000000	–0.618410000000	–0.822142000000
H	4.358148000000	–2.991848000000	–0.171075000000
C	1.995534000000	2.292919000000	–0.226099000000
C	0.668699000000	1.891425000000	0.109477000000
H	3.939504000000	1.664828000000	–0.854935000000
H	2.217133000000	3.353957000000	–0.260511000000
C	–0.160027000000	3.003781000000	0.482705000000
N	–0.695916000000	3.979674000000	0.827353000000
C	–1.189020000000	0.166239000000	0.004903000000
C	–1.678936000000	–1.153358000000	0.096599000000
C	–2.146847000000	1.152315000000	–0.338744000000
C	–3.034446000000	–1.453654000000	–0.053123000000
H	–1.042843000000	–2.005970000000	0.245240000000
C	–3.496704000000	0.848367000000	–0.484339000000
H	–1.857974000000	2.173214000000	–0.526146000000
C	–3.967172000000	–0.454757000000	–0.326940000000
H	–4.190477000000	1.643657000000	–0.739172000000
H	–5.022988000000	–0.688335000000	–0.440933000000
O	–3.378114000000	–2.776924000000	0.074615000000
H	–4.327755000000	–2.886297000000	–0.064322000000

1a-Br GS1

C	–2.587136000000	–2.472039000000	0.983841000000
C	–1.532493000000	–1.589247000000	0.904715000000
C	–1.724573000000	–0.263288000000	0.423451000000
C	–3.038825000000	0.130126000000	0.003865000000
C	–4.101795000000	–0.809248000000	0.087398000000
C	–3.883374000000	–2.082877000000	0.565478000000
H	–2.422816000000	–3.477914000000	1.358482000000
H	–0.542788000000	–1.897645000000	1.222799000000
C	–0.653986000000	0.682433000000	0.357062000000
C	–3.249848000000	1.452279000000	–0.474859000000
H	–5.094014000000	–0.503667000000	–0.233692000000
H	–4.704927000000	–2.790421000000	0.626810000000
C	–2.216845000000	2.352776000000	–0.533977000000
C	–0.910361000000	1.968336000000	–0.112889000000
H	–4.246811000000	1.744394000000	–0.792652000000
H	–2.375536000000	3.362472000000	–0.896933000000
C	0.138139000000	2.947482000000	–0.177309000000
N	0.954476000000	3.775925000000	–0.228514000000
C	0.717443000000	0.315662000000	0.826652000000
C	1.633116000000	–0.327931000000	–0.015898000000
C	1.100709000000	0.623182000000	2.140362000000
C	2.924076000000	–0.640173000000	0.436866000000
C	2.380190000000	0.309551000000	2.594941000000
H	0.390790000000	1.118597000000	2.794407000000
C	3.291241000000	–0.318282000000	1.747415000000
H	2.673113000000	0.559204000000	3.609789000000
H	4.292828000000	–0.558759000000	2.096434000000
O	3.777637000000	–1.250564000000	–0.433071000000
H	4.632067000000	–1.399239000000	–0.007019000000
Br	1.165188000000	–0.750184000000	–1.807341000000

1a-Br TS1

C	–3.842150000000	1.832423000000	0.875839000000
C	–2.532296000000	1.399179000000	0.831107000000
C	–2.144935000000	0.237305000000	0.097837000000
C	–3.211305000000	–0.542562000000	–0.463111000000
C	–4.538344000000	–0.033137000000	–0.472000000000
C	–4.853094000000	1.143889000000	0.168699000000
H	–4.095965000000	2.701065000000	1.476165000000
H	–1.790262000000	1.924311000000	1.419265000000
C	–0.760855000000	–0.243433000000	0.033504000000
C	–2.945972000000	–1.866717000000	–0.900177000000
H	–5.313188000000	–0.626320000000	–0.949983000000
H	–5.875093000000	1.510261000000	0.179018000000
C	–1.746435000000	–2.433548000000	–0.570003000000
C	–0.671422000000	–1.658637000000	–0.031065000000
H	–3.732036000000	–2.451907000000	–1.367608000000
H	–1.578664000000	–3.497540000000	–0.697544000000
C	0.274160000000	–2.491298000000	0.653462000000
N	0.785527000000	–3.295348000000	1.326294000000
C	0.356567000000	0.760781000000	–0.038548000000
C	1.767326000000	0.553831000000	–0.018574000000
C	–0.026140000000	2.107081000000	–0.303987000000
C	2.667226000000	1.642369000000	–0.015956000000
C	0.861468000000	3.169900000000	–0.348807000000
H	–1.056786000000	2.323021000000	–0.529195000000
C	2.219771000000	2.954006000000	–0.148529000000
H	0.489110000000	4.169206000000	–0.550640000000
H	2.931270000000	3.775935000000	–0.158810000000
O	4.000339000000	1.362636000000	0.078205000000
H	4.505154000000	2.186327000000	0.062518000000
Br	2.650675000000	–1.115332000000	–0.139744000000

1a-Br GS2

C	–2.587293000000	2.472115000000	0.983673000000
C	–1.532601000000	1.589396000000	0.904485000000
C	–1.724634000000	0.263396000000	0.423275000000
C	–3.038880000000	–0.130092000000	0.003744000000
C	–4.101891000000	0.809245000000	0.087266000000
C	–3.883516000000	2.082890000000	0.565301000000
H	–2.423034000000	3.478004000000	1.358307000000
H	–0.542907000000	1.897800000000	1.222574000000
C	–0.654031000000	–0.682319000000	0.356978000000
C	–3.249873000000	–1.452272000000	–0.474897000000
H	–5.094105000000	0.503614000000	–0.233798000000
H	–4.705101000000	2.790396000000	0.626643000000
C	–2.216876000000	–2.352795000000	–0.533836000000
C	–0.910412000000	–1.968314000000	–0.112745000000
H	–4.246827000000	–1.744408000000	–0.792693000000
H	–2.375561000000	–3.362518000000	–0.896719000000
C	0.138136000000	–2.947407000000	–0.177057000000
N	0.954501000000	–3.775835000000	–0.228088000000
C	0.717367000000	–0.315580000000	0.826650000000
C	1.633134000000	0.327905000000	–0.015891000000
C	1.100559000000	–0.623075000000	2.140385000000
C	2.924040000000	0.640248000000	0.437003000000
C	2.379964000000	–0.309339000000	2.595074000000
H	0.390535000000	–1.118406000000	2.794385000000
C	3.291058000000	0.318531000000	1.747614000000
H	2.672810000000	–0.558893000000	3.609969000000
H	4.292571000000	0.559146000000	2.096754000000
O	3.777644000000	1.250628000000	–0.432901000000
H	4.632229000000	1.398841000000	–0.006999000000
Br	1.165464000000	0.749916000000	–1.807449000000

1a-Br TS2

C	2.327017000000	–2.610266000000	0.619694000000
C	1.439876000000	–1.555034000000	0.701299000000
C	1.689070000000	–0.312025000000	0.064937000000
C	3.013942000000	–0.134480000000	–0.471523000000
C	3.873279000000	–1.256356000000	–0.623036000000
C	3.527812000000	–2.487561000000	–0.111554000000
H	2.105289000000	–3.533398000000	1.147156000000
H	0.575361000000	–1.653059000000	1.334634000000
C	0.771431000000	0.823069000000	0.083127000000
C	3.517180000000	1.174885000000	–0.688572000000
H	4.843533000000	–1.101923000000	–1.087181000000
H	4.202631000000	–3.333066000000	–0.205074000000
C	2.791947000000	2.242862000000	–0.233779000000
C	1.438877000000	2.072405000000	0.181997000000
H	4.516675000000	1.307411000000	–1.091584000000
H	3.215420000000	3.240932000000	–0.205911000000
C	0.862428000000	3.251332000000	0.766900000000
N	0.531133000000	4.230144000000	1.306458000000
C	–0.730824000000	0.835733000000	–0.111215000000
C	–1.732219000000	–0.182405000000	–0.021333000000
C	–1.234169000000	2.092968000000	–0.548687000000
C	–3.104382000000	0.141292000000	–0.112232000000
C	–2.579632000000	2.402870000000	–0.665288000000
H	–0.540474000000	2.861556000000	–0.845389000000
C	–3.535912000000	1.434560000000	–0.401144000000
H	–2.872942000000	3.399355000000	–0.978486000000
H	–4.598626000000	1.651479000000	–0.474320000000
O	–4.013222000000	–0.867455000000	0.047541000000
H	–4.906715000000	–0.504242000000	–0.011716000000
Br	–1.490475000000	–2.074557000000	0.066136000000

2a GS1

C	2.957757000000	–0.140397000000	–2.994408000000
C	2.031618000000	–0.080332000000	–1.976957000000
C	2.446053000000	–0.068969000000	–0.615852000000
C	3.848431000000	–0.126060000000	–0.322111000000
C	4.776585000000	–0.191075000000	–1.396143000000
C	4.343353000000	–0.197340000000	–2.704000000000
H	2.625544000000	–0.146787000000	–4.028134000000
H	0.973830000000	–0.038162000000	–2.213281000000
C	1.513121000000	0.001292000000	0.461386000000
C	4.274734000000	–0.116902000000	1.034219000000
H	5.837809000000	–0.234818000000	–1.166620000000
H	5.062512000000	–0.245724000000	–3.516176000000
C	3.367147000000	–0.048818000000	2.061623000000
C	1.974055000000	0.009651000000	1.774511000000
H	5.338382000000	–0.162091000000	1.250716000000
H	3.692506000000	–0.040667000000	3.096137000000
C	1.043096000000	0.081999000000	2.864281000000
N	0.311356000000	0.143425000000	3.767803000000
C	0.047853000000	0.080665000000	0.192596000000
C	–0.731617000000	–1.079706000000	0.090077000000
C	–0.595924000000	1.317986000000	0.043489000000
C	–2.117306000000	–1.029503000000	–0.126502000000
C	–1.966493000000	1.404893000000	–0.174394000000
C	–2.708818000000	0.234013000000	–0.253109000000
H	–2.449209000000	2.368079000000	–0.279176000000
O	–2.807447000000	–2.187524000000	–0.204683000000
H	–3.749631000000	–1.992602000000	–0.341310000000
Br	0.072086000000	–2.785388000000	0.253782000000
Br	0.400552000000	2.934685000000	0.139593000000
Br	–4.589025000000	0.327291000000	–0.544562000000

2a TS1

C	–3.906774000000	–2.284427000000	–0.958371000000
C	–2.727153000000	–1.572742000000	–0.856793000000
C	–2.700282000000	–0.239917000000	–0.371518000000
C	–3.973517000000	0.391902000000	–0.158026000000
C	–5.159982000000	–0.390506000000	–0.167096000000
C	–5.129144000000	–1.717176000000	–0.537871000000
H	–3.889986000000	–3.286825000000	–1.375629000000
H	–1.822219000000	–2.011166000000	–1.251215000000
C	–1.485935000000	0.562496000000	–0.283126000000
C	–4.056109000000	1.809265000000	–0.126103000000
H	–6.103914000000	0.100342000000	0.053444000000
H	–6.044819000000	–2.299210000000	–0.577403000000
C	–2.959375000000	2.544154000000	–0.498738000000
C	–1.688325000000	1.922351000000	–0.631013000000
H	–5.014740000000	2.288095000000	0.049442000000
H	–3.034715000000	3.608248000000	–0.694061000000
C	–0.697345000000	2.710725000000	–1.302134000000
N	–0.007834000000	3.365006000000	–1.975879000000
C	–0.126121000000	0.069473000000	0.023102000000
C	0.961320000000	0.983606000000	0.235571000000
C	0.296476000000	–1.298489000000	0.119434000000
C	2.297090000000	0.637314000000	–0.018188000000
C	1.609639000000	–1.671731000000	–0.138712000000
C	2.585465000000	–0.699673000000	–0.315946000000
H	1.882845000000	–2.719443000000	–0.125838000000
O	3.251730000000	1.586706000000	0.100235000000
H	4.115929000000	1.200928000000	–0.118748000000
Br	0.764965000000	2.614840000000	1.222220000000
Br	–0.704237000000	–2.667607000000	1.020750000000
Br	4.371073000000	–1.194931000000	–0.729185000000

2a GS2

C	–2.957271000000	–0.139243000000	–2.994687000000
C	–2.031277000000	–0.079859000000	–1.977064000000
C	–2.445922000000	–0.068982000000	–0.616022000000
C	–3.848351000000	–0.125902000000	–0.322518000000
C	–4.776357000000	–0.190211000000	–1.396720000000
C	–4.342925000000	–0.195994000000	–2.704516000000
H	–2.624905000000	–0.145218000000	–4.028366000000
H	–0.973441000000	–0.037688000000	–2.213177000000
C	–1.513139000000	0.000726000000	0.461377000000
C	–4.274855000000	–0.117252000000	1.033757000000
H	–5.837626000000	–0.233819000000	–1.167379000000
H	–5.061974000000	–0.243816000000	–3.516822000000
C	–3.367402000000	–0.049816000000	2.061324000000
C	–1.974260000000	0.008596000000	1.774440000000
H	–5.338543000000	–0.162349000000	1.250079000000
H	–3.692906000000	–0.042088000000	3.095796000000
C	–1.043453000000	0.080525000000	2.864372000000
N	–0.311821000000	0.141625000000	3.768003000000
C	–0.047856000000	0.080361000000	0.192768000000
C	0.731804000000	–1.079845000000	0.089840000000
C	0.595682000000	1.317843000000	0.044023000000
C	2.117466000000	–1.029330000000	–0.126865000000
C	1.966223000000	1.405062000000	–0.173872000000
C	2.708745000000	0.234338000000	–0.253058000000
H	2.448766000000	2.368371000000	–0.278333000000
O	2.807799000000	–2.187206000000	–0.205549000000
H	3.749923000000	–1.992084000000	–0.342293000000
Br	–0.071594000000	–2.785669000000	0.253264000000
Br	–0.401042000000	2.934360000000	0.140689000000
Br	4.588920000000	0.328134000000	–0.544541000000

2a TS2

C	3.786949000000	–2.520138000000	0.747353000000
C	2.650693000000	–1.734622000000	0.754342000000
C	2.680096000000	–0.377755000000	0.342532000000
C	3.981087000000	0.196724000000	0.127946000000
C	5.118982000000	–0.649841000000	0.033747000000
C	5.023167000000	–1.995796000000	0.311673000000
H	3.728286000000	–3.544640000000	1.102682000000
H	1.743721000000	–2.140548000000	1.173689000000
C	1.513121000000	0.495386000000	0.328137000000
C	4.146122000000	1.606032000000	0.188891000000
H	6.081582000000	–0.197864000000	–0.189087000000
H	5.902728000000	–2.630944000000	0.269444000000
C	3.101561000000	2.377769000000	0.628904000000
C	1.799173000000	1.820555000000	0.741773000000
H	5.127797000000	2.038943000000	0.021401000000
H	3.240523000000	3.422417000000	0.884446000000
C	0.848875000000	2.647148000000	1.429302000000
N	0.195960000000	3.335538000000	2.105444000000
C	0.106161000000	0.157040000000	–0.006132000000
C	–0.516217000000	–1.134251000000	–0.063226000000
C	–0.824754000000	1.214242000000	–0.281103000000
C	–1.890237000000	–1.305225000000	0.176502000000
C	–2.190796000000	1.074390000000	–0.083053000000
C	–2.706205000000	–0.169903000000	0.248774000000
H	–2.850533000000	1.919442000000	–0.231539000000
O	–2.387884000000	–2.563579000000	0.238990000000
H	–3.348574000000	–2.517391000000	0.377781000000
Br	0.295244000000	–2.678107000000	–0.843218000000
Br	–0.351654000000	2.792338000000	–1.280655000000
Br	–4.562472000000	–0.382804000000	0.586244000000
